# Synthesis, Bacteriostatic and Anticancer Activity of Novel Phenanthridines Structurally Similar to Benzo[*c*]phenanthridine Alkaloids

**DOI:** 10.3390/molecules23092155

**Published:** 2018-08-27

**Authors:** Pavel Lasák, Kamil Motyka, Vladimír Kryštof, Jakub Stýskala

**Affiliations:** 1 Department of Organic Chemistry, Faculty of Science, Palacký University, 17. Listopadu 1192/12, CZ-771 46 Olomouc, Czech Republic; pavellasak@seznam.cz; 2 Institute of Molecular and Translation Medicine, Faculty of Medicine, Palacký University, Hněvotínská 5, CZ-779 00 Olomouc, Czech Republic; kamil.motyka@gmail.com; 3 Laboratory of Growth Regulators, Faculty of Science, Palacký University and Institute of Experimental Botany ASCR, Šlechtitelů 27, CZ-783 71 Olomouc, Czech Republic; vladimir.krystof@upol.cz

**Keywords:** phenanthridines, benzo[*c*]phenanthridines, radical cyclization, antiproliferative, antibacterial activity

## Abstract

In this study, we report the synthesis, antibacterial and anticancer evaluation of 38 novel phenanthridines that were designed as analogs of the benzo[*c*]phenanthridine alkaloids. The prepared phenanthridines differ from the benzo[*c*]phenanthridines in the absence of a benzene A-ring. All novel compounds were prepared from 6-bromo-2-hydroxy-3-methoxybenzaldehyde in several synthetic steps through reduction of Schiff bases and accomplished by radical cyclization. Twelve derivatives showed high antibacterial activity against *Bacillus* *subtilis*, *Micrococcus* *luteus* and/or *Mycobacterium* *vaccae* at single digit micromolar concentrations. Some compounds also displayed cytotoxicity against the K-562 and MCF-7 cancer cell lines at as low as single digit micromolar concentrations and were more potent than chelerythrine and sanguinarine. The active compounds caused cell-cycle arrest in cancer cells, increased levels of p53 protein and caused apoptosis-specific fragmentation of PARP-1. Biological activity was connected especially with the presence of the *N*-methyl quaternary nitrogen and 7-benzyloxy substitution (compounds **7i**, **7j**, **7k**, and **7l**) of phenanthridine.

## 1. Introduction

**Figure 1:**
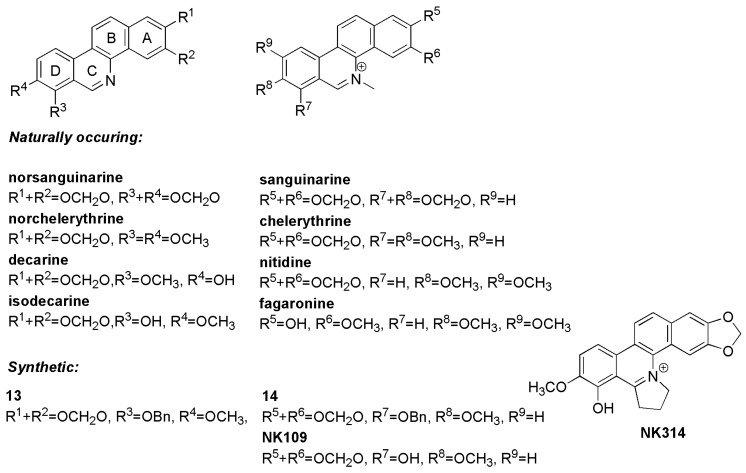
Structures of some benzo[*c*]phenanthridines.

Benzo[*c*]phenanthridine alkaloids contain a chrysene-skeleton-based heterocyclic core and are classified as isoquinoline alkaloids (Figure 1). They are distributed especially in the plant family *Papaveraceae* and display a broad spectrum of biological activities, including anti-inflammatory, antimicrobial, antifungal and antitumor effects [1,2,3,4,5,6,7]. Sanguinarine and chelerythrine are the best-known benzo[*c*]phenanthridine alkaloids, most frequently studied for their antitumor effects [8]. As flat polyaromatic compounds they directly interact with DNA [9]. However, cell cycle arrest and induction of cell death caused by these alkaloids probably occurs not only due to DNA damage alone, but as a combined result of targeting other cellular structures, including topoisomerases, tubulin and antiapoptotic protein Bcl-X_L_ [10,11]. Importantly, these alkaloids act at concentrations comparable to those of cytostatics used as anticancer drugs.

Benzo[*c*]phenanthridine alkaloids have been subjected to chemical modifications with the aim of understanding their structure-activity relationships and to improve their biological functions. Early studies indicated that the activity of benzo[*c*]phenanthridine alkaloids can be linked to the presence of their cationic quaternary nitrogen [12]. The quaternary iminium may undergo nucleophilic addition to biological amines or thiols, modify DNA and proteins and as a consequence induce cell death. In contrast, a recent report suggests that the iminium group is not essential for cytotoxicity in cancer cell lines [13]. Other systematic studies examined the correlation between the type of substituents at positions 2, 3, 7, 8 and 9 of their skeletons and antiproliferative activity [14] and revealed that cationic iminium alkaloids displayed stronger activity than their corresponding uncharged bases. The cytotoxic activity was further increased if these quaternary bases were 7,8-oxygenated [15].

These studies led to identification of NK109, the 7-*O*-demethylated synthetic analogue of chelerythrine, that was shown to have submicromolar dose growth-inhibitory activity against cancer cell lines, i.e., significantly lower than chelerythrine [16,17,18,19] (Figure 1). Further modification of NK109, in which ring C was fused to a pyrrolidine cycle, yielded NK314 with even stronger activities in several cancer models [20,21,22]. The molecular mechanism of action of these compounds has been often attributed to inhibition of topoisomerases, which are also targeted by the related compounds nitidine and fagaronine [23].

To our knowledge, there have not been many attempts to modify the heterocyclic skeleton of benzo[*c*]phenanthridines up until now in order to modify their biological activity. With the exception of benzo[*h*]quinolones, which are structurally related to the benzo[*c*]phenanthridines but lack the D-ring [24], and phenanthridines lacking the A-ring [25], most other reports describe the synthesis of various isomeric and aza-analogous structures such as benzo[*i*]phenanthridines and dibenzo[*c*,*h*]cinnolines [26], benzo[*c*]phenanthrolines [27], pyridophenanthrolines and azapyrimido-phenanthrolines [28] or compounds containing the 3,4-dihydroisoquinolin-2-ium scaffold [29,30]. 

**Figure 2:**
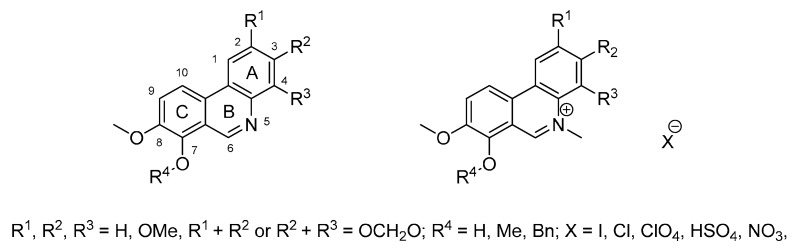
Desired phenanthridines for biological evaluation (for details see Table 1).

We therefore decided to prepare novel phenanthridines related to benzo[*c*]phenanthridines lacking the benzene A-ring (Figure 2), because previous work has suggested that phenanthridines may retain biological activity [25]. The series contains derivatives differing in the presence and position of hydroxy and methoxy groups, 2,3- or 3,4-methylenedioxy bridges and 7-benzyloxy groups, but all compounds contain an 8-methoxy group. Some derivatives have been prepared either as free bases or protonated salts. In some compounds, the heterocyclic nitrogen is available in a quaternary form with methyl substitution. Thus, such variability allowed us to build a preliminary structure-activity relationship for antibacterial and antiproliferative activity, and to compare the phenanthridines with known benzo[*c*]phenanthridines (Figure 1).

For the preparation of novel phenanthridine derivatives we adapted methodologies from benzo[*c*]phenanthridine chemistry. Many synthetic approaches to the benzo[*c*]phenanthridine nucleus have been reported, with most routes involving the construction of either the B or C ring in the final or semifinal stage. These synthetic methods are summarized in reviews [31,32]. Our synthesis of desired phenanthridine compounds is based on our previous experience with the synthesis of isodecarine [33] and metabolites of benzo[*c*]phenanthridines [34], where a radical cyclization leading to ring C closure was used to accomplish the construction of the benzo[*c*]phenanthridine heterocyclic system. By this method we prepared phenanthridine derivatives where the B ring is formed.

## 2. Results and Discussion

### 2.1. Chemistry

For the preparation of phenanthridines, selected anilines possessing substitution in positions 2,3 (**1b [35]**, **1d [36]**) and 3,4 (**1a**, **1c**) with methylenedioxy or dimethoxy groups were used for the reactions. Commercially available 6-bromo-2-hydroxy-3-methoxybenzaldehyde (**2a**) needed for introduction of the C-ring aromatic core was used as is or after modification of the hydroxyl group to obtain the methoxyaldehyde **2b** [25] or benzyloxyaldehyde **2c** [16]. 

Reductive amination of aldehydes **2a**–**2c** with selected anilines **1a**–**1d** by NaBH(OAc)_3_ in toluene proceeded quantitatively and afforded the corresponding novel secondary amines **4**. We have also verified an alternative two-step route leading to the same secondary amines through their isolated Schiff bases **3**. The preparation of Schiff bases **3a**, **3c**, and **3d** possessing unsubstituted hydroxyl groups proceeded smoothly providing products that readily precipitated from the reaction mixture with excellent nearly quantitative yields. Facile reduction of their iminium double bonds by NaBH_4_ in ethanol led to the corresponding secondary amines **4a**, **4c**, and **4d**. Surprisingly, the condensation leading to the formation of Schiff bases with substituted hydroxyl group (R^4^ = benzyl or methyl) under the same reaction conditions was not quantitative (conversions ranged from 50–75%) and subsequent isolation attempts by conventional methods were complicated. Therefore, we tried to influence the reaction equilibrium by increasing the ratio of aniline to aldehyde, prolonging the reaction time, performing the reactions in alternative solvents or by use of a microwave reactor, but unfortunately, without any satisfactory improvement. In summary, reductive amination seems to be a more suitable method for the preparation of these sorts of amines **4**.

To obtain compounds possessing a phenanthridine core, amines **4** needed to be cyclized. For this purpose, several synthetic approaches including palladium-catalyzed cross-coupling cyclization [37,38,39,40,41] or base-promoted homolytic aromatic substitution [42,43] were tested with amines **4a** and **4e**, unfortunately, the reactions failed or yields were very low. During these experiments debromination was the main reaction observed. Finally, radical cyclization by Bu_3_SnH using AIBN (azobisisobutyronitrile) as a radical initiator in toluene and subsequent aromatization by activated MnO_2_ [44] afforded the desired novel phenanthridine derivatives **5** in various yields. During all radical cyclization reactions we observed formation of debrominated starting amines **10** as side products in amounts of 10–20% (Scheme 2). Formation of these reduced compounds is in accordance with earlier results [33]. Further side products were detected when 3,4-disbstituted (R^1^ and R^2^) amines **4** were subjected to the radical cyclization conditions. Apart from reduced amines **10**, cyclized regioisomers **11** were also observed as minor products. In the case of substituted amines **4**, where R^4^ is benzyl or methyl the amount of **11** was around 5%, whereas the ratio of these regioisomers was much higher (up to 30%, based on HPLC analysis) for the unsubstituted hydroxylamines **4a**–**4d** (Scheme 2). 

Crystallization was utilized here as a useful method for the separation of most phenanthridines **5** from their reaction by-products. Unfortunately, a problem concerning the isolation of pure compounds was observed with hydroxyphenanthridines **5a**–**5d**. The byproducts formed had similar properties to the targeted molecules, with only silica gel column chromatography providing pure compound **5d**, but again with difficulties. For these reasons the remaining hydroxyphenanthridines **5a**–**5c** were obtained by catalytic hydrogenation of the corresponding benzyl derivatives **5i**–**5k**. This benzyl group deprotection proceeded under atmospheric pressure of hydrogen on 10% Pd/C. Under these conditions reduction of the iminium double bond can occur, so subsequent stirring of the reaction mixture in air or by addition of MnO_2_ was necessary for rearomatization. Alternatively, to avoid the need for this re-oxidation step benzyl group removal could also be achieved by acid hydrolysis with HCl followed by treatment with aqueous NH_3_ to obtain the free bases (Scheme 3).

**Scheme 1:**
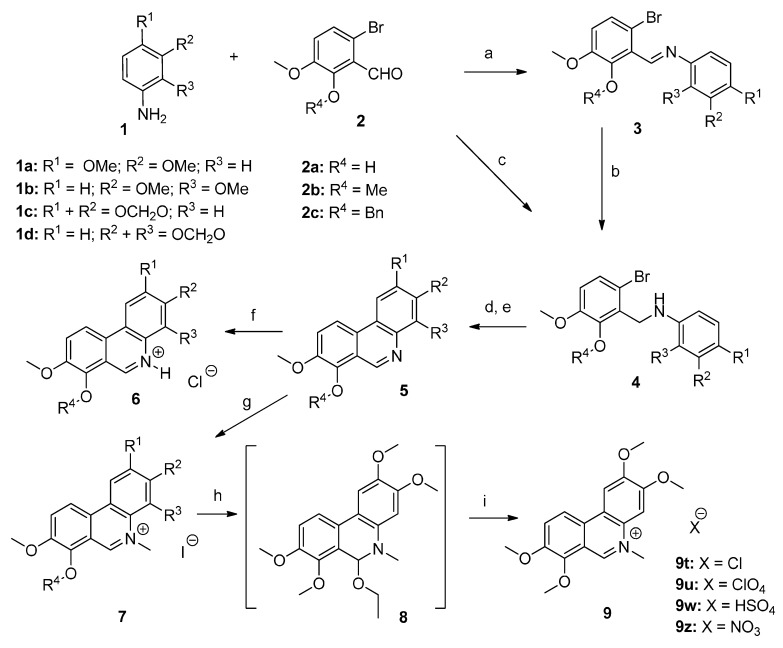
The synthetic route for the preparation of phenanthridine derivatives. *Reagents and conditions*: (a) EtOH, reflux, 1 h; (b) NaBH_4_, EtOH, r.t., 2 h; (c) NaBH(OAc)_3_, toluene, r.t., 1 h; (d) Bu_3_SnH, AIBN, toluene, 104–106 °C, 3–6 h; (e) activated MnO_2_, r.t., 18 h; (f) HCl, dioxane, r.t.; (g) MeI, ACN, r.t., 7 days; (h) NaOH, EtOH, r.t., 1 h; (i) HX, r.t., 10 min.

**Scheme 2:**
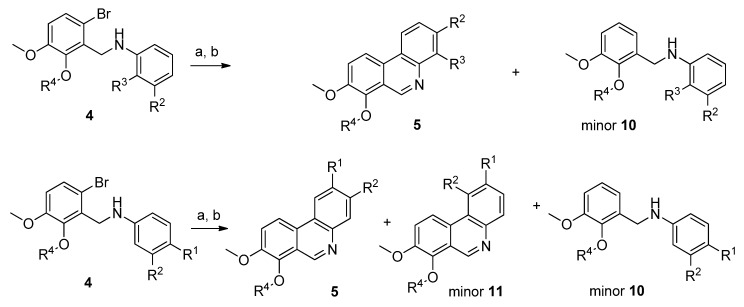
Side reaction products formed during radical cyclization of **4** to **5**. *Reagents and conditions*: (a) Bu_3_SnH, AIBN, toluene, 104–106 °C, 3–6 h; (b) activated MnO_2_, r.t., 18 h.

**Scheme 3:**
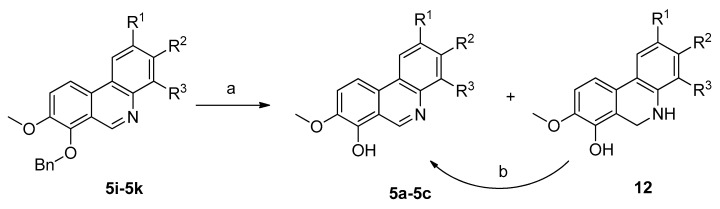
Convenient route for the preparation of hydroxy phenanthridines **5a**–**5c**. *Reagents and conditions*: (a) H_2_/10% Pd(C), atm. pressure, r.t. or HCl/EtOH, reflux 2.5 h, then aq. NH_3_; (b) air (O_2_) or MnO_2_; r.t.

Phenanthridine derivatives **5** were converted to their hydrochlorides **6** by addition of HCl into dioxane solutions of the free bases (Scheme 1). Surprisingly these compounds have very low solubility in water, and even in other polar solvents (DMSO, EtOH, DMF) which hindered biological testing.

*N*-methylation of phenanthridines **5** was carried out with methyl iodide in acetonitrile under mild conditions to give *N*-methylphenanthridinium iodides **7**. These conditions enabled the selective methylation, even of the phenanthridines possessing a free hydroxyl group. Similarly to the hydrochlorides **6**, the prepared quaternary iodides **7** were also surprisingly very poorly soluble in water. To verify the possibility if the anion exchange can influence the solubility of these compounds, selected quaternary iodides were transformed in a NaOH/EtOH solution into their unisolated colorless pseudobases **8**. Different salts (chloride, hydrogensulfate, perchlorate, and nitrate) were obtained by addition of large excess of corresponding acids to provide the anion modified *N*-methylphenanthridine precipitates. It was found that anion exchange did not improve solubility of these mentioned compounds significantly. For illustration, we demonstrate experimentally the preparation of various salts of compound **9**, which were confirmed by elemental analysis. 

### 2.2. Biological Assays

#### 2.2.1. Antibacterial Activity

Prepared derivatives were screened for antibacterial activity against representative Gram-positive bacteria (*Bacillus. subtilis,* ATCC 6633; *Micrococcus luteus,* ATCC 10240; *Mycobacterium vaccae*, DSM 43514; *Staphylococcus aureus*, CCM 2524) and Gram-negative bacteria (*Pseudomonas aeruginosa*, CCM 3955; *Escherichia coli*, CCM 3954) using agar diffusion assays [45]. Derivatives with zones of inhibition ≥20 mm were subjected to a further assay [46] that determines their minimum inhibitory concentrations (MIC). From the measured data (Table 2) it is possible to make a few general conclusions regarding their structure-activity relationships:

(1) Antibacterial activity was observed only for derivatives containing a phenanthridine skeleton. No tested strain was susceptible to representative intermediates **3** and **4**.
(2) Derivatives with benzyl substituent as R^4^ (**5k**, **5l**, **7i**, **7j**, **7k**, and **7l**) showed high antibacterial activity against *B. subtilis*, *M. luteus* and/or *M. vaccae* with MIC in single digit micromolar values.
(3) Compounds with a charged nitrogen bearing methyl group (**7i**, **7j**, **7k** and **7l**) demonstrated high activity as well. To summarize, the most active compounds have a similar structural motif—a phenanthridine skeleton with a charged *N*-methyl nitrogen and a benzyl group as a R^4^ substituent.

These points are noteworthy for potential structural exploitation in further antibacterial agent development. To compare the antibacterial activity results for well-known natural (chelerythrine, sanguinarine, isodecarine, norchelerythrine) or synthetic compounds (NK-109, **13**, **14**) with similar structure motifs are presented in Table 2 as well. From the measured results it is evident that some prepared derivatives provided similar or even better activity than these compounds (MIC in micromolar or submicromolar values). All newly reported compounds were less active than a previously developed analogue 14, with 7j performing better than that reference compound only against *M. luteus*. No compound displayed any relevant activity against Gram-negative bacteria. If the structures are compared the importance of the presence of a charged *N*-methyl nitrogen in the molecule is confirmed.

#### 2.2.2. Anticancer Activity In Vitro

Benzo[*c*]phenanthridine alkaloids are known to display antiproliferative and anticancer activities [8,18]. Due to their structural analogy, the preliminary in vitro anticancer activity of the newly prepared compounds was evaluated on two established cancer cell lines, MCF-7 (breast carcinoma) and K-562 (chronic myelogeneous leukemia). The resulting data are presented in Table 3 and show that several of the new compounds have significant activity in both cell lines, with single digit micromolar EC_50_ values. 

Derivative **7j** reached submicromolar values in K-562 cells and was even more potent than chelerythrine and sanguinarine. The most active compounds bear 7-benzyloxy substitution and either 2,3-dimethoxy or an isosteric 2,3-methylenedioxy bridge and are either *N*5-methylated (**7j**, **7k**) or contain an unsubstituted *N*5 (**5k**). Compounds without a benzyl moiety or with 3,4-dimethoxy/3,4-methylenedioxy substitution were less potent. Interestingly, removal of the methyl from *N*5 of **7j**, that also uncharged the nitrogen, decreased potency (**5j**). In contrast, presence of the quaternary nitrogen (methylated) significantly reduced the activity of several compounds (compare especially pairs **5f** and **7f**, **5g** and **7g**, **5h** or **7h**). According to previous studies, the activity of benzo[*c*]phenanthridine alkaloids can be explained by the presence of a cationic quaternary nitrogen [12,15]. These conclusions however probably cannot be applied to phenanthridines, because many of these compounds with a quaternary nitrogen have poor activity and one of the most potent compounds (**5k**) does not contain a quaternary nitrogen at all. 

With the aim of directly comparing the activity of phenanthridines with the corresponding benzo[*c*]phenanthridines, we prepared also four variously substituted benzo[*c*]phenanthridines—**13 [16,33]**, **14** [33], isodecarine [33] and norchelerythrine (Figure 1). In line with the abovementioned findings for phenanthridines, the presence of the benzyloxy functionality at position 7 correlated with in vitro anticancer activity for compounds **13** and **14**. However, previous studies indicated that the activity of benzo[*c*]phenanthridine alkaloids can be explained by the presence of a cationic quaternary nitrogen atom [12]. We also observed lack of cellular activity with isodecarine and norchelerythrine, which were prepared as demethylated derivatives of the very potent NK-109 and chelerythrine, respectively. Compound **14**, killing both cancer cell lines with EC_50_ values around 1 µM, contains not only a benzyloxy functionality, but also a quaternary nitrogen that could be essential for its activity. We cannot rule out the possibility that this cation is even more important for the activity. 

#### 2.2.3. Mechanism of Cellular Activity

Anticancer activity of benzo[*c*]phenanthridine alkaloids relates at least partly to their ability to inhibit topoisomerase I or II. For example, nitidine and fagaronine inhibit the topoisomerase I—mediated DNA relaxation [23]. 

**Figure 3:**
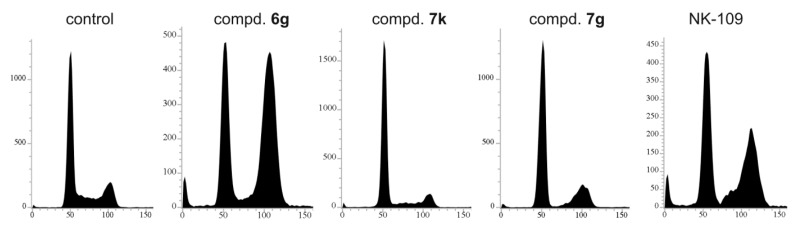
Cell cycle effects of compounds **7k**, **7g**, **6g** and NK-109 in MCF-7 cells treated for 24 h with doses corresponding to 3 × EC_50_ values (60, 5.4, 33 and 13.8 µM, respectively). Cells were harvested and then flow cytometric analysis of DNA stained by propidium iodide (10,000 counts) was performed as described in the Materials and Methods section.

Their synthetic derivatives NK109 and NK314 exerted their cytotoxic activity through inhibition of topoisomerase II, followed by DNA breaks [19,22]. DNA damage usually results in a cell cycle arrest and eventually leads to apoptosis. We therefore treated MCF-7 cells with several compounds and NK109 as a control. Interestingly, these compounds influenced the cell cycle profile in different manners (Figure 3). The most potent **7k** reduced S and G2/M phases, **7g** reduced S phase and caused slight accumulation in G2/M, whereas the only weakly cytotoxic **6g** resulted in strong G2/M arrest. NK109 used as a control also arrested cells in G2/M. These differences suggest that the compounds may have different molecular targets.

**Figure 4:**
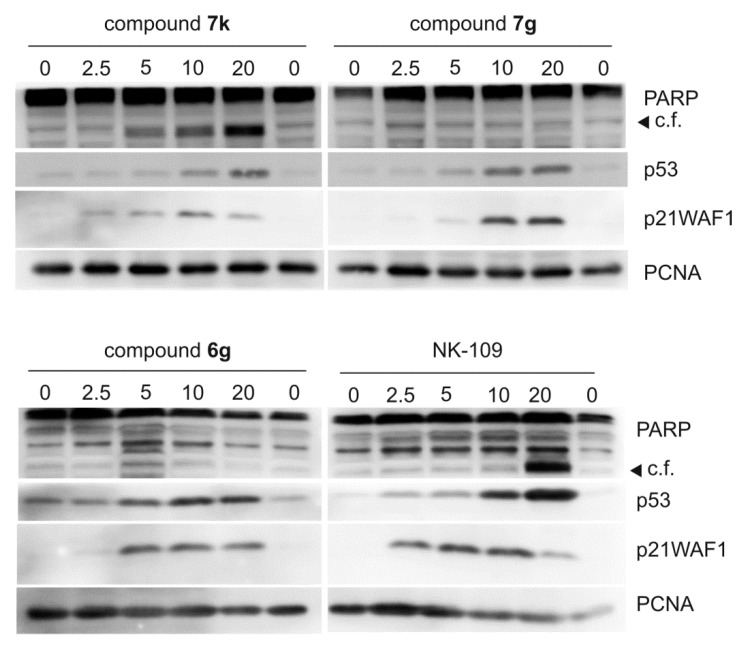
Dose-dependent effect of compounds **7k**, **7g**, **6g** and **NK-109** on p53 and its activity in MCF-7 cells. The cells were treated for 24 h with indicated doses of compounds (given in µM) and then specific proteins were analyzed by immunoblotting as described in the Materials and Methods section. C.f., 89 kDa cleavage fragment of PARP.

DNA damage induces various responses, including stabilization and activation of tumor suppressor protein p53 [47]. In the following experiments, we analyzed levels and activities of tumor suppressor protein p53, which is typically activated in cells upon topoisomerase inhibition. We performed immunoblotting of proteins extracted from MCF-7 cells treated for 24 h with compounds **6g**, **7k** and **7g**. Levels of p53 and its typical downstream target p21^waf1^ were both increased (Figure 4). In addition, fragmentation of PARP producing a 89 kDa band was observed in cells treated with **7k** and NK109, indicating ongoing apoptosis. This fragmentation was dose-dependent for compound **7k**. In contrast, neither **6g** nor **7g** triggered PARP fragmentation, which is in line with their weaker cytotoxicities.

## 3. Materials and Methods

### 3.1. Chemistry

#### 3.1.1. General Information

All commercially available reagents were used without further purification and purchased from standard chemical suppliers. Reactions were monitored by LC/MS analyses on a UHPLC-MS system (Thermo Scientific, Waltham, MA, USA) consisting of a UHPLC chromatograph equipped with a photodiode array detector and a triple quadrupole mass spectrometer using a C18 column at 30 °C and flow rate of 800 μL/min. ^1^H and ^13^C-NMR spectra were measured on an ECA 400II (^1^H: 399.78 MHz, ^13^C: 100.53 MHz) NMR spectrometer (JEOL Resonance, Tokyo, Japan). Samples were dissolved and subsequently measured in DMSO-*d*_6_ or CDCl_3_. Chemical shifts (δ) are reported in ppm and referenced to the middle of the solvent signal (DMSO-*d*_6_: 2.50 ppm, 39.51 ppm; CDCl_3_: 7.27 ppm, 77.00 ppm. Data are reported as follows: chemical shift (multiplicity [singlet (s), doublet (d), doublet of doublet (dd), triplet (t), quartet (q), multiplet (m), broad resonance (br)], coupling constants [Hz], integration). All the NMR spectra were acquired at ambient temperature. All recorded ^1^H and ^13^C-NMR spectra are available as Supplementary material. High resolution mass spectra (HRMS) measurements were performed on an Orbitrap mass analyzer (Thermo Scientific, Waltham, MA, USA) equipped with Heated Electrospray Ionization (HESI). The spectrometer was tuned to obtain a maximum response for *m*/*z* 70–700. Elemental analyses were performed on an EA 1108 Elemental Analyser (Fisons Instruments, Thermo Scientific, Waltham, MA, USA). Thin layer chromatography (TLC) were performed on pre-coated silica gel 60 F254 plates (Merck, Prague, Czech Republic) and visualized by exposure to UV light (254 or 366 nm). Melting points were measured on a Boetius stage apparatus (WEB Analytik, Dresden, Germany) and are uncorrected.

#### 3.1.2. Synthesis of Compounds **3**–**9**

*3-Bromo-2-{[(3,4-dimethoxyphenyl)imino]methyl}-6-methoxyphenol* (**3a**). To a solution of aniline **1a** (410 mg; 2.67 mmol) in anhydrous EtOH (15 mL) was added benzaldehyde **2a** (600 mg; 2.60 mmol). The reaction mixture was stirred at reflux for 1 h and then slowly cooled to 0–5 °C. Resultant precipitate was filtered off, washed with ice-cold EtOH (2 mL) and dried. Compound **3a** (921 mg, 97%) was obtained as an orange microcrystalline solid. Mp. = 157–159 °C; ^1^H-NMR (400 MHz, CDCl_3_) δ = 15.27 (s, 1H), 9.05 (s, 1H), 7.02 (d, *J* = 8.8 Hz, 1H), 6.98–6.89 (m, 3H), 6.78 (d, *J* = 8.8 Hz, 1H), 3.94 (s, 3H), 3.92 (s, 3H), 3.90 (s, 3H); ^13^C-NMR (101 MHz, CDCl_3_) δ = 160.4, 154.6, 149.6, 148.8, 148.7, 140.0, 121.8, 116.4, 115.7, 114.7, 113.1, 111.4, 105.1, 56.2, 56.1, 56.0; HRMS (HESI, *m*/*z*): [M + H]^+^ calcd. for C_16_H_17_BrNO_4_, 366.0341; found 366.0335.

*2-[(1,3-Benzodioxole-5-ylimino)methyl]-3-bromo-6-methoxyphenol* (**3c**). Schiff base **3c** was obtained in a similar manner to compound **3a** using benzaldehyde **2a** (750 mg; 3.25 mmol), aniline **1c** (445 mg; 3.25 mmol) and anhydrous EtOH (20 mL). Compound **3c** (1.09 g; 92%) was obtained as a dark red microcrystalline solid. Mp. = 176–178 °C; ^1^H-NMR (400 MHz, CDCl_3_) δ = 15.07 (br. s, 1H), 9.02 (s, 1H), 7.03 (d, *J* = 8.7 Hz, 1H), 6.92–6.89 (m, 1H), 6.88–6.85 (m 2H), 6.78 (d, *J* = 8.7 Hz, 1H), 6.03 (s, 2H), 3.91 (s, 3H); ^13^C-NMR (101 MHz, CDCl_3_) δ = 160.6, 154.4, 148.8, 148.7, 147.4, 141.4, 122.0, 116.5, 116.0, 115.9, 114.9, 108.7, 101.8, 101.5, 56.3; HRMS (HESI, *m*/*z*): [M + H]^+^ calcd. for C_15_H_13_BrNO_4_, 350.0028; found 350.0023.

*2-[(1,3-Benzodioxole-4-ylimino)methyl]-3-bromo-6-methoxyphenol* (**3d**). Schiff base **3d** was obtained in a similar manner to compound **3a** using benzaldehyde **2a** (572 mg; 2.48 mmol), aniline **1d** (350 mg; 2.55 mmol) and anhydrous EtOH (15 mL). Compound **3d** (737 mg; 85%) was obtained as a pale orange microcrystalline solid. Mp. = 195–196 °C; ^1^H-NMR (400 MHz, CDCl_3_) δ = 15.18 (s, 1H), 9.40 (s, 1H), 7.05 (d, *J* = 8.6 Hz, 1H), 6.96–6.88 (m, 2H), 6.82–6.77 (m, 2H), 6.09 (s, 2H), 3.92 (s, 3H); ^13^C-NMR (101 MHz, CDCl_3_) δ = 164.3, 154.7, 148.9, 148.7, 140.3, 129.3, 122.2, 121.9, 116.7, 116.3, 116.0, 115.0, 107.7, 101.7, 56.2; HRMS (HESI, *m*/*z*): [M + H]^+^ calcd. for C_15_H_13_BrNO_4_, 350.0028; found 350.0022.

*3-Bromo-2-{[(3,4-dimethoxyphenyl)amino]methyl}-6-methoxyphenol* (**4a**). To a suspension of the Schiff base **3a** (921 mg, 2.51 mmol) in anhydrous EtOH (25 mL) was added NaBH_4_ (95 mg; 2.51 mmol). After 2 h of stirring at room temperature the reaction mixture was neutralized by adding an aqueous solution of 10% AcOH (1 mL). The suspension dissolved into solution, which was concentrated under reduced pressure. The residue was extracted with a mixture of EtOAc (20 mL) and water (20 mL). The aqueous phase was washed with EtOAc (20 mL). The combined EtOAc phases were dried over Na_2_SO_4_, filtered and concentrated under reduced pressure to provide a yellow crude oil (737 mg, 80%). TLC R_f_ = 0.3 (hexane/EtOAc 10:3 *v*/*v*); ^1^H-NMR (400 MHz, CDCl_3_) δ = 7.05 (d, *J* = 9.3 Hz, 1H), 6.74 (d, *J* = 8.3 Hz, 1H), 6.66 (d, *J* = 8.3 Hz, 1H), 6.47 (d, *J* = 2.1 Hz, 1H), 6.37 (dd, *J* = 2.1, 8.3, Hz, 1H), 5.89 (brs, 2H), 4.53 (s, 2H), 3.85 (s, 3H), 3.83 (s, 3H), 3.80 (s, 3H); ^13^C-NMR (101 MHz, CDCl_3_) δ = 149.7, 146.6, 146.2, 142.7, 141.7, 123.4, 123.2, 115.1, 112.7, 111.2, 106.1, 100.5, 56.5, 56.1, 55.7, 45.8; HRMS (HESI, *m*/*z*): [M + H]^+^ calcd. for C_16_H_19_BrNO_4_, 368.0492; found 368.0498.

*2-[(1,3-Benzodioxole-5-ylamino)methyl]-3-bromo-6-methoxyphenol* (**4c**). The secondary amine **4c** was obtained in a similar manner as compound **4a** using Schiff base **3c** (1.0 g; 2.9 mmol), NaBH_4_ (109 mg; 2.9 mmol) and anhydrous EtOH (25 mL). The resulting crude product was a pale yellow oil (1.0 g, 99%). A sample for analysis was prepared by crystallization from toluene (100 mg/3 mL) and standing this solution at −20 °C. Mp. = 95–98 °C as a colorless crystalline compound; TLC R_f_ = 0.3 (hexane/EtOAc 10:3 *v*/*v*); ^1^H-NMR (400 MHz, DMSO-*d*_6_) δ = 9.34 (s, 1H), 7.02 (d, *J* = 8.7 Hz, 1H), 6.86 (d, *J* = 8.7 Hz, 1H), 6.64 (d, *J* = 8.2 Hz, 1H), 6.42 (d, *J* = 1.8 Hz, 1H), 6.12 (dd, *J* = 8.3, 2.3 Hz, 1H), 5.82 (s, 2H), 5.21 (t, *J* = 5.2 Hz, 1H), 4.19 (d, *J* = 5.2 Hz, 2H), 3.79 (s, 3H); ^13^C-NMR (101 MHz, DMSO-*d*_6_) δ = 147.6, 147.1, 146.2, 144.6, 138.1, 124.7, 122.2, 115.6, 112.2, 108.3, 103.4, 99.9, 95.4, 56.0, 42.9; HRMS (HESI, *m*/*z*): [M + H]^+^ calcd. for C_15_H_15_BrNO_4_, 352.0179; found 352.0182.

*2-[(1,3-Benzodioxole-4-ylamino)methyl]-3-bromo-6-methoxyphenol* (**4d**). The secondary amine **4d** was obtained in a similar manner as compound **4a** using Schiff base **3d** (500 mg; 1.43 mmol), NaBH_4_ (54 mg; 1.43 mmol) and anhydrous EtOH (13 mL). The resulting crude product was a pale yellow oil (500 mg, 99%). A sample for analysis was prepared by crystallization from toluene (100 mg/3 mL) and standing this solution at −20 °C. Mp. = 89–90 °C as a colorless crystalline compound; TLC R_f_ = 0.3 (hexane/EtOAc 10:3 *v*/*v*); ^1^H-NMR (400 MHz, CDCl_3_) δ = 7.07 (d, *J* = 8.7 Hz, 1H), 6.75 (t, *J* = 8.0 Hz, 1H), 6.68 (d, *J* = 8.7 Hz, 1H), 6.60 (d, *J* = 8.2 Hz, 1H), 6.36 (d, *J* = 7.8 Hz, 1H), 5.90 (s, 2H), 4.59 (s, 2H), 3.87 (s, 3H); ^13^C-NMR (101 MHz, CDCl_3_) δ = 147.2, 146.2, 145.6, 134.6, 132.2, 123.9, 123.3, 122.2, 115.7, 111.1, 107.8, 100.6, 99.8, 56.1, 43.7; HRMS (HESI, *m*/*z*): [M + H]^+^ calcd. for C_15_H_15_BrNO_4_, 352.0179; found 352.0183.

*N-(6-Bromo-2,3-dimethoxybenzyl)-3,4-dimethoxyaniline* (**4e**). To a solution of bromobenzaldehyde **2b** (1.0 g; 4.08 mmol) and aniline **1a** (644 mg; 4.20 mmol) in anhydrous toluene (50 mL) was added NaBH(OAc)_3_ (2.6 g; 12.2 mmol). The resulting suspension was stirred at room temperature for 1 h, and then washed with water (2 × 35 mL) and brine (1 × 40 mL). The aqueous phase was extracted with toluene (1 × 40 mL). The combined organic extracts were dried over Na_2_SO_4_, filtered and concentrated under reduced pressure. To induce crystallization of the product, MeOH (8 mL) was added to the residue. The resulting solid was filtered off, washed with cold MeOH (1 mL) and dried. Compound **4e** was obtained as a slightly pink crystalline solid (1.19 g; 76%). Mp. = 79–80 °C; TLC R_f_ = 0.2 (hexane/EtOAc 10:3 *v*/*v*); ^1^H-NMR (400 MHz, CDCl_3_) δ = 7.26 (d, *J* = 8.8 Hz, 1H), 6.79–6.72 (m, 2H), 6.43 (d, *J* = 2.6 Hz, 1H), 6.33 (dd, *J* = 8.5, 2.6 Hz, 1H), 4.42 (s, 2H), 4.02 (br. s, 1H), 3.87 (s, 3H), 3.86 (s, 3H), 3.85 (s, 3H), 3.80 (s, 3H); ^13^C-NMR (101 MHz, CDCl_3_) δ = 152.3, 149.8, 148.7, 142.7, 141.7, 132.6, 128.0, 115.2, 113.0, 112.9, 104.6, 99.5, 61.5, 56.6, 55.9, 55.7, 43.9; HRMS (HESI, *m*/*z*): [M + H]^+^ calcd. for C_17_H_21_BrNO_4_, 382.0654; found 382.0648.

*N-(6-Bromo-2,3-dimethoxybenzyl)-2,3-dimethoxyaniline* (**4f**). The secondary amine **4f** was prepared in a similar manner as the amine **4e** using bromobenzaldehyde **2b** (400 mg; 1.63 mmol), aniline **1b** (258 mg; 1.68 mmol) and NaBH(OAc)_3_ (1.04 g; 4.9 mmol) in anhydrous toluene (20 mL). The residue was crystallized from EtOH/water to provide compound **4f** (212 mg; 34%) as a colorless microcrystalline solid. Mp. = 52–53 °C; TLC R_f_ = 0.5 (hexane/EtOAc 10:3 *v*/*v*); ^1^H-NMR (400 MHz, DMSO-*d*_6_) δ = 7.34 (d, *J* = 8.8 Hz, 1H), 6.98 (d, *J* = 8.8 Hz, 1H), 6.85 (t, *J* = 8.2 Hz, 1H), 6.52 (dd, *J* = 8.2, 1.2 Hz, 1H), 6.33 (dd, *J* = 8.2, 1.2 Hz, 1H), 4.85 (t, *J* = 6.2 Hz, 1H), 4.33 (d, *J* = 6.2 Hz, 2H), 3.80 (s, 3H), 3.77 (s, 3H), 3.72 (s, 3H), 3.60 (s, 3H); ^13^C-NMR (101 MHz, DMSO-*d*_6_) δ = 152.1, 148.4, 141.6, 135.0, 131.9, 127.9, 124.2, 119.5, 114.4, 113.8, 104.7, 101.6, 61.2, 59.5, 55.9, 55.5, 42.2; HRMS (HESI *m*/*z*): [M + H]^+^ calcd. for C_17_H_21_BrNO_4_, 382.0654; found 382.0648. 

*N-(6-Bromo-2,3-dimethoxybenzyl)-1,3-benzodioxole-5-amine* (**4g**). The secondary amine **4g** was prepared in a similar manner as the amine **4e** using bromobenzaldehyde **2b** (400 mg; 1.63 mmol), aniline **1c** (231 mg; 1.68 mmol) and NaBH(OAc)_3_ (1.04 g; 4.9 mmol) in anhydrous toluene (20 mL). The residue was crystallized from EtOH/water to provide compound **4g** (275 mg; 46%) as a pale yellow microcrystalline solid. Mp. = 74–75 °C; TLC R_f_ = 0.4 (hexane/EtOAc 10:3 *v*/*v*); ^1^H-NMR (400 MHz, DMSO-*d*_6_) δ = 7.34 (d, *J* = 8.8 Hz, 1H), 7.00 (d, *J* = 8.8 Hz, 1H), 6.65 (d, *J* = 8.3 Hz, 1H), 6.41 (d, *J* = 2.3 Hz, 1H), 6.12 (dd, *J* = 8.3, 2.3 Hz, 1H), 5.82 (s, 2H), 5.26 (t, *J* = 5.3 Hz, 1H), 4.16 (d, *J* = 5.2 Hz, 2H), 3.81 (s, 3H), 3.75 (s, 3H); ^13^C-NMR (101 MHz, DMSO-*d*_6_) δ = 152.1, 148.5, 147.7, 144.6, 138.1, 132.0, 127.7, 115.0, 113.7, 108.4, 103.2, 99.9, 95.2, 61.1, 55.9, 43.0; HRMS (HESI *m*/*z*): [M + H]^+^ calcd. for C_16_H_17_BrNO_4_, 366.0341; found 366.0337. 

*N-(6-Bromo-2,3-dimethoxybenzyl)-1,3-benzodioxole-4-amine* (**4h**). The secondary amine **4h** was prepared in a similar manner as the amine **4e** using bromobenzaldehyde **2b** (253 mg; 1.03 mmol), aniline **1d** (142 mg; 1.04 mmol) and NaBH(OAc)_3_ (660 mg; 3.1 mmol) in anhydrous toluene (12 mL). The residue was crystallized from MeOH to provide compound **4h** (302 mg; 80%) as a colorless microcrystalline solid. Mp. = 90–92 °C; TLC R_f_ = 0.3 (hexane/EtOAc 10:3 *v*/*v*); ^1^H-NMR (400 MHz, DMSO-*d*_6_) δ = 7.33 (d, *J* = 8.7 Hz, 1H), 6.98 (d, *J* = 9.2 Hz, 1H), 6.65 (t, *J* = 7.8 Hz, 1H), 6.46 (d, *J* = 8.2 Hz, 1H), 6.27 (dd, *J* = 7.8, 0.9 Hz, 1H), 5.90 (s, 2H), 4.82 (t, *J* = 6.0 Hz, 1H), 4.40 (d, *J* = 6.0 Hz, 2H), 3.80 (s, 3H), 3.77 (s, 3H); ^13^C-NMR (101 MHz, DMSO-*d*_6_) δ = 152.1, 148.4, 147.0, 133.2, 132.7, 131.8, 127.7, 122.1, 114.6, 113.7, 107.2. 100.0, 98.3, 61.0, 55.9, 42.6; HRMS (HESI *m*/*z*): [M + H]^+^ calcd. for C_16_H_17_BrNO_4_, 366.0341; found 366.0337.

*N-[2-(Benzyloxy)-6-bromo-3-methoxybenzyl]-3,4-dimethoxyaniline* (**4i**). The secondary amine **4i** was prepared in a similar manner as amine **4e** using bromobenzaldehyde **2c** (1.0 g; 3.11 mmol), aniline **1a** (491 mg; 3.21 mmol) and NaBH(OAc)_3_ (1.98 g; 9.4 mmol) in anhydrous toluene (50 mL). The residue was crystallized from MeOH to provide compound **4i** (1.16 g; 81%) as a light pink microcrystalline solid. Mp.= 79–81 °C; TLC R_f_ = 0.3 (hexane/EtOAc 10:3 *v*/*v*); ^1^H-NMR (400 MHz, DMSO-*d*_6_) δ = 7.43–7.29 (m, 6H), 7.03 (d, *J* = 8.8 Hz, 1H), 6.69 (d, *J* = 8.8 Hz, 1H), 6.39 (d, *J* = 8.6 Hz, 1H), 6.15 (dd, *J* = 8.6, 2.6 Hz, 1H), 5.10 (t, *J* = 5.6 Hz, 1H), 4.99 (s, 2H), 4.17 (d, *J* = 5.4 Hz, 2H), 3.86 (s, 3H), 3.61 (s, 6H); ^13^C-NMR (101 MHz, DMSO-*d*_6_) δ = 152.2, 149.8, 147.1, 143.7, 140.3, 137.1, 132.4, 128.4, 128.3, 128.1, 127.9, 115.0, 114.4, 113.6, 103.0, 98.7, 75.1, 56.6, 56.0, 55.1, 42.9; HRMS (HESI *m*/*z*): [M + H]^+^ calcd. for C_23_H_25_BrNO_4_, 458.0967; found 458.0961. 

*N-[2-(Benzyloxy)-6-bromo-3-methoxybenzyl]-2,3-dimethoxyaniline* (**4j**). The secondary amine **4j** was prepared in a similar manner as the amine **4e** using bromobenzaldehyde **2c** (900 mg; 2.80 mmol), aniline **1b** (442 mg; 2.89 mmol) and NaBH(OAc)_3_ (1.78 g; 8.4 mmol) in anhydrous toluene (45 mL). The residue was crystallized from EtOH to provide compound **4j** (758 mg; 75%) as a pale yellow microcrystalline solid. Mp. = 86–87 °C; TLC R_f_ = 0.5 (hexane/EtOAc 10/3 *v*/*v*); ^1^H-NMR (400 MHz, DMSO-*d*_6_) δ = 7.46–7.30 (m, 6H), 7.02 (d, *J* = 8.8 Hz, 1H), 6.81 (t, *J* = 8.2 Hz, 1H), 6.46 (d, *J* = 8.0 Hz, 1H), 6.32 (d, *J* = 8.3 Hz, 1H), 5.01 (s, 2H), 4.85 (t, *J* = 6.2 Hz, 1H), 4.27 (d, *J* = 4.9 Hz, 2H), 3.85 (s, 3H), 3.71 (s, 3H), 3.55 (s, 3H); ^13^C-NMR (101 MHz, DMSO-*d*_6_) δ = 152.2, 152.1, 147.0, 141.5, 137.1, 135.1, 132.2, 128.3, 128.2, 128.1, 128.0, 124.1, 114.4, 113.7, 104.6, 101.6, 74.9, 59.4, 56.0, 55.5, 42.4; HRMS (HESI *m*/*z*): [M + H]^+^ calcd. for C_23_H_25_BrNO_4_, 458.0967; found 458.0961.

*N-[2-(Benzyloxy)-6-bromo-3-methoxybenzyl]-1,3-benzodioxole-5-amine* (**4k**). The secondary amine **4k** was prepared in a similar manner as the amine **4e** using bromobenzaldehyde **2c** (600 mg; 1.87 mmol), aniline **1c** (263 mg; 1.93 mmol) and NaBH(OAc)_3_ (1.19 g; 5.6 mmol) in anhydrous toluene (33 mL). The residue was crystallized from MeOH/acetone/water mixture to provide compound **4k** (791 mg; 96%) as a pale brown microcrystalline solid. Mp. = 77–80 °C; TLC R_f_ = 0.5 (hexane/EtOAc 10:3 *v*/*v*); ^1^H-NMR (400 MHz, DMSO-*d*_6_) δ = 7.41–7.27 (m, 6H), 7.04 (d, *J* = 9.1 Hz, 1H), 6.64 (d, *J* = 8.3 Hz, 1H), 6.38 (d, *J* = 2.3 Hz, 1H), 6.08 (dd, *J* = 8.3, 2.3 Hz, 1H), 5.82 (s, 2H), 5.23 (t, *J* = 5.2 Hz, 1H), 4.98 (s, 2H), 4.12 (d, *J* = 5.4 Hz, 2H), 3.86 (s, 3H); ^13^C-NMR (101 MHz, DMSO-*d*_6_) δ = 152.2, 147.7, 147.2, 144.6, 138.1, 137.1, 132.2, 128.3, 128.3, 128.1, 127.9, 115.1, 113.7, 108.4, 103.2, 99.9, 95.3, 75.0, 56.1, 43.2; HRMS (HESI *m*/*z*): [M + H]^+^ calcd. for C_22_H_21_BrNO_4_, 442.0654; found 442.0648. 

*N-[2-(Benzyloxy)-6-bromo-3-methoxybenzyl]-1,3-benzodioxole-4-amine* (**4l**). The secondary amine **4l** was prepared in a similar manner as the amine **4e** using bromobenzaldehyde **2c** (1.0 g; 3.11 mmol), aniline **1d** (439 mg; 3.20 mmol) and NaBH(OAc)_3_ (1.98 g; 9.3 mmol) in anhydrous toluene (45 mL). The residue was crystallized from EtOH to provide compound **4l** (1.24 g; 90%) as a white microcrystalline solid. Mp. = 94–96 °C; TLC R_f_ = 0.4 (toluene); ^1^H-NMR (400 MHz, DMSO-*d*_6_) δ = 7.41–7.31 (m, 6H), 7.03 (d, *J* = 9.6 Hz, 1H), 6.62 (t, *J* = 8.3 Hz, 1H), 6.41 (d, *J* = 7.6 Hz, 1H), 6.26 (d, *J* = 7.6 Hz, 1H), 5.86 (s, 2H), 5.01 (s, 2H), 4.75 (t, *J* = 6.0 Hz, 1H), 4.33 (d, *J* = 6.0 Hz, 2H), 3.85 (s, 3H); ^13^C-NMR (101 MHz, DMSO-*d*_6_) δ = 152.2, 147.1, 147.0, 137.1, 133.2, 132.7, 132.1, 128.3 (2×), 128.1, 127.9, 122.1, 114.7, 113.8, 107.3, 100.0, 98.4, 74.9, 56.1, 42.7; HRMS (HESI *m*/*z*): [M + H]^+^ calcd. for C_22_H_21_BrNO_4_, 442.0654; found 442.0652.

*2,3,8-Trimethoxyphenanthridin-7-ol* (**5a**). To a suspension of benzylated phenanthridine **5i** (52 mg; 0.14 mmol) in MeOH (8 mL) was added 10% Pd/C (5 mg) and the mixture was vigorously stirred under atmospheric pressure of hydrogen for 4 h. It was then filtered and concentrated under reduced pressure to afford compound **5a** (28 mg; 71%) as an orange microcrystalline solid. Mp. = 212–215 °C; TLC R_f_ = 0.2 (CHCl_3_/MeOH 40/2 *v*/*v*); ^1^H-NMR (400 MHz, DMSO-*d*_6_) δ = 9.88 (br. s., 1H), 9.40 (s, 1H), 8.18 (d, *J* = 9.1 Hz, 1H), 7.98 (s, 1H), 7.64 (d, *J* = 8.8 Hz, 1H), 7.47 (s, 1H), 4.00 (s, 3H), 3.95 (s, 3H), 3.93 (s, 3H); ^13^C-NMR (101 MHz, DMSO-*d*_6_) δ = 149.9, 149.3, 145.5, 143.7, 142.7, 138.7, 126.4, 118.0, 117.7, 116.4, 112.8, 109.7, 102.7, 56.9, 55.9, 55.5; HRMS (HESI *m*/*z*): [M + H]^+^ calcd. for C_16_H_16_NO_4_ 286.1079; found 286.1074.

*3,4,8-Trimethoxyphenanthridin-7-ol* (**5b**). Compound **5b** was prepared in a similar manner as the base **5a** using benzylated phenanthridine **5j** (20 mg; 0.09 mmol), 10% Pd/C (2 mg) and MeOH (4 mL). After stirring for 4 h under atmospheric pressure of hydrogen, the reaction mixture was filtered. The filtrate contained both fully aromatized debenzylated product **5b** and partially reduced debenzylated form **12b** as a side product. Therefore, the mixture was vigorously stirred in air until only fully aromatized product was present (ca. 2 h). The solution was allowed to stand overnight at −20 °C and the resulting precipitate was filtered off to afford **5b** (10 mg; 66%) as an orange crystalline solid. Mp.= 199–202 °C; TLC R_f_ = 0.2 (CHCl_3_/MeOH 40/2 *v*/*v*); ^1^H-NMR (400 MHz, DMSO-*d*_6_) δ = 9.88 (s, 1H), 9.51 (d, *J* = 0.5 Hz, 1H), 8.35 (d, *J* = 9.3 Hz, 1H), 8.11 (d, *J* = 9.1 Hz, 1H), 7.63 (d, *J* = 9.1 Hz, 1H), 7.48 (d, *J* = 9.3 Hz, 1H), 3.96 (s, 3H), 3.95 (s, 6H); ^13^C-NMR (101 MHz, DMSO-*d*_6_) δ = 151.0, 147.9, 144.2, 144.0, 143.1, 138.0, 126.6, 118.6, 118.2, 117.7, 115.8, 114.3, 112.5, 61.2, 56.7, 56.4; HRMS (HESI *m*/*z*): [M + H]^+^ calcd. for C_16_H_16_NO_4_ 286.1079; found 286.1074.

*3-Methoxy-*[1,3]*dioxolo[4,5-b]phenanthridin-4-ol* (**5c**). Compound **5k** (100 mg; 0.28 mmol) was added to ethanol (4 mL) containing aqueous 35% HCl (3 mL). This reaction mixture was heated in a sealed vial at 100 °C for 2.5 h. After removal of volatiles, the residue was diluted with water (5 mL) and alkalized with aqueous NH_3_ solution to pH = 8–9. The precipitated solid was filtered off, washed with water and dried to provide compound **5c** (57 mg; 76%) as an orange crystalline solid. A sample for analysis was prepared by crystallization from larger amount of THF. Mp. = 260–270 °C (decomp.); R_f_ = 0.2 (CHCl_3_/MeOH 40/2 *v*/*v*); ^1^H-NMR (400 MHz, DMSO-*d*_6_) δ = 9.85 (s, 1H), 9.39 (s, 1H), 8.14 (s, 1H), 8.11 (d, *J* = 8.7 Hz, 1H), 7.63 (d, *J* = 9.0 Hz, 1H), 7.43 (s, 1H), 6.21 (s, 2H), 3.96 (s, 3H); ^13^C-NMR (101 MHz, DMSO-*d*_6_) δ = 148.2, 147.84, 145.6, 143.8, 142.5, 140.04, 126.9, 119.5, 118.2, 116.56, 113.0, 106.9, 101.8, 100.0, 56.8; HRMS (HESI *m*/*z*): [M + H]^+^ calcd. for C_15_H_12_NO_4_, 270.0766; found 270.0760.

*7-Methoxy-*[1,3]*dioxolo[4,5-c]phenanthridin-6-ol* (**5d**). To a stirred solution of the secondary amine **4d** (300 mg; 0.85 mmol) and Bu_3_SnH (460 μL; 1.70 mmol) in anhydrous toluene (15 mL) at 70 °C was added AIBN (210 mg, 1.28 mmol) and temperature was raised and maintained at 104–108 °C for a period of 3 h. Due to no further conversion of starting material (**4d**), another portion of Bu_3_SnH (160 μL; 0.60 mmol) and AIBN (50 mg, 0.30 mmol) were added to the reaction mixture and heating continued for a further 3 h. The reaction mixture was then cooled to room temperature, activated MnO_2_ [44] (222 mg, 2.56 mmol) was added and stirring continued overnight. The suspension was filtered and the filtrate concentrated under reduced pressure. Addition of cyclohexane (30 mL) to the residue resulted in the exclusion of a red precipitate, which was filtered off and further purified by silica gel gradient column chromatography (hexane/EtOAc 5/1–5/3 *v*/*v*) to afford compound **5d** (30 mg; 13%) as an orange microcrystalline solid. Mp. = 246–248 °C; R_f_ = 0.2 (CHCl_3_/MeOH 40/2 *v*/*v*); ^1^H-NMR (400 MHz, DMSO-*d*_6_) δ = 9.99 (s, 1H), 9.44 (s, 1H), 8.16 (d, *J* = 8.8 Hz, 1H), 8.08 (d, *J* = 8.8 Hz, 1H), 7.63 (d, *J* = 8.8 Hz, 1H), 7.35 (d, *J* = 8.8 Hz, 1H), 6.25 (s, 2H), 3.95 (s, 3H); ^13^C-NMR (101 MHz, DMSO-*d*_6_) δ = 148.6, 146.1, 144.5, 143.3, 142.3, 129.3, 126.5, 119.5, 118.4, 115.8, 115.7, 112.7, 109.4, 102.0, 56.7; HRMS (HESI *m*/*z*): [M + H]^+^ calcd. for C_15_H_12_NO_4_, 270.0766; found 270.0761.

*2,3,7,8-Tetramethoxyphenanthridin* (**5e**). To a stirred solution of the secondary amine **4e** (600 mg, 1.57 mmol) and Bu_3_SnH (845 μL; 3.14 mmol) in anhydrous toluene (30 mL) at 70 °C was added AIBN (387 mg, 2.36 mmol) and temperature of the reaction mixture was raised and maintained at 104–108 °C for a period of 3 h. The reaction was carried out under an inert atmosphere of argon. After 3 h the reaction mixture was slowly cooled to room temperature and activated MnO_2_ (409 mg; 4.71 mmol) was added. The resulting mixture was stirred overnight, then filtered and concentrated under reduced pressure. Addition of cyclohexane (8 mL) to the residue resulted in the exclusion of a brown precipitate. The suspension was left to stand overnight at 5 °C, and the next day it was filtered, washed with cyclohexane (2 mL) and recrystallized from CHCl_3_/hexane. Compound **5e** (185 mg, 39%) was obtained as a beige crystalline solid. Mp. = 154–158 °C (CHCl_3_/hexane); R_f_ = 0.2 (CHCl_3_/MeOH 40/1 *v*/*v*); ^1^H-NMR (400 MHz, DMSO-*d*_6_) δ = 9.35 (s, 1H), 8.51 (d, *J* = 8.8 Hz, 1H), 8.03 (s, 1H), 7.74 (d, *J* = 9.1 Hz, 1H), 7.51 (s, 1H), 4.02 (s, 3H), 4.00 (s, 6H), 3.93 (s, 3H); ^13^C-NMR (101 MHz, DMSO-*d*_6_) δ = 150.1, 149.6, 148.9, 145.0, 144.1, 139.0, 126.4, 120.2, 118.9, 118.5, 117.6, 109.8, 102.6, 61.4, 56.6, 56.0, 55.6; HRMS (HESI *m*/*z*): [M + H]^+^ calcd. for C_17_H_18_NO_4_, 300.1236; found 300.1230.

*3,4,7,8-Tetramethoxyphenanthridine* (**5f**). Phenanthridine **5f** was prepared in a similar manner as the compound **5e** using amine **4f** (467 mg; 1.22 mmol), Bu_3_SnH (660 μL; 2.44 mmol), AIBN (301 mg, 1.83 mmol) and MnO_2_ (318 mg; 3.66 mmol) in anhydrous toluene (23 mL). The recrystallization from CHCl_3_/hexane afforded compound **5f** (150 mg; 41%) as a beige crystalline solid. Mp. = 142–144 °C; TLC R_f_ = 0.2 (CHCl_3_/MeOH 40/1 *v*/*v*); ^1^H-NMR (400 MHz, DMSO-*d*_6_) δ = 9.47 (s, 1H), 8.46 (d, *J* = 9.1 Hz, 1H), 8.42 (d, *J* = 9.1 Hz, 1H), 7.75 (d, *J* = 9.1 Hz, 1H), 7.52 (d, *J* = 9.1 Hz, 1H), 4.01 (s, 3H), 3.99 (s, 3H), 3.96 (s, 6H); ^13^C-NMR (101 MHz, DMSO-*d*_6_) δ = 151.2, 149.3, 147.5, 144.5, 144.0, 138.0, 126.7, 119.7, 119.3, 118.4, 118.2, 117.8, 114.6, 61.5, 61.3, 56.5, 56.4; HRMS (HESI *m*/*z*): [M + H]^+^ calcd. for C_17_H_18_NO_4,_ 300.1236; found 300.1230.

*3,4-Dimethoxy-*[1,3]*dioxolo[4,5-b]phenanthridine* (**5g**). Phenanthridine **5g** was prepared in a similar manner as compound **5e** using secondary amine **4g** (265 mg; 0.72 mmol), Bu_3_SnH (390 μL; 1.45 mmol), AIBN (178 mg, 1.09 mmol), MnO_2_ (189 mg; 2.17 mmol) in anhydrous toluene (13 mL). Compound **5g** (55 mg, 27%) was obtained as a beige microcrystalline solid. Mp. = 172–174 °C; TLC R_f_ = 0.2 (CHCl_3_/MeOH 40/1 *v*/*v*); ^1^H-NMR (400 MHz, DMSO-*d*_6_) δ = 9.33 (d, *J* = 0.5 Hz, 1H), 8.44 (d, *J* = 9.0 Hz, 1H), 8.20 (s, 1H), 7.73 (d, *J* = 9.3 Hz, 1H), 7.46 (s, 1H), 6.23 (s, 2H), 3.99 (s, 3H), 3.98 (s, 3H); ^13^C-NMR (101 MHz, DMSO-*d*_6_) δ = 149.0, 148.4, 148.1, 145.2, 143.9, 140.2, 126.9, 119.5, 119.5, 119.1, 118.7, 107.0, 102.0, 100.0, 61.4, 56.6; HRMS (HESI *m*/*z*): [M + H]^+^ calcd. for C_16_H_14_NO_4_, 284.0923; found 284.0917.

*6,7-Dimethoxy-*[1,3]*dioxolo[4,5-c]phenanthridine* (**5h**). Phenanthridine **5h** was prepared in a similar manner as the product **5e** using amine **4h** (478 mg; 1.31 mmol), Bu_3_SnH (700 μL; 2.61 mmol), AIBN (322 mg; 1.96 mmol) and MnO_2_ (340 mg; 3.92 mmol) in anhydrous toluene (23 mL). The recrystallization from CHCl_3_/hexane afforded compound **5h** (135 mg; 37%) as a brown crystalline solid. Mp. = 163–166 °C; TLC R_f_ = 0.2 (CHCl_3_/MeOH 40/1 *v*/*v*); ^1^H-NMR (400 MHz, DMSO-*d*_6_) δ = 9.40 (s, 1H), 8.42 (d, *J* = 9.1 Hz, 1H), 8.23 (d, *J* = 8.8 Hz, 1H), 7.75 (d, *J* = 9.0 Hz, 1H), 7.39 (d, *J* = 8.6 Hz, 1H), 6.27 (s, 2H), 4.00 (s, 3H), 3.99 (s, 3H); ^13^C-NMR (101 MHz, DMSO-*d*_6_) δ = 149.5, 148.1, 146.2, 144.7, 142.3, 129.1, 126.4, 122.3, 119.8, 119.5, 118.3, 115.7, 109.7, 102.1, 61.5, 56.5; HRMS (HESI *m*/*z*): [M + H]^+^ calcd. for C_16_H_14_NO_4_, 284.0923; found 284.0917.

*7-(Benzyloxy)-2,3,8-trimethoxyphenanthridine* (**5i**). Phenanthridine **5i** was prepared in a similar manner as phenanthridine **5e** using secondary amine **4i** (1.27 g, 2.78 mmol), Bu_3_SnH (1.5 mL; 5.55 mmol), AIBN (684 mg; 4.16 mmol) and MnO_2_ (724 mg, 8.33 mmol) in anhydrous toluene (50 mL). The recrystallization from CHCl_3_/hexane afforded compound **5i** (240 mg; 23%) as a beige crystalline solid. Mp. = 155–156 °C; TLC R_f_ = 0.2 (CHCl_3_/MeOH 40/1 *v*/*v*); ^1^H-NMR (400 MHz, DMSO-*d*_6_) δ = 9.25 (s, 1H), 8.51 (d, *J* = 9.1 Hz, 1H), 8.01 (s, 1H), 7.76 (d, *J* = 9.1 Hz, 1H), 7.53–7.49 (m, 2H), 7.46 (s, 1H), 7.41–7.30 (m, 3H), 5.25 (s, 2H), 4.04 (s, 3H), 4.01 (s, 3H), 3.92 (s, 3H); ^13^C-NMR (101 MHz, DMSO-*d*_6_) δ = 150.0, 149.5, 149.0, 145.2, 142.6, 138.9, 137.1, 128.6, 128.4, 128.2, 126.3, 120.5, 118.7, 118.5, 117.5, 109.8, 102.6, 75.0, 56.7, 56.0, 55.5; HRMS (HESI *m*/*z*): [M + H]^+^ calcd. for C_23_H_22_NO_4_, 376.1549; found 376.1543.

*7-(Benzyloxy)-3,4,8-trimethoxyphenanthridine* (**5j**). Phenanthridine **5j** was prepared in a similar manner as phenanthridine **5e** using secondary amine **4j** (950 mg, 2.07 mmol), Bu_3_SnH (1115 μL; 4.15 mmol), AIBN (511 mg, 3.11 mmol) and MnO_2_ (541 mg, 6.22 mmol) in anhydrous toluene (37 mL). The recrystallization from CHCl_3_/hexane afforded compound **5j** (233 mg; 30%) as a slightly gray microcrystalline solid. Mp. = 153–155 °C; TLC R_f_ = 0.3 (CHCl_3_/MeOH 40/1 *v*/*v*); ^1^H-NMR (400 MHz, DMSO-*d*_6_) δ = 9.38 (s, 1H), 8.45 (d, *J* = 8.8 Hz, 1H), 8.40 (d, *J* = 9.1 Hz, 1H), 7.77 (d, *J* = 9.1 Hz, 1H), 7.55–7.48 (m, 3H), 7.42–7.30 (m, 3H), 5.26 (s, 2H), 4.04 (s, 3H), 3.96 (s, 3H), 3.93 (s, 3H); ^13^C-NMR (101 MHz, DMSO-*d*_6_) δ = 151.2, 149.4, 147.6, 143.9, 143.0, 137.9, 137.0, 128.7, 128.4, 128.2, 126.6, 120.1, 119.1, 118.3, 118.2, 117.8, 114.5, 75.1, 61.3, 56.6, 56.4; HRMS (HESI *m*/*z*): [M + H]^+^ calcd. for C_23_H_22_NO_4_, 376.1549; found 376.1543.

*4-(Benzyloxy)-3-methoxy-*[1,3]*dioxolo[4,5-b]phenanthridine* (**5k**). Phenanthridine **5k** was prepared in a similar manner as phenanthridine **5e** using secondary amine **4k** (730 mg; 1.65 mmol), Bu_3_SnH (890 μL; 3.30 mmol), AIBN (407 mg, 2.48 mmol) and MnO_2_ (430 mg; 4.95 mmol) in anhydrous toluene (29 mL). The recrystallization from CHCl_3_/hexane afforded compound **5k** (280 mg; 47%) as a brown crystalline solid. Mp.= 129–131 °C; TLC R_f_ = 0.3 (CHCl_3_/MeOH 40/1 *v*/*v*); ^1^H-NMR (400 MHz, DMSO-*d*_6_) δ = 9.25 (s, 1H), 8.43 (d, *J* = 9.1 Hz, 1H), 8.18 (s, 1H), 7.76 (d, *J* = 9.1 Hz, 1H), 7.53–7.49 (m, 2H), 7.43–7.31 (m, 4H), 6.21 (s, 2H), 5.24 (s, 2H), 4.03 (s, 3H); ^13^C-NMR (101 MHz, DMSO-*d*_6_) δ = 149.1, 148.4, 148.0, 145.4, 142.4, 140.0, 137.0, 128.6, 128.4, 128.2, 126.7, 120.6, 119.3, 118.9, 118.6, 106.9, 101.9, 99.9, 75.0, 56.6; HRMS (HESI *m*/*z*): [M + H]^+^ calcd. for C_22_H_18_NO_4_, 360.1236; found 360.1230.

*6-(Benzyloxy)-7-methoxy-*[1,3]*dioxolo[4,5-c]phenanthridine* (**5l**). Phenanthridine **5l** was prepared in a similar manner as phenanthridine **5e** using secondary amine **4l** (1.1 g; 2.48 mmol), Bu_3_SnH (1.34 mL; 4.95 mmol), AIBN (610 mg, 3.72 mmol) and MnO_2_ (645 mg; 7.42 mmol) in anhydrous toluene (40 mL). The recrystallization from CHCl_3_/hexane afforded compound **5l** (555 mg; 62%) as a beige crystalline solid. Mp.= 163–164 °C; TLC R_f_ = 0.3 (CHCl_3_/MeOH 40/1 *v*/*v*); ^1^H-NMR (400 MHz, DMSO-*d*_6_) δ = 9.27 (s, 1H), 8.42 (d, *J* = 9.1 Hz, 1H), 8.21 (d, *J* = 8.7 Hz, 1H), 7.78 (d, *J* = 9.1 Hz, 1H), 7.50 (d, *J* = 6.9 Hz, 2H), 7.41 – 7.30 (m, 4H), 6.25 (s, 2H), 5.26 (s, 2H), 4.04 (s, 3H); ^13^C-NMR (101 MHz, DMSO-*d*_6_) δ = 149.62, 148.32, 146.25, 143.13, 142.34, 136.87, 129.06, 128.77, 128.40, 128.28, 126.38, 120.24, 119.31, 119.10, 118.41, 115.68, 109.71, 102.07, 75.07, 56.56; HRMS (HESI *m*/*z*): [M + H]^+^ calcd. for C_22_H_18_NO_4_, 360.1236; found 360.1230.

#### 3.1.3. General Procedure for the Preparation of Phenanthridine Hydrochlorides **6a**–**6l**

The above prepared phenanthridine derivatives **5** (ca. 20 mg) were dissolved in dioxane (2–3 mL). To the resulting solution a 4 M HCl in dioxane solution (1.5 mL) was added resulting in formation of a yellow-orange precipitate. To the suspension was added ether (4 mL). The precipitate was filtered off, washed with ether (2 mL) and dried under vacuum over KOH. The yields of hydrochlorides **6** were within 80–95%. All compounds were proved by elemental analysis; the recorded values were within +/−0.4%.

*7-Hydroxy-2,3,8-trimethoxyphenanthridin-5-ium* chloride (**6a**). Elemental analysis, calcd. for C_16_H_16_ClNO_4_ (321.8), C, 59.73; H, 5.01; N, 4.35; found C, 59.83; H, 5.11; N, 4.08.

*7-Hydroxy-3,4,8-trimethoxyphenanthridin-5-ium chloride* (**6b**). Elemental analysis, calcd. for C_16_H_16_ClNO_4_ (321.8), C, 59.73; H, 5.01; N, 4.35; found C, 59.74; H, 4.90; N, 4.18.

*4-Hydroxy-3-methoxy-*[1,3]*dioxolo[4,5-b]phenanthridin-6-ium chloride* (**6c**). Elemental analysis, calcd. for C_15_H_12_ClNO_4_ (305.7), C, 58.93; H, 3.96; N, 4.58; found C, 58.73; H, 3.77; N, 4.88.

*6-Hydroxy-7-methoxy-*[1,3]*dioxolo[4,5-c]phenanthridin-4-ium chloride* (**6d**). Elemental analysis, calcd. for C_15_H_12_ClNO_4_ (305.7), C, 58.93; H, 3.96; N, 4.58; found C, 58.65; H, 3.98; N, 4.73.

*2,3,7,8-Tetramethoxyphenanthridin-5-ium chloride* (**6e**). Elemental analysis, calcd. for C_17_H_18_ClNO_4_ (335.8), C, 60.81; H, 5.40; N, 4.17; found C, 61.15; H, 5.63; N, 4.26.

*3,4,7,8-Tetramethoxyphenanthridin-5-ium chloride* (**6f**). Elemental analysis, calcd. for C_17_H_18_ClNO_4_ (335.8), C, 60.81; H, 5.40; N, 4.17; found C, 61.01; H, 5.31; N, 4.02.

*3,4-Dimethoxy-*[1,3]*dioxolo[4,5-b]phenanthridin-6-ium chloride* (**6g**). Elemental analysis, calcd. for C_16_H_14_ClNO_4_ (319.7), C, 60.10; H, 4.41; N, 4.38; found C, 60.01; H, 4.71; N, 4.22.

*6,7-Dimethoxy-*[1,3]*dioxolo[4,5-c]phenanthridin-4-ium chloride* (**6h**). Elemental analysis, calcd. for C_16_H_14_ClNO_4_ (319.7), C, 60.10; H, 4.41; N, 4.38; found C, 60.24; H, 4.69; N, 4.48.

*7-(Benzyloxy)-2,3,8-trimethoxyphenanthridin-5-ium chloride* (**6i**). Elemental analysis, calcd. for C_23_H_22_ClNO_4_ (411.9), C, 67.07; H, 5.38; N, 3.40; found C, 67.17; H, 5.31; N, 3.46.

*7-(Benzyloxy)-3,4,8-trimethoxyphenanthridin-5-ium chloride* (**6j**). Elemental analysis, calcd. for C_23_H_22_ClNO_4_ (411.9), C, 67.07; H, 5.38; N, 3.40; found C, 67.22; H, 5.46; N, 3.20.

*4-(Benzyloxy)-3-methoxy-*[1,3]*dioxolo[4,5-b]phenanthridin-6-ium chloride* (**6k**). Elemental analysis, calcd. for C_22_H_18_ClNO_4_ (395.8), C, 66.75; H, 4.58; N, 3.54; found C, 66.66; H, 4.75; N, 3.44.

*6-(Benzyloxy)-7-methoxy-*[1,3]*dioxolo[4,5-c]phenanthridin-4-ium chloride* (**6l**). Elemental analysis, calcd. for C_22_H_18_ClNO_4_ (395.8), C, 66.75; H, 4.58; N, 3.54; found C, 66.58; H, 4.80; N, 3.32.

*4-Hydroxy-3-methoxy-6-methyl-*[1,3]*dioxolo[4,5-b]phenanthridin-6-ium iodide* (**7c**). The free base **5c** (20 mg; 0.08 mmol) was dissolved in ACN (10 mL) and MeI (19 μL; 0.3 mmol) was added. The reaction mixture was allowed to stand at r.t. in the dark place without stirring. After 7 days, the precipitated crystalline solid was filtered and washed with cold ACN (1 mL) to obtain poorly soluble substance **7c** (18 mg; 61%) as a dark orange crystalline solid. Mp. = 250–255 °C; ^1^H-NMR (400 MHz, DMSO-*d*_6_) δ = 11.35 (br. s, 1H), 9.95 (s, 1H), 8.50 (s, 1H), 8.35 (d, *J* = 9.0 Hz, 1H), 8.05 (d, *J* = 9.1 Hz, 1H), 7.97 (s, 1H), 6.41 (s, 2H), 4.56 (s, 3H), 4.05 (s, 3H); ^13^C-NMR (101 MHz, DMSO-*d*_6_) δ = 150.7, 150.1, 147.42, 145.7, 145.2, 130.22, 127.6, 124.0, 122.7, 114.6, 113.5, 103.71, 101.4, 98.6, 57.0, 46.1; HRMS (HESI *m*/*z*): [M − I]^+^ calcd. for C_16_H_14_NO_4_, 284.0923; found 284.0917; elemental analysis, calcd. for C_16_H_14_INO_4_ (411.2), C, 46.74; H, 3.43; N, 3.41; found C, 46.70; H, 3.66; N, 3.35.

*6-Hydroxy-7-methoxy-4-methyl-*[1,3]*dioxolo[4,5-c]phenanthridin-4-ium iodide* (**7d**). The quaternary salt **7d** was prepared in a similar manner as compound **7c** using base **5d** (10 mg; 0.04 mmol), MeI (9 μL; 0.15 mmol) and ACN (3 mL). Compound **7d** (9 mg; 61%) was obtained as a dark orange crystalline solid. Mp. = 236–239 °C; ^1^H-NMR (400 MHz, DMSO-*d*_6_) δ = 11.52 (br. s, 1H), 9.90 (s, 1H), 8.49 (d, *J* = 8.6 Hz, 1H), 8.26 (d, *J* = 8.8 Hz, 1H), 8.00 (d, *J* = 8.8 Hz, 1H), 7.69 (dd, *J* = 1.0, 8.8 Hz, 1H), 6.38 (s, 2H), 4.65 (s, 3H), 4.04 (s, 3H); ^13^C-NMR (101 MHz, DMSO-*d*_6_) δ = 152.0, 148.8, 147.4, 145.7, 138.1, 126.9, 124.2, 120.9, 120.0, 118.4, 113.7, 113.0, 112.4, 103.1, 56.8, 48.2; HRMS (HESI *m*/*z*): [M − I]^+^ calcd. for C_16_H_14_NO_4_, 284.0923; found 284.0917; elemental analysis, calcd. for C_16_H_14_INO_4_ (411.2), C, 46.74; H, 3.43; N, 3.41; found C, 46.88; H, 3.54; N, 3.32.

*2,3,7,8-Tetramethoxy-5-methylphenanthridinium iodide* (**7e**). The quaternary salt **7e** was prepared in a similar manner as compound **7d** using base **5e** (280 mg, 0.94 mmol), MeI (230 μL; 3.74 mmol) and ACN (14 mL). Compound **7e** (372 mg; 90%) was obtained as a dark orange needle-like crystalline solid. Mp. = 263–266 °C; ^1^H-NMR (400 MHz, DMSO-*d*_6_) δ = 9.96 (s, 1H), 8.84 (d, *J* = 9.3 Hz, 1H), 8.33 (s, 1H), 8.20 (d, *J* = 9.3 Hz, 1H), 7.73 (s, 1H), 4.70 (s, 3H), 4.13 (s, 6H), 4.10 (s, 6H); ^13^C-NMR (101 MHz, DMSO-*d*_6_) δ = 152.0, 151.5, 149.9, 146.9, 145.6, 129.1, 127.7, 125.3, 120.8, 119.2, 118.7, 103.9, 100.7, 62.1, 57.0, 56.8, 56.6, 46.0; HRMS (HESI *m*/*z*): [M − I]^+^ calcd. for C_18_H_20_NO_4_, 314.1392; found 314.1387; elemental analysis, calcd. for C_18_H_20_INO_4_ (441.3), C, 48.99; H, 4.57 N, 3.17; found C, 48.92; H, 4.15; N, 3.12.

*3,4,7,8-Tetramethoxy-5-methylphenanthridinium iodide* (**7f**). The quaternary salt **7f** was prepared in a similar manner as compound **7d** using base **5f** (60 mg, 0.20 mmol), MeI (50 μL, 0.80 mmol) and ACN (5 mL). Compound **7f** (42 mg; 47%) was obtained as an orange crystalline solid. Mp. = 186–188 °C; ^1^H-NMR (400 MHz, DMSO-*d*_6_) δ = 9.95 (s, 1H), 8.81 (d, *J* = 9.3 Hz, 1H), 8.70 (d, *J* = 9.3 Hz, 1H), 8.19 (d, *J* = 9.3 Hz, 1H), 7.89 (d, *J* = 9.3 Hz, 1H), 4.81 (s, 3H), 4.13 (s, 3H), 4.09 (s, 3H), 4.08 (s, 3H), 3.99 (s, 3H); ^13^C-NMR (101 MHz, DMSO-*d*_6_) δ = 154.4, 153.6, 150.0, 146.4, 140.1, 128.3, 128.1, 126.2, 120.5, 120.2, 118.5, 118.0, 116.9, 62.2, 61.8, 56.9, 51.1, 39.5; HRMS (HESI *m*/*z*): [M − I]^+^ calcd. for C_18_H_20_NO_4_, 314.1392; found 314.1387.

*3,4-Dimethoxy-6-methyl-*[1,3]*dioxolo[4,5-b]phenanthridin-6-ium iodide* (**7g**). The quaternary salt **7g** was prepared in a similar manner as substance **7d** using base **5g** (30 mg, 0.11 mmol), MeI (25 μL; 0.42 mmol) and ACN (6 mL). Compound **7g** (40 mg; 89%) was obtained as a bright yellow crystalline solid. Mp. > 300 °C; ^1^H-NMR (400 MHz, DMSO-*d*_6_) δ = 9.96 (s, 1H), 8.71 (d, *J* = 9.3 Hz, 1H), 8.58 (s, 1H), 8.19 (d, *J* = 9.3 Hz, 1H), 8.04 (s, 1H), 6.44 (s, 2H), 4.62 (s, 3H), 4.11 (s, 3H), 4.08 (s, 3H); ^13^C-NMR (101 MHz, DMSO-*d*_6_) δ = 151.1, 150.4, 150.0, 147.2, 145.5, 130.6, 128.1, 125.5, 122.8, 119.1, 118.7, 103.9, 101.2, 98.5, 62.1, 57.0, 46.4; HRMS (HESI *m*/*z*): [M − I]^+^ calcd. for C_17_H_16_NO_4_, 298.1079; found 298.1074.

*6,7-Dimethoxy-4-methyl-*[1,3]*dioxolo[4,5-c]phenanthridin-4-ium iodide* (**7h**). The quaternary salt **7h** was prepared in a similar manner as the substance **7d** using free base **5h** (90 mg; 0.32 mmol), MeI (80 μL; 1.27 mmol) and ACN (5 mL). Compound **7h** (26 mg; 19%) was obtained as a blood-red crystalline solid. Mp. = 223–224 °C; ^1^H-NMR (400 MHz, DMSO-*d*_6_) δ = 9.95 (s, 1H), 8.64 (d, *J* = 9.1 Hz, 1H), 8.59 (d, *J* = 8.8 Hz, 1H), 8.16 (d, *J* = 9.3 Hz, 1H), 7.76 (d, *J* = 8.8 Hz, 1H), 6.42 (s, 2H), 4.71 (s, 3 H), 4.11 (s, 3H), 4.07 (s, 3H); ^13^C-NMR (101 MHz, DMSO-*d*_6_) δ = 152.1, 150.2, 149.1, 146.7, 138.2, 127.6, 126.0, 120.9, 120.2, 118.7, 118.6, 118.0, 112.8, 103.3, 62.2, 56.9, 48.7; HRMS (HESI *m*/*z*): [M − I]^+^ calcd. for C_17_H_16_NO_4_, 298.1079; found 298.1074.

*7-(Benzyloxy)-2,3,8-trimethoxy-5-methylphenanthridinium iodide* (**7i**). The quaternary salt **7i** was prepared in a similar manner as compound **7d** using free base **5i** (91 mg; 0.24 mmol), MeI (60 μL; 0.97 mmol) and ACN (9 mL). Compound **7i** (77 mg; 61%) was obtained as a bright yellow crystalline solid. Mp. = 207–210 °C; ^1^H-NMR (400 MHz, DMSO-*d*_6_) δ = 9.79 (s, 1H), 8.83 (d, *J* = 9.3 Hz, 1H), 8.30 (s, 1H), 8.20 (d, *J* = 9.1 Hz, 1H), 7.70 (s, 1H), 7.58 (d, *J* = 7.5 Hz, 2H), 7.43–7.30 (m, 3H), 5.40 (s, 2H), 4.68 (s, 3H), 4.12 (s, 6H), 4.09 (s, 3H); ^13^C-NMR (101 MHz, DMSO-*d*_6_) δ = 152.0, 151.5, 150.2, 146.7, 143.9, 136.3, 128.9, 128.9, 128.4, 128.3, 127.5, 125.0, 120.7, 119.4, 119.1, 103.9, 100.7, 75.4, 57.0, 56.7, 56.6, 46.2; HRMS (HESI *m*/*z*): [M − I]^+^ calcd. for C_24_H_24_NO_4_, 390.1705; found 390.1700.

*7-(Benzyloxy)-3,4,8-trimethoxy-5-methylphenanthridinium iodide* (**7j**). The quaternary salt **7j** was prepared in a similar manner as compound **7d** using free base **5j** (150 mg; 0.40 mmol), MeI (100 μL; 1.6 mmol) and ACN (7 mL). Compound **7j** (123 mg; 60%) was obtained as a light orange microcrystalline solid. Mp. = 169–173 °C; ^1^H-NMR (400 MHz, DMSO-*d*_6_) δ = 9.79 (s, 1H), 8.80 (d, *J* = 9.3 Hz, 1H), 8.70 (d, *J* = 9.1 Hz, 1H), 8.20 (d, *J* = 9.1 Hz, 1H), 7.89 (d, *J* = 9.3 Hz, 1H), 7.59 (d, *J* = 7.0 Hz, 2 H), 7.48–7.26 (m, 3H), 5.41 (s, 2H), 4.79 (s, 3H), 4.10 (s, 3H), 4.08 (s, 3H), 3.98 (s, 3H); ^13^C-NMR (101 MHz, DMSO-*d*_6_) δ = 154.4, 153.5, 150.3, 144.6, 140.1, 136.3, 128.9, 128.4, 128.3, 128.2, 127.9, 126.0, 120.4, 120.2, 118.8, 118.5, 117.0, 75.6, 61.8, 57.0, 56.9, 51.3; HRMS (HESI *m*/*z*): [M − I]^+^ calcd. for C_24_H_24_NO_4_, 390.1705; found 390.1700.

*4-(Benzyloxy)-3-methoxy-6-methyl-*[1,3]*dioxolo[4,5-b]phenanthridin-6-ium iodide* (**7k**). The quaternary salt **7k** was prepared in a similar manner as compound **7d** using free base **5k** (91 mg, 0.25 mmol), MeI (60 μL, 1.0 mmol) and ACN (8 mL). The reaction was conducted at 50 °C for 36 h. Compound **7k** (77 mg; 61%) was obtained as a pale yellow crystalline solid. Mp. = 198–201 °C; ^1^H-NMR (400 MHz, DMSO-*d*_6_) δ = 9.81 (s, 1H), 8.69 (d, *J* = 9.1 Hz, 1H), 8.55 (s, 1H), 8.19 (d, *J* = 9.3 Hz, 1H), 8.01 (s, 1H), 7.61–7.56 (m, 2H), 7.42–7.31 (m, 3H), 6.44 (s, 2H), 5.38 (s, 2H), 4.60 (s, 3H), 4.10 (s, 3H); ^13^C-NMR (101 MHz, DMSO-*d*_6_) δ = 151.1, 150.4, 150.2, 147.0, 143.7, 136.3, 130.5, 128.9, 128.4, 128.3, 128.0, 125.3, 122.8, 119.2, 119.2, 103.9, 101.3, 98.5, 75.4, 56.9, 46.7; HRMS (HESI *m*/*z*): [M − I]^+^ calcd. for C_23_ H_20_NO_4_, 374.1392; found 374.1387.

*6-(Benzyloxy)-7-methoxy-4-methyl-*[1,3]*dioxolo[4,5-c]phenanthridin-4-ium iodide* (**7l**). The quaternary salt **7l** was prepared in a similar manner as compound **7d** with using free base **5l** (36 mg, 0.1 mmol), MeI (24 μL, 0.4 mmol) and ACN (4 mL). The reaction was conducted at 50 °C for 36 h. Compound **7l** (28 mg; 55%) was obtained as a light orange solid. Mp. = 169–172 °C; ^1^H-NMR (400 MHz, DMSO-*d*_6_) δ = 9.80 (s, 1H), 8.64 (d, *J* = 9.2 Hz, 1H), 8.58 (d, *J* = 8.7 Hz, 1H), 8.17 (d, *J* = 9.2 Hz, 1H), 7.75 (d, *J* = 8.7 Hz, 1H), 7.64–7.52 (m, 2H), 7.44–7.28 (m, 3H), 6.40 (s, 2H), 5.39 (s, 2H), 4.69 (s, 3H), 4.08 (s, 3H); ^13^C-NMR (101 MHz, DMSO-*d*_6_) δ = 152.0, 150.2, 149.2, 145.0, 138.2, 136.2, 128.9, 128.5, 128.4, 127.6, 125.8, 120.8, 120.2, 119.0, 118.6, 118.5, 112.9, 103.3, 75.6, 56.9, 48.9; HRMS (HESI *m*/*z*): [M − I]^+^ calcd. for C_23_ H_20_NO_4_, 374.1392; found 374.1387.

#### 3.1.4. Preparation of 2,3,7,8-tetramethoxy-5-methylphenanthridinium Salts **9t**–**9z**

To a suspension of quaternary iodide **7e** (54 mg; 0.11 mmol) in EtOH (18 mL) was added 1.25 N aqueous NaOH (180 μL; 0.22 mmol). After 1 h of stirring the resulting colorless solution was diluted with water (2 mL) and concentrated under reduced pressure to remove EtOH resulting in precipitation of a white crystalline solid (pseudobase **8**), which was not isolated. To the suspension of pseudobase was added 20% aqueous solution of corresponding acid (10 mL) and the mixture was stirred for 10 min, and then cooled to 0 °C. The resulting yellow precipitate was filtered, washed with ice water (2 mL) and dried at 110 °C.

*2,3,7,8-Tetramethoxy-5-methylphenanthridinium chloride* (**9t**): Yield 35 mg (82%) as a yellow microcrystalline compound. Mp. = 220–222 °C; ^1^H-NMR (400 MHz, DMSO-*d*_6_) δ = 9.96 (s, 1H), 8.83 (d, *J* = 9.2 Hz, 1H), 8.29 (s, 1H), 8.17 (d, *J* = 9.3 Hz, 1H), 7.72 (s, 1H), 4.71 (s, 3H), 4.12 (s, 3H), 4.11 (s, 3H), 4.10 (s, 3H), 4.09 (s, 3H); ^13^C-NMR (101 MHz, DMSO-*d*_6_) δ = 152.0, 151.5, 149.9, 146.8, 145.6, 129.1, 127.6, 125.2, 120.8, 119.18, 118.68, 103.9, 100.7, 62.1, 57.1, 56.7, 56.6, 46.0; elemental analysis, calcd. for C_18_H_20_ClNO_4_ (349.8), C, 61.80; H, 5.76; N, 4.00; found C, 61.83; H, 5.69; N, 4.03.

*2,3,7,8-Tetramethoxy-5-methylphenanthridinium perchlorate* (**9u**): Yield 40 mg (80%) as a light orange microcrystalline compound. Mp. = above 300 °C; ^1^H-NMR (400 MHz, DMSO-*d*_6_) δ = 9.93 (s, 1H), 8.80 (d, *J* = 9.2 Hz, 1H), 8.28 (s, 1H), 8.17 (d, *J* = 9.3 Hz, 1H), 7.70 (s, 1H), 4.69 (s, 3H), 4.12 (s, 3H), 4.12 (s, 3H), 4.09 (s, 6H); ^13^C-NMR (101 MHz, DMSO-*d*_6_) δ = 152.0, 151.5, 149.9, 146.8, 145.6, 129.1, 127.6, 125.2, 120.8, 119.18, 118.68, 103.9, 100.7, 62.1, 57.1, 56.7, 56.6, 46.0; elemental analysis, calcd. for C_18_H_20_ClNO_8_ (413.8), C, 52.24; H, 4.87; N, 3.38; found C, 52.12; H, 4.68; N, 3.35.

*2,3,7,8-Tetramethoxy-5-methylphenanthridinium hydrogensulfate* (**9w**): Yield 38 mg (75%) as a yellow microcrystalline compound. Mp. = 272–280 °C; ^1^H-NMR (400 MHz, DMSO-*d*_6_) δ = 9.94 (s, 1H), 8.82 (d, *J* = 9.2 Hz, 1H), 8.30 (s, 1H), 8.18 (d, *J* = 9.3 Hz, 1H), 7.72 (s, 1H), 4.70 (s, 3H), 4.12 (s, 3H), 4.12 (s, 3H), 4.10 (s, 3H), 4.09 (s, 3H); ^13^C-NMR (101 MHz, DMSO-*d*_6_) δ = 152.0, 151.5, 149.9, 146.8, 145.6, 129.1, 127.6, 125.2, 120.8, 119.18, 118.68, 103.9, 100.7, 62.1, 57.1, 56.7, 56.6, 46.0; elemental analysis, calcd. for C_18_H_21_NO_8_S (411.4), C, 52.55; H, 5.14; N, 3.40; found C, 52.40; H, 5.24; N, 3.43.

*2,3,7,8-Tetramethoxy-5-methylphenanthridinium nitrate* (**9z**): Yield 37 mg (80%) as a light orange microcrystalline compound. Mp. = 266–269 °C; ^1^H-NMR (400 MHz, DMSO-*d*_6_) δ = 9.95 (s, 1H), 8.83 (d, *J* = 9.2 Hz, 1H), 8.32 (s, 1H), 8.19 (d, *J* = 9.3 Hz, 1H), 7.72 (s, 1H), 4.70 (s, 3H), 4.12 (s, 6H), 4.10 (s, 6H); ^13^C-NMR (101 MHz, DMSO-*d*_6_) δ = 152.0, 151.5, 149.9, 146.8, 145.6, 129.1, 127.6, 125.2, 120.8, 119.18, 118.68, 103.9, 100.7, 62.1, 57.1, 56.7, 56.6, 46.0; elemental analysis, calcd. for C_18_H_20_N_2_O_7_ (376.1), C, 57.44; H, 5.36; N, 7.44; found C, 57.36; H, 5.45; N, 7.48.

### 3.2. Antibacterial Activity Testing

#### 3.2.1. Agar Diffusion Test

Overnight cultures of test organisms were grown in LB broth for 18–24 h and standard suspensions of 1.5 × 10^8^ CFU/mL were prepared in a sterile saline solution (0.9% NaCl) according to a BaSO_4_ 0.5 McFarland Standard. The standardized suspension (0.1 mL) was added to 34 mL of sterile, melted and tempered (47–50 °C) Mueller-Hinton No. 2 agar. After gentle mixing, the inoculated melted agar was poured into a sterile Petri dish (145 mm × 20 mm, Greiner Bio-One, Kremsmünster, Austria) and allowed to solidify next to the flame with lids slightly ajar. Wells of 9 mm diameter were cut from the Petri dish agar and filled with 50 μL of the test sample solution. Solutions were made at 20 mM in DMSO and diluted in MeOH to 2 mM. The Petri dish was incubated at 37 °C for 18–24 h and the inhibition zone diameters were measured (mm) with an electronic caliper after 24–48 h.

#### 3.2.2. MIC Determination

Antibacterial activity of the compounds was determined by measuring their minimum inhibitory concentrations (MIC) using the broth microdilution method. Each well of a 96-well microtiter plate was filled with 50 μL of sterile iron deficient MH2 broth. Each test compound was dissolved in DMSO making a 10 mM solution, then diluted with sterile MH2 broth to 800 µM. Exactly 50 μL of the compound solution was added to the first well of the microtiter plate and 2-fold serial dilutions were made down each row of the plate. A pre culture of bacteria was grown in Luria-Bertani broth overnight at 37 °C. This was diluted to McFarland standard 0.5 (1.5 × 10^8^ CFU) with saline. 100 µL of the bacterial suspension was further diluted with 14.9 mL of MHII broth. Exactly 50 μL of bacterial (in broth) inoculum (1 × 10^6^ CFU/mL) was then added to each well giving a total volume of 100 μL/well and 5 × 10^5^ CFU/mL and a compound concentration gradient of 200–0.1 µM. The plate was incubated at 37 °C. After 18 h, each well was examined visually for bacterial growth. The MIC was recorded as the lowest compound concentration required to inhibit bacterial growth as judged by turbidity relative to a row of wells diluted with a the solvent DMSO as a growth control. Ciprofloxacin was included in a control row at a concentration gradient of 5 µg/mL–0.0025 µg/L.

### 3.3. Anticancer Activity In Vitro

The in vitro anticancer activity was determined using MCF-7 (breast adenocarcinoma) and K-562 (chronic myelogeneous leukemia) cell lines as described earlier [48]. Briefly, cells were treated in triplicate with three different doses of each compound for 72 h. After treatments, Calcein AM solution was added, and fluoresence from live cells was measured at 485 nm/538 nm (excitation/emission) using a Fluoroskan Ascent microplate reader (Thermo Scientific, Waltham, MA, USA). The EC_50_ value, that is, the drug concentration reducing number of live cells to 50%, was calculated from the dose response curves that resulted from the assays. The cells were maintained in DMEM medium supplemented with 10% fetal bovine serum, penicillin (100 U/mL), and streptomycin (100 µg/mL) and cultivated at 37 °C in 5% CO_2_.

#### 3.3.1. Cell Cycle Analysis

Cell cycle analysis was performed as described earlier [48]. Briefly, subconfluent cells were treated with different concentrations of test compound for 24 h. The cultures were pulse-labeled with 10 μM 5-bromo-2′-deoxyuridine (BrdU) for 30 min at 37 °C prior to harvesting. The cells were then washed in PBS, fixed with 70% ethanol, and denatured in 2 M HCl. Following neutralization, the cells were stained with anti-BrdU fluorescein-labeled antibodies, washed, stained with propidium iodide, and analyzed by flow cytometry using a 488 nm laser (FACSVerse, Becton Dickinson, Franklin Lakes, NJ, USA).

#### 3.3.2. Immunoblotting

Immunoblotting was performed as described earlier [48]. Briefly, cellular lysates were prepared by harvesting cells in Laemmli sample buffer. Proteins were separated on SDS-polyacrylamide gels and electroblotted onto nitrocellulose membranes. After blocking, the membranes were incubated with specific primary antibodies overnight, washed and then incubated with peroxidase-conjugated secondary antibodies. Finally, peroxidase activity was detected with ECL+ reagents (AP Biotech, Prague, Czech Republic) using a LAS-4000 CCD camera (AP Biotech, Prague, Czech Republic). Specific antibodies against PARP were purchased from Santa Cruz Biotechnology (Dallas, TX, USA) peroxidase-labeled secondary antibodies was from Sigma Aldrich (Prague, Czech Republic, peroxidase-labeled secondary antibodies) or were generously gifted by Dr. B. Vojtěšek (p53, p21WAF1, PCNA).

## 4. Conclusions

In this work we extended a previously developed method for the preparation of novel phenanthridine derivatives, which were derived from benzo[*c*]phenanthridines by deletion of the A-ring. Derivatives were prepared with substituents on the formed skeleton that mimic biologically active benzo[*c*]phenanthridines. The main principle for the preparation of the aforementioned compounds was based on the radical cyclization of reduced Schiff bases prepared by the condensation of appropriate aromatic aldehydes and amines. The prepared compounds were tested for antibacterial and anticancer cytotoxic effects and compared with several compounds containing a benzo[*c*]phenanthridine skeleton (e.g., chelerythrine, sanguinarine, isodecarine, norchelerythrine, NK-109). Several derivatives displayed antibacterial activity against *Bacillus subtilis*, *Micrococcus luteus* and/or *Mycobacterium vaccae* and cytotoxicity against K-562 and MCF-7 cancer cell lines at micromolar concentrations; these compounds typically contained a *N*-methylated quaternary nitrogen and 7-benzyloxy substitution. The mechanism of action of the novel phenanthridines however remains still unclear.

## Figures and Tables

**Table 1:** Numbering of compounds **3**–**7**.

Compound Number						Type of Substitution			
3	4	5	6	7		R^1^	R^2^	R^3^	R^4^
•	•	•	•		a:	-OCH_3_	-OCH_3_	H	H
		•	•		b:	H	-OCH_3_	-OCH_3_	H
•	•	•	•	•	c:	-OCH_2_O-		H	H
•	•	•	•	•	d:	H	-OCH_2_O-		H
	•	•	•	•	e:	-OCH_3_	-OCH_3_	H	CH_3_
	•	•	•	•	f:	H	-OCH_3_	-OCH_3_	CH_3_
	•	•	•	•	g:	-OCH_2_O-		H	CH_3_
	•	•	•	•	h:	H	-OCH_2_O-		CH_3_
	•	•	•	•	i:	-OCH_3_	-OCH_3_	H	benzyl
	•	•	•	•	j:	H	-OCH_3_	-OCH_3_	benzyl
	•	•	•	•	k:	-OCH_2_O-		H	benzyl
	•	•	•	•	l:	H	-OCH_2_O-		benzyl

• Newly prepared compounds (for general structures see Scheme 1).

**Table 2:** Antibacterial activity.

Compound	Diameter of Inhibition Zone in Agar Diffusion Assay (mm) (MIC) (μmol L^−1^)					
	*B. subtilis* (ATCC 6633)	*M. luteus* (ATCC 10240)	*M. vaccae* (DSM 43514)	*S. aureus* (CCM 2524)	*P. aeruginosa* (CCM 3955)	*E. coli* (CCM 3954)
**5a**	13	14	15	15	15	12
**5b**	12	15	20(>200)	19	20(>200)	0
**5c**	18	17	25(50)	18	16	0
**5d**	33(50)	22(50)	23(25)	18	15	0
**5e**	22(>200)	18	23(200)	13	12	12
**5f**	35(50)	30(100)	31(25)	20(50)	15	13
**5g**	20(100)	14	16	11	12	0
**5h**	34(50)	30(100)	22(25)	20(50)	15	0
**5i**	28(50)	22(50)	18	17	15	0
**5j**	19	18	17	19	13	12
**5k**	41(6.25)	33(3.13)	31(12.5)	24(25)	16	11
**5l**	34(12.5)	30(3.13)	32(12.5)	20(50)	15	0
**6e**	0	13	19	13	15	14
**6f**	11	16	12	0	15	15
**6g**	11	16	12	0	15	15
**6h**	0	15	14	12	16	11
**6i**	14	16	13	11	18	0
**6j**	11	16	12	0	18	10
**6k**	12	15	14	14	17	0
**6l**	12	10	10	14	0	0
**7c**	13	20	10	15	0	0
**7d**	13	19	21(100)	15	0	0
**7e**	11	26(50)	11	12	14	13
**7f**	13	36(25)	21(50)	17	13	12
**7g**	39(6.25)	48(12.5)	18	15	14	12
**7h**	26(25)	40(50)	15	14	15	11
**7i**	25(25)	42(3.13)	40(6.25)	13	10	0
**7j**	35(12.5)	42(0.78)	40(12.5)	13	12	0
**7k**	30(12.5)	42(3.13)	30(3.13)	20(50)	0	12
**7l**	30(25)	37(3.13)	30(3.13)	18	0	0
**9t**	15	25(50)	21(50)	15	10	10
**9u**	15	20(25)	25(50)	18	13	10
**9w**	17	20(50)	21(50)	17	10	12
**9z**	15	33(25)	21(50)	17	13	10
**13**	15	12	16	15	0	0
**14**	25(0.78)	22(6.25)	23(0.78)	24(0.78)	0	15
norchelerythrine	14	11	17	18	0	0
isodecarine	16	11	12	16	0	0
NK-109	32(1.56)	27(12.5)	45(12.5)	25(12.5)	0	15
chelerythrine	30(3.13)	26(50)	32(3.13)	24(6.25)	0	16
sanguinarine	29(3.13)	27(25)	27(6.25)	24(6.25)	0	17
Ciprofloxacin *	20(7.5)	22(0.26)	27(0.26)	20(15.0)	15(3.75)	20(7.5)

* used as a standard.

**Table 3:** Anticancer activity in vitro.

Compounds	EC_50_ (µM) *	
	K-562	MCF-7
**3a**	>50	>50
**3c**	>50	>50
**3d**	>50	>50
**4a**	>50	>50
**4c**	71 ± 2	79 ± 1
**4d**	>50	>50
**4e**	40 ± 8	70 ± 5
**4f**	>25	>25
**4g**	51 ± 5	75 ± 2
**4h**	>12	>12
**4i**	>50	>50
**4j**	>25	>25
**4k**	>50	>50
**4l**	>50	>50
**5a**	11 ± 1	17 ± 6
**5b**	40 ± 5	24 ± 4
**5c**	59 ± 4	17 ± 1
**5d**	19 ± 6	12 ± 1
**5e**	29 ± 6	27 ± 6
**5f**	13 ± 1	10 ± 2
**5g**	8.7 ± 1.3	7.2 ± 2.9
**5h**	14 ± 1	10 ± 1
**5i**	19 ± 8	15 ± 5
**5j**	36 ± 11	23 ± 5
**5k**	4.2 ± 1.1	3.2 ± 0.9
**5l**	>50	>50
**6a**	8.7 ± 1.2	18 ± 3
**6c**	60 ± 8	17 ± 4
**6d**	>50	>50
**6e**	24 ± 1	34 ± 6
**6f**	82 ± 7	39 ± 3
**6g**	15 ± 3	20 ± 2
**6h**	59 ± 12	45 ± 6
**6i**	67 ± 18	43 ± 9
**6j**	14 ± 1	21 ± 2
**6k**	>50	>50
**6l**	>50	>50
**7c**	82 ± 10	57 ± 7
**7d**	41 ± 4	16 ± 1
**7e**	>50	38 ±
**7f**	81 ± 17	69 ± 7
**7g**	29 ± 13	11 ± 6
**7h**	>50	>50
**7i**	6.4 ± 1.1	5.3 ± 1.1
**7j**	0.8 ± 0.2	2.6 ± 0.3
**7k**	5.1 ± 0.7	1.8 ± 0.8
**7l**	8.3 ± 1.4	7.7 ± 0.9
**9t**	>50	>50
**9u**	>50	45 ± 4
**9w**	>50	36 ± 9
**9z**	>50	>50
**13**	94 ± 6	48 ± 2
**14**	0.9 ± 0.2	1.4 ± 0.1
norchelerythrine	>100	>100
isodecarine	>100	>100
NK-109	1.0 ± 0.1	4.6 ± 0.6
chelerythrine	8.6 ± 1.8	5.9 ± 0.5
sanguinarine	2.2 ± 0.2	2.0 ± 0.2
cisplatin	4.7 ± 0.4	11 ± 1

* Values are means of triplicates and indicate concentration that kills 50% of cells during a three-day cultivation.

## Supplementary Materials

The copies of ^1^H- and ^13^C-NMR spectra are available online.
