# Supplementary material for: Synthesis, Bacteriostatic and Anticancer Activity of Novel Phenanthridines Structurally Similar to Benzo[c]phenanthridine Alkaloids
Source: Molecules. 2018 Aug 27;23(9):2155. doi: 10.3390/molecules23092155 (PMC6225299; doi:10.3390/molecules23092155)
Supplement: Supplementary file 1 [file molecules-23-02155-s001.pdf]

Supporting Information

Synthesis, Bacteriostatic and Anticancer Activity of Novel Phenanthridines Structurally Similar to Benzo[*c*]phenanthridine Alkaloids

Pavel Lasák [1], Kamil Motyka [2], Vladimír Kryštof [3] and Jakub Stýskala [1,*]

[1] Department of Organic Chemistry, Faculty of Science, Palacký University, 17. listopadu 1192/12, CZ-771 46 Olomouc, Czech Republic; pavellasak@seznam.cz

[2] Institute of Molecular and Translation Medicine, Faculty of Medicine, Palacký University, Hněvotínská 5, CZ-779 00 Olomouc, Czech Republic; kamil.motyka@gmail.com

[3] Laboratory of Growth Regulators, Faculty of Science, Palacký University and Institute of Experimental Botany ASCR, Šlechtitelů 27, CZ-783 71 Olomouc, Czech Republic; vladimir.krystof@upol.cz

* Correspondence: jakub.styskala@upol.cz; Tel.: +42-058-563-4466

Compound 3a ($CDCl_3$)

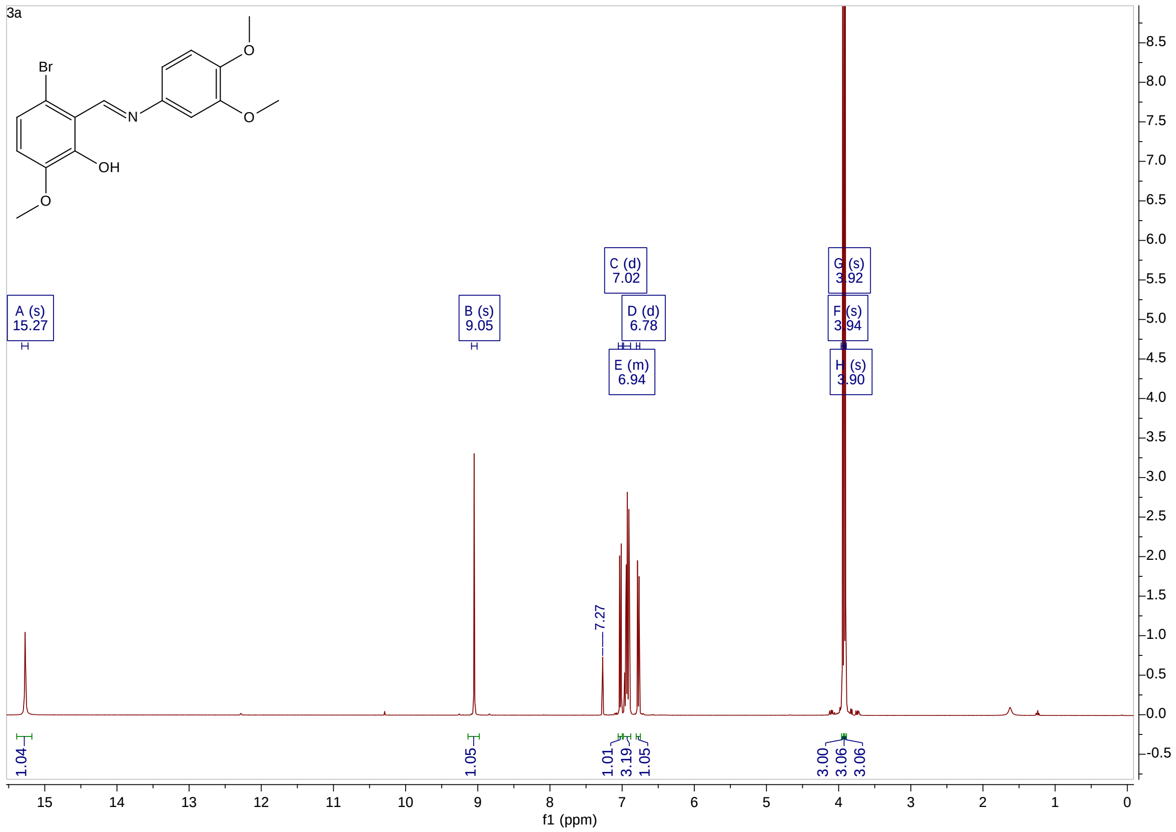

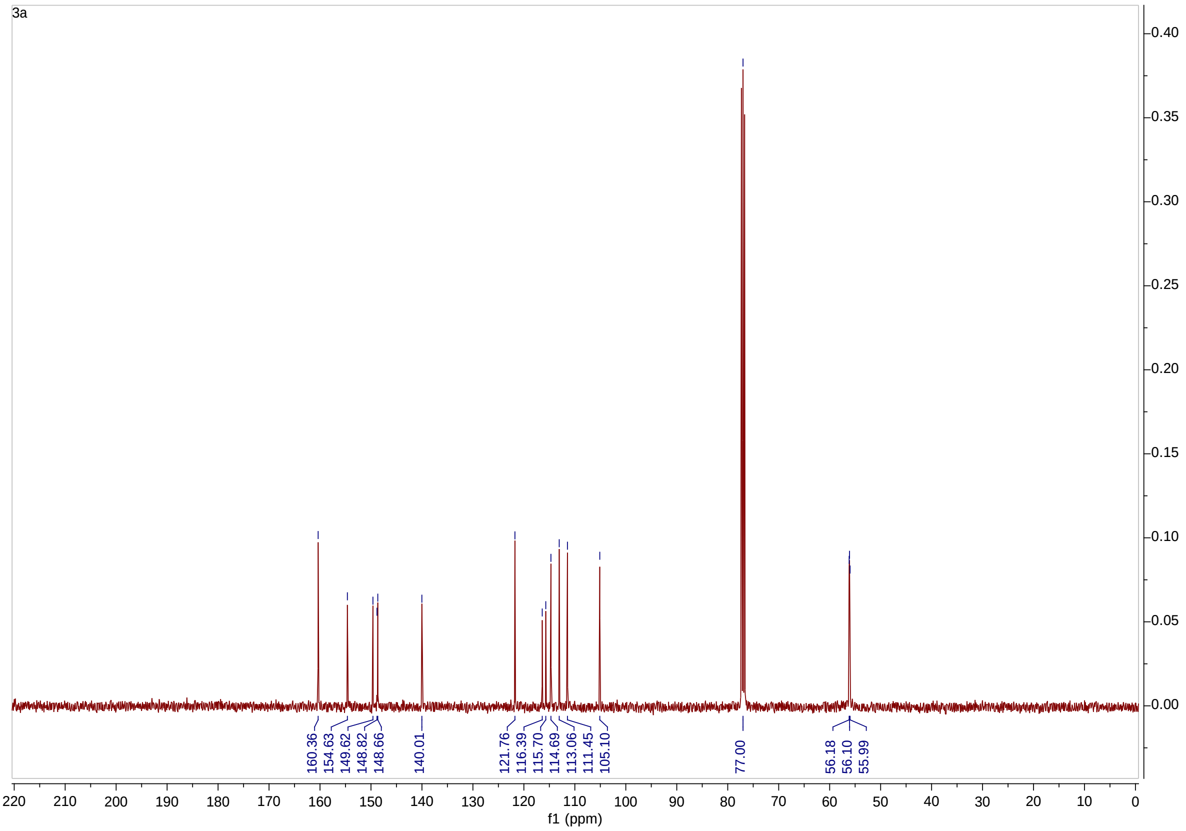

Compound 3c ($CDCl_3$)

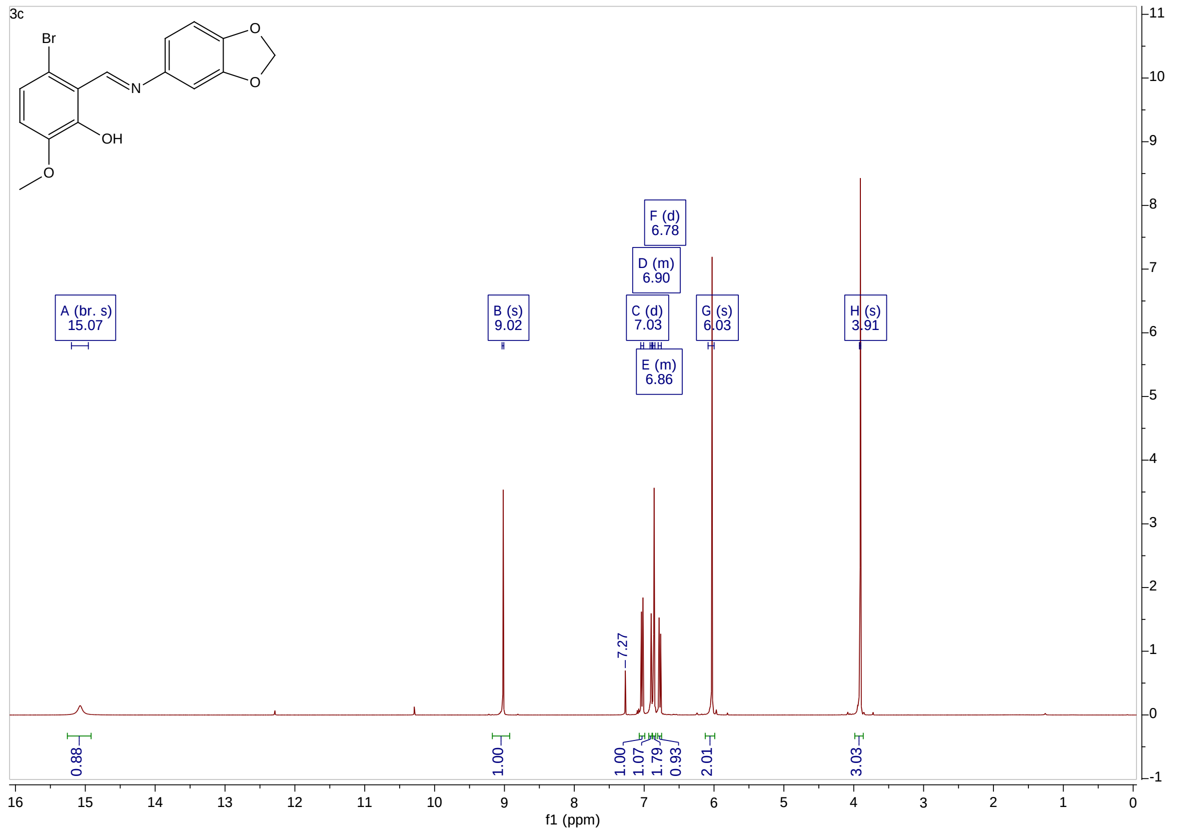

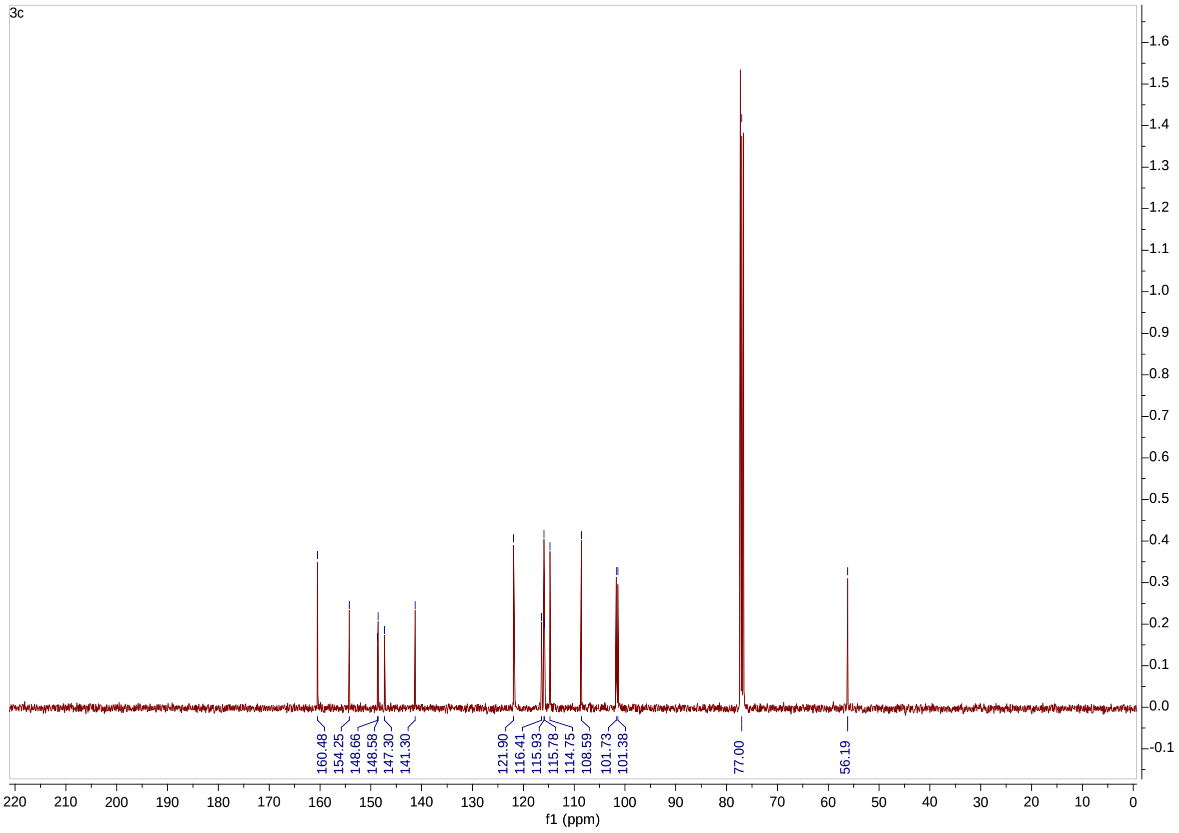

Compound 3d ($CDCl_3$)

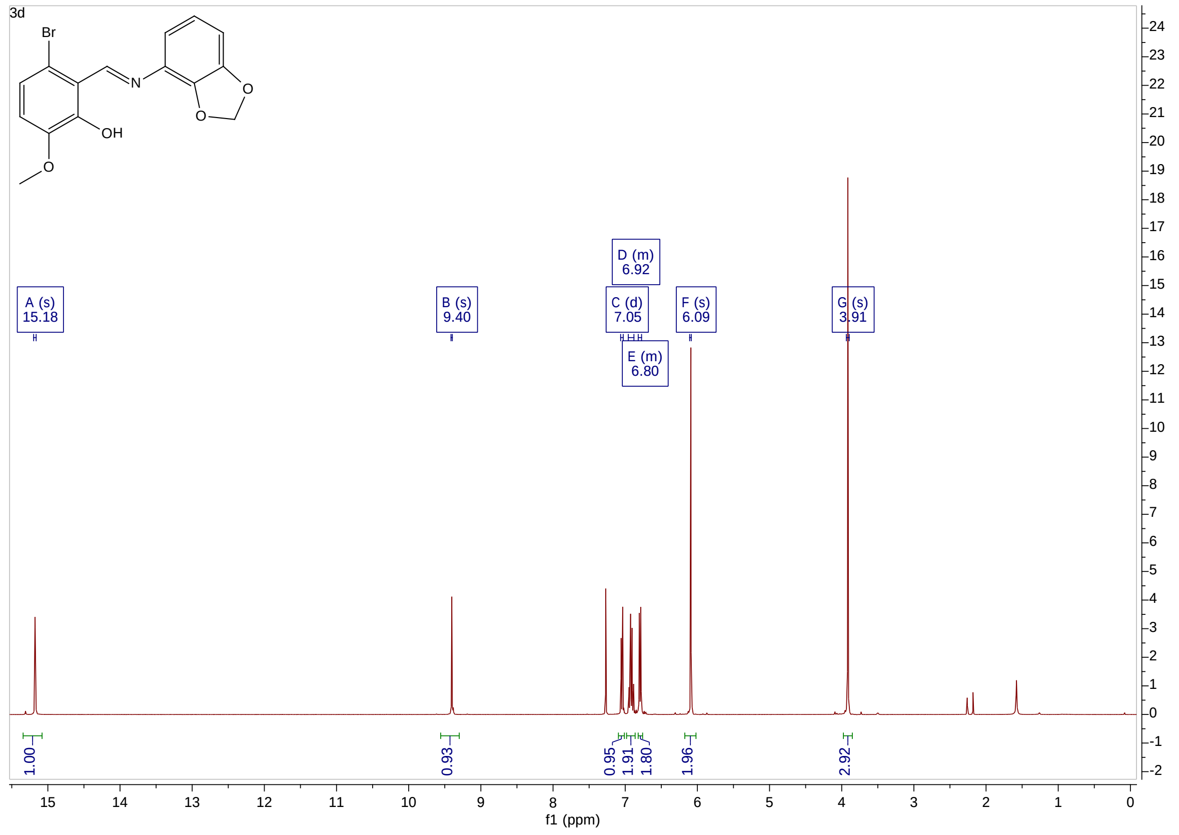

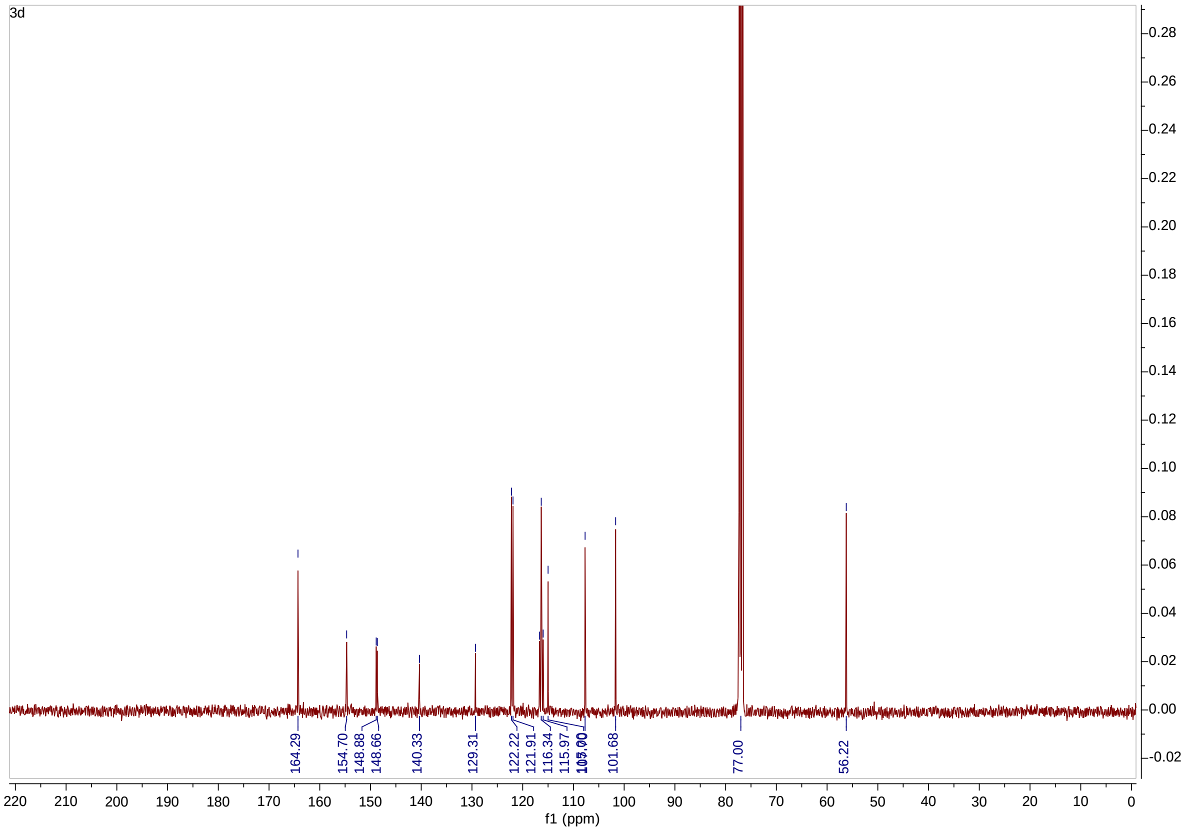

Compound 4a ($CDCl_3$)

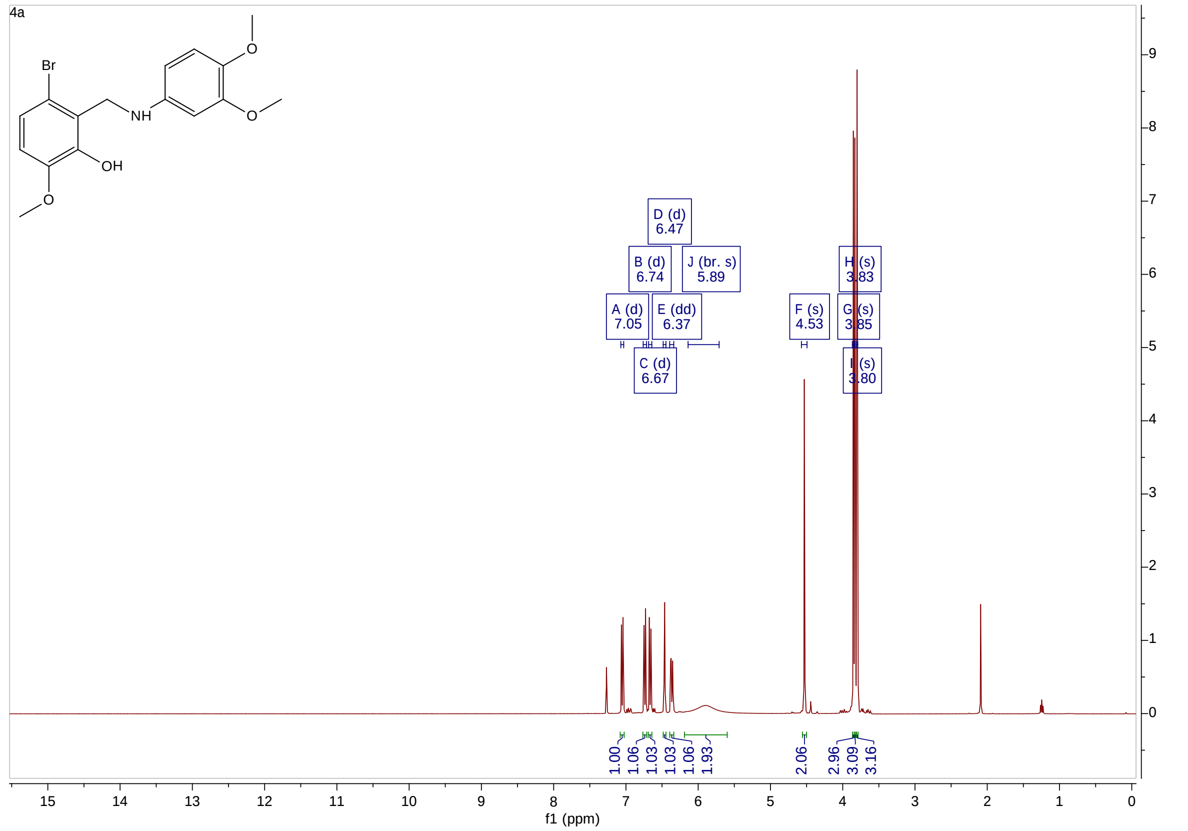

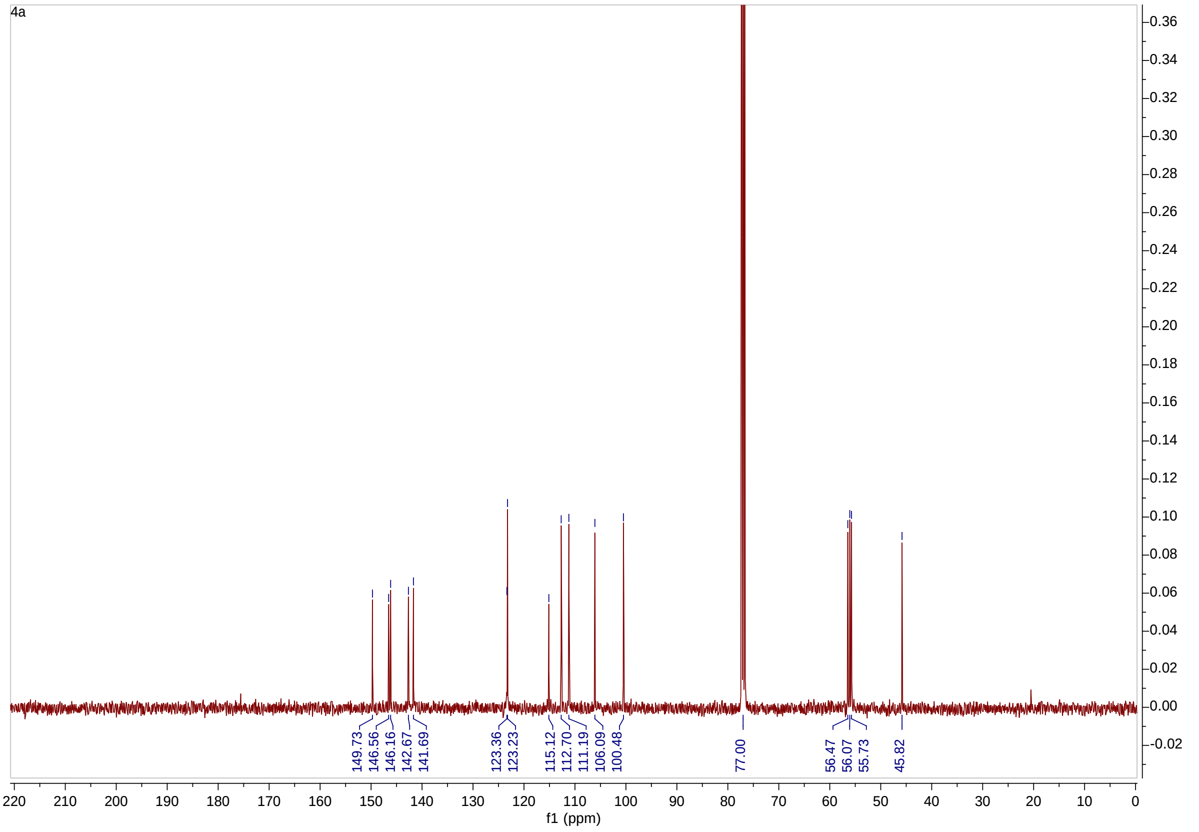

Compound 4c (DMSO-d_6)

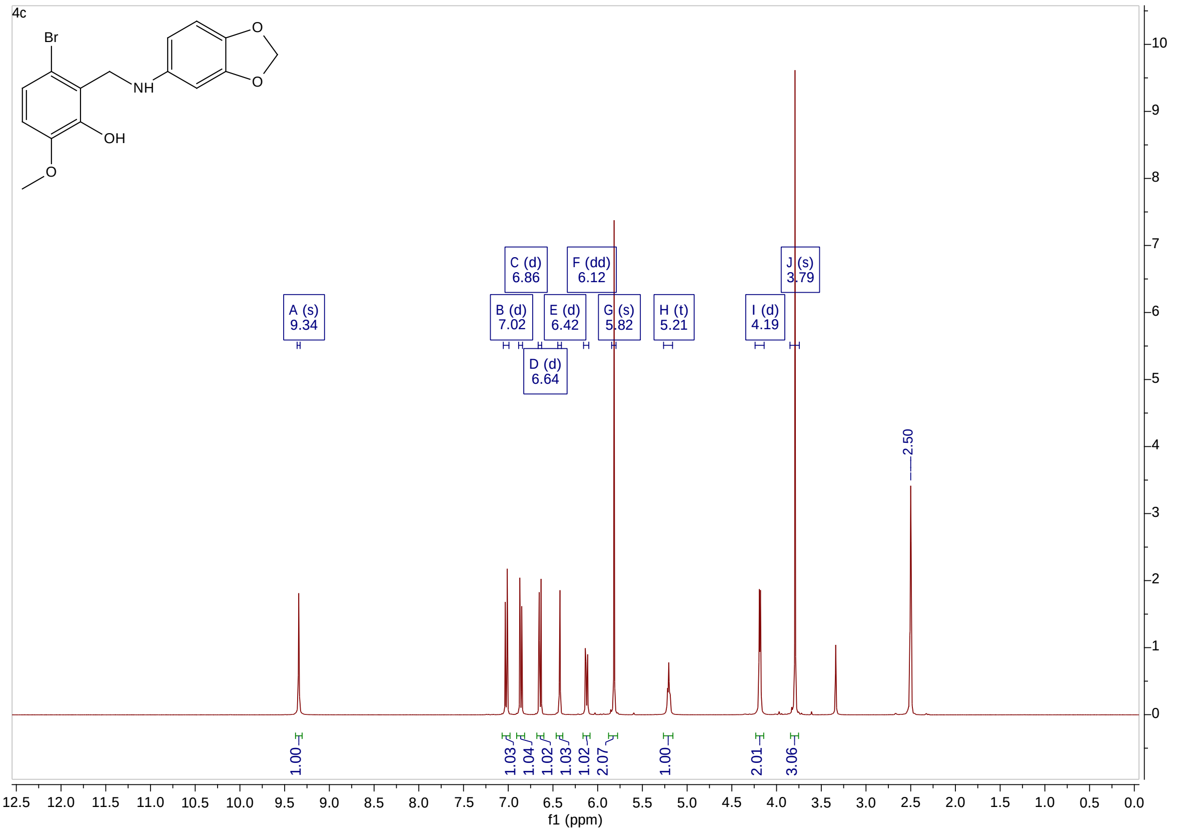

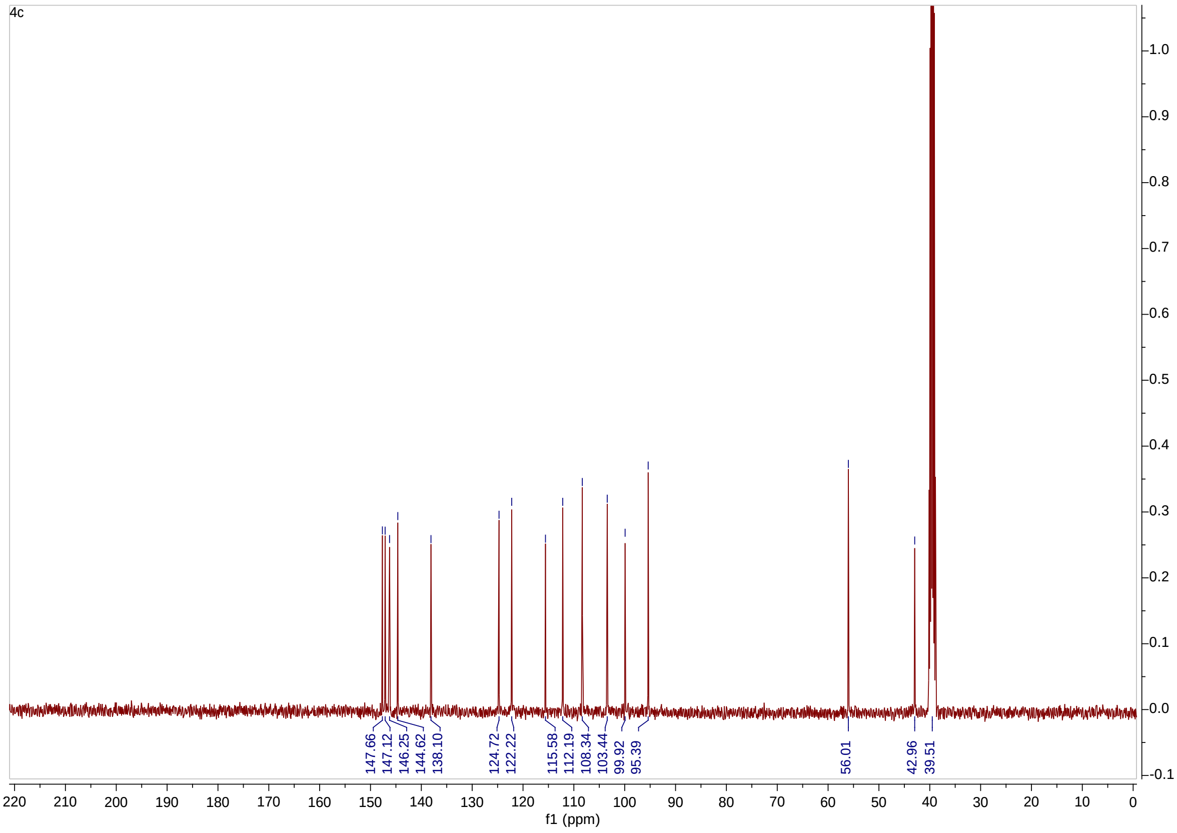

Compound 4d ($CDCl_3$)

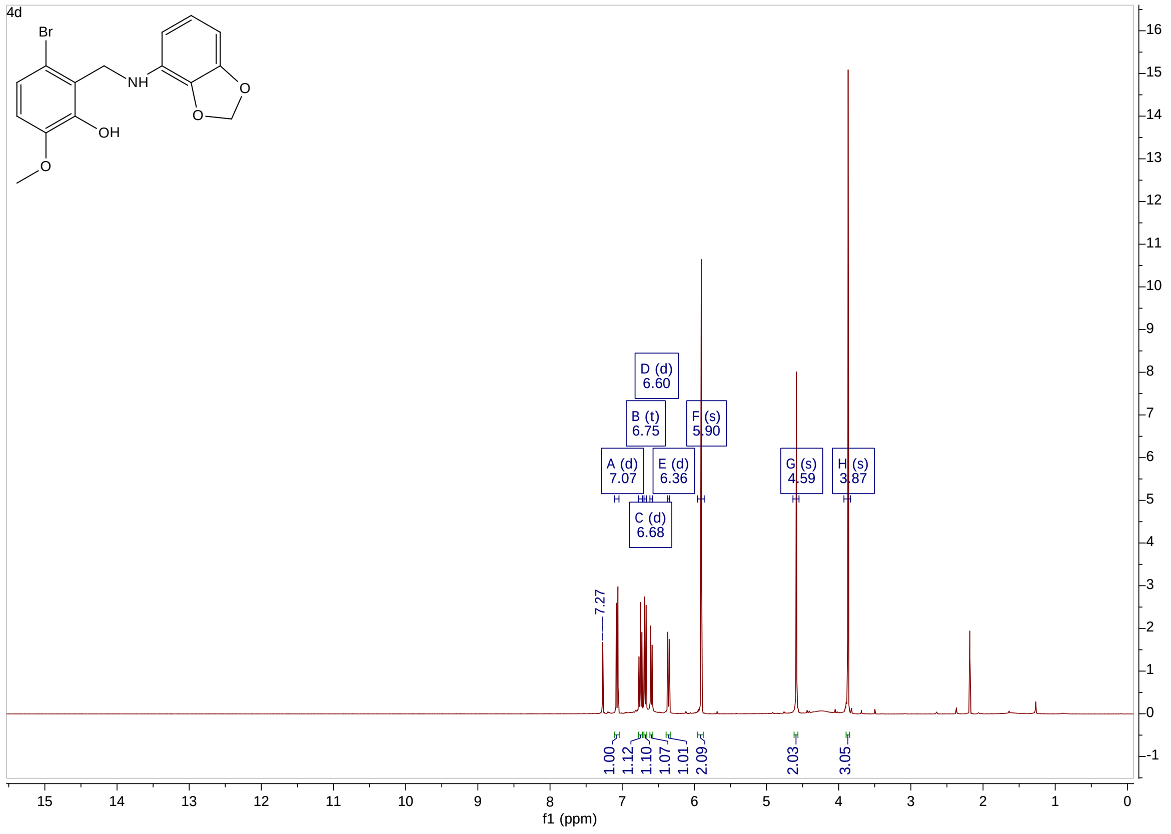

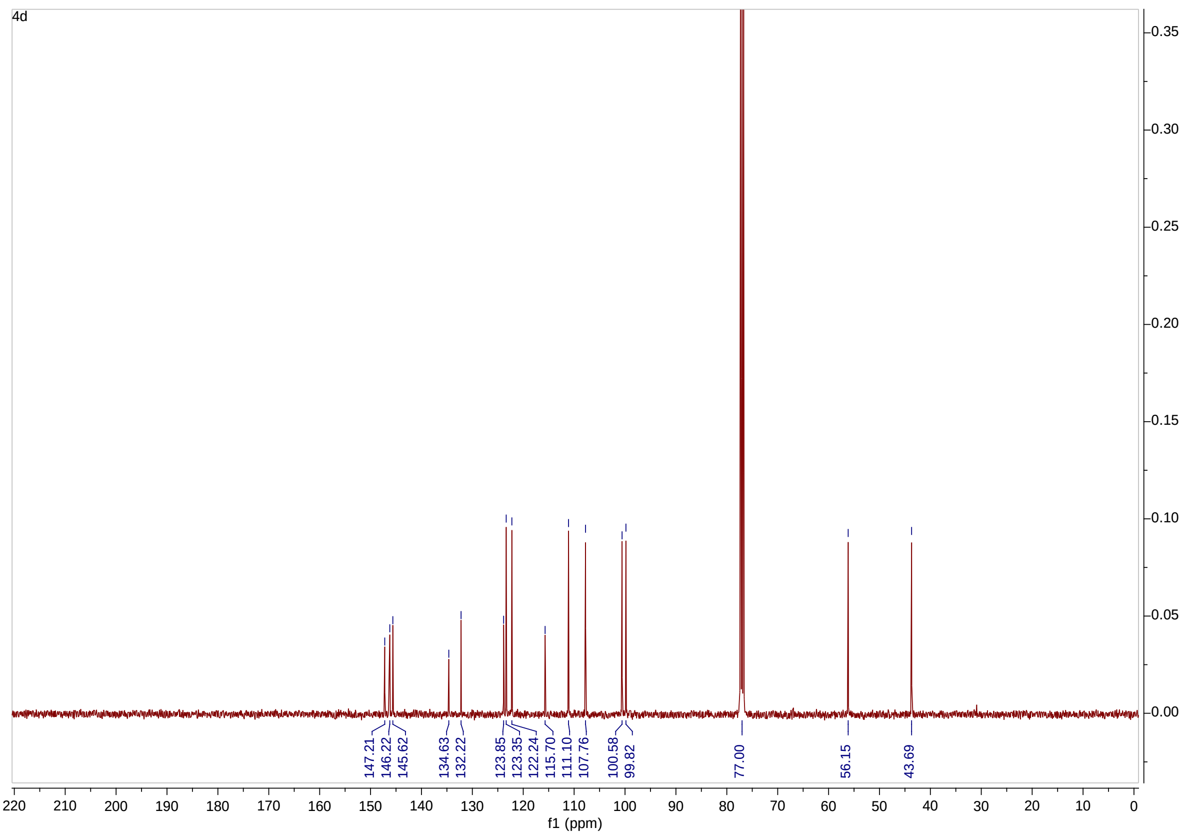

Compound 4e ($CDCl_3$)

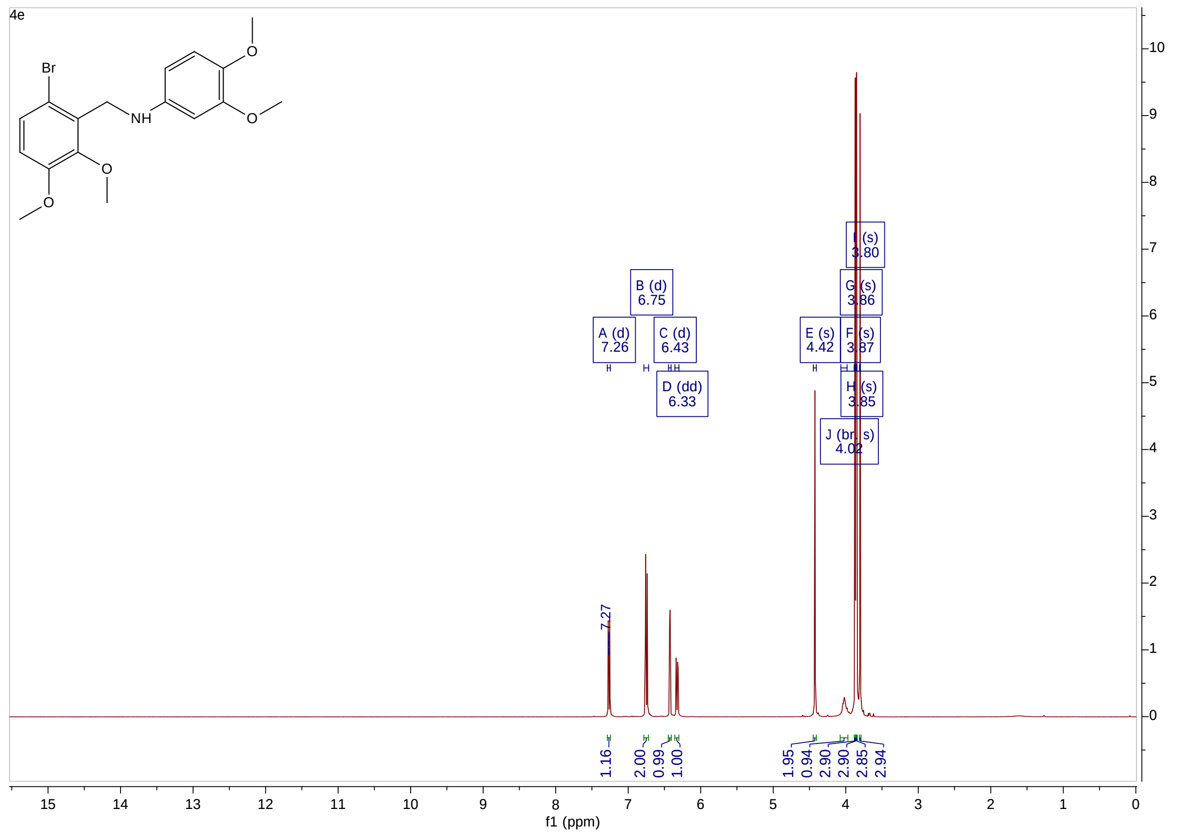

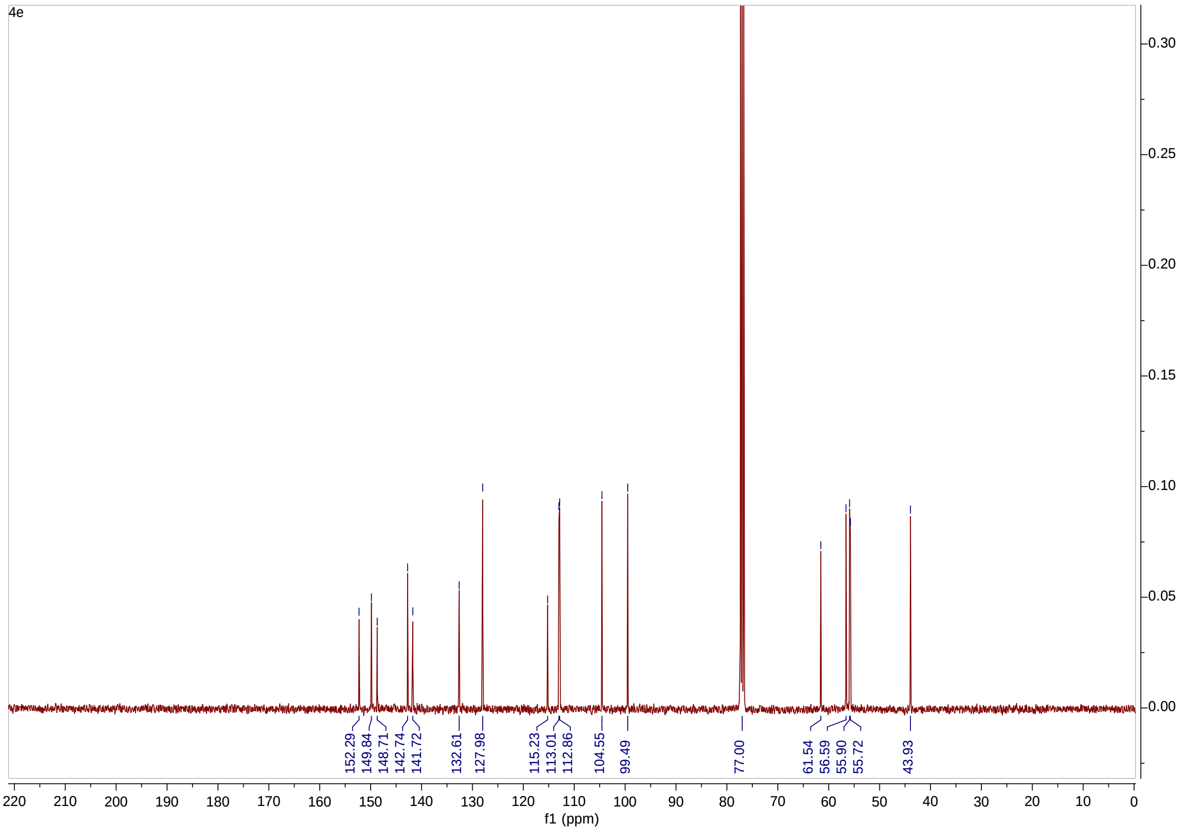

Compound 4f (DMSO-d_6)

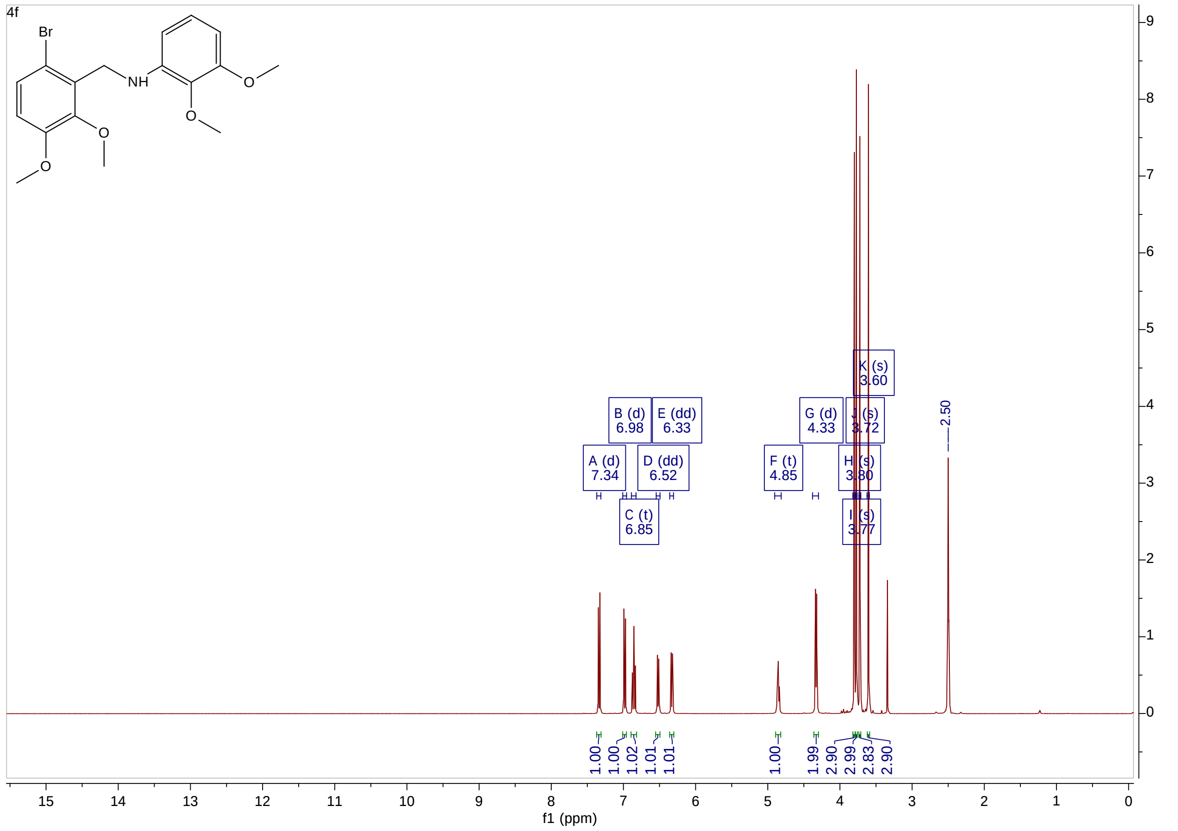

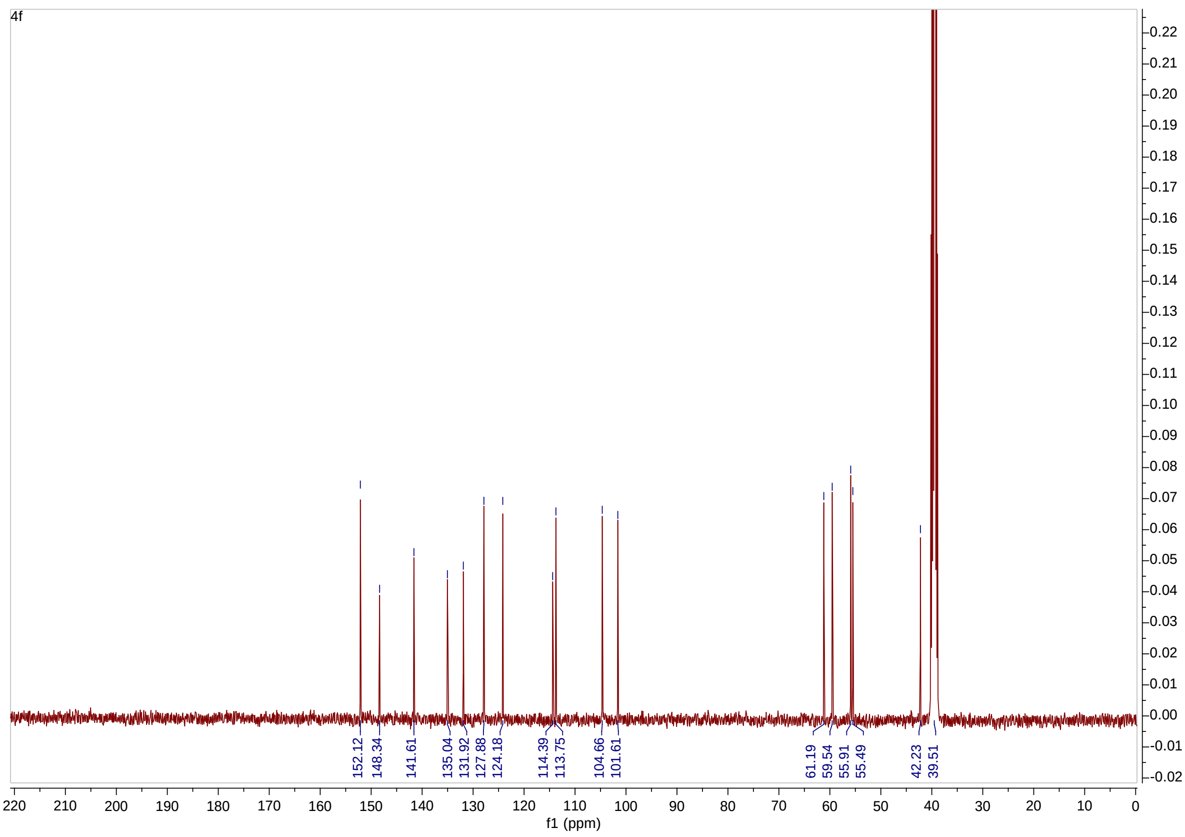

Compound 4g (DMSO-d_6)

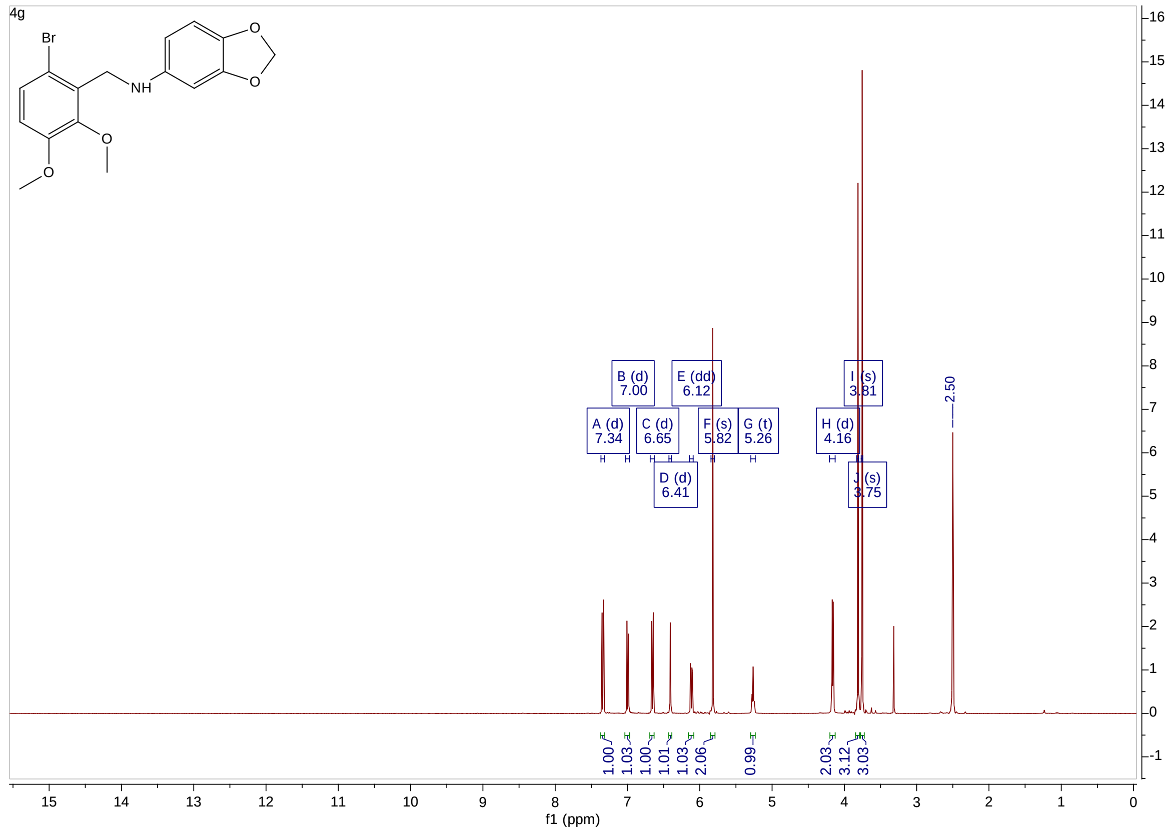

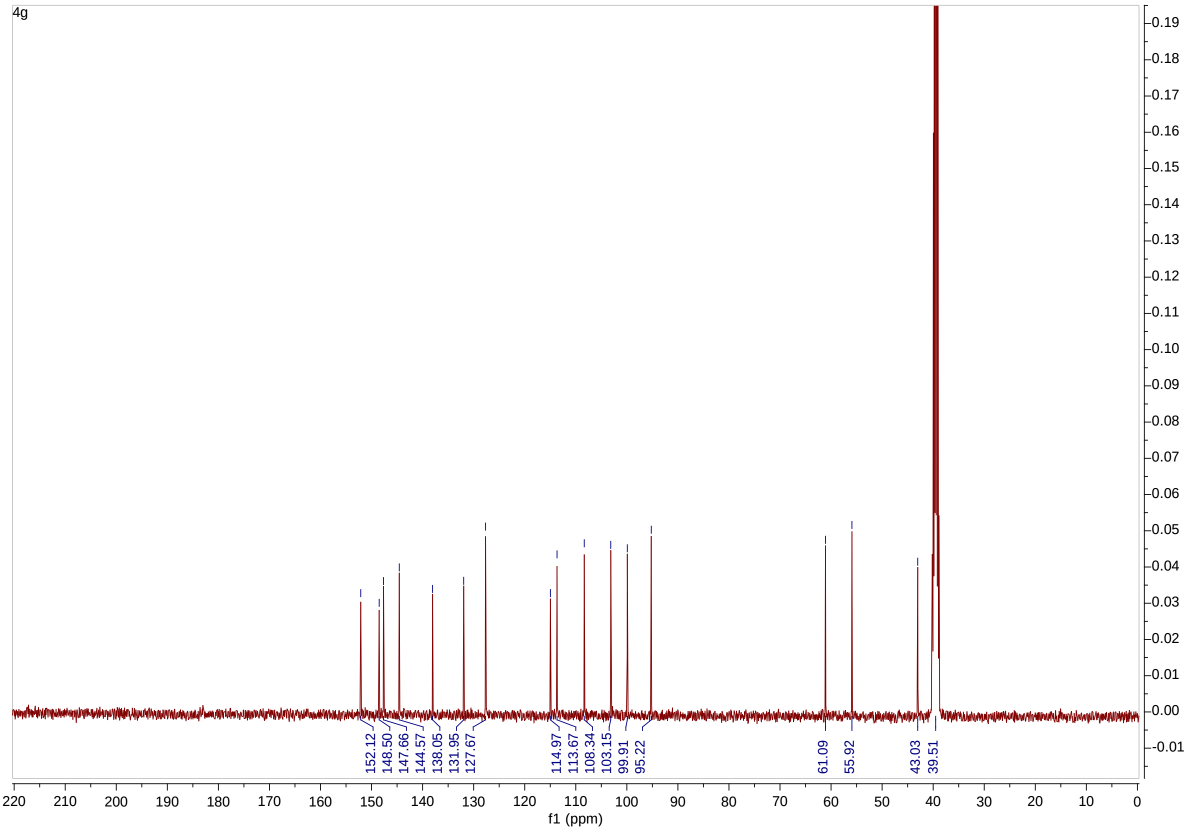

Compound 4h (DMSO-d_6)

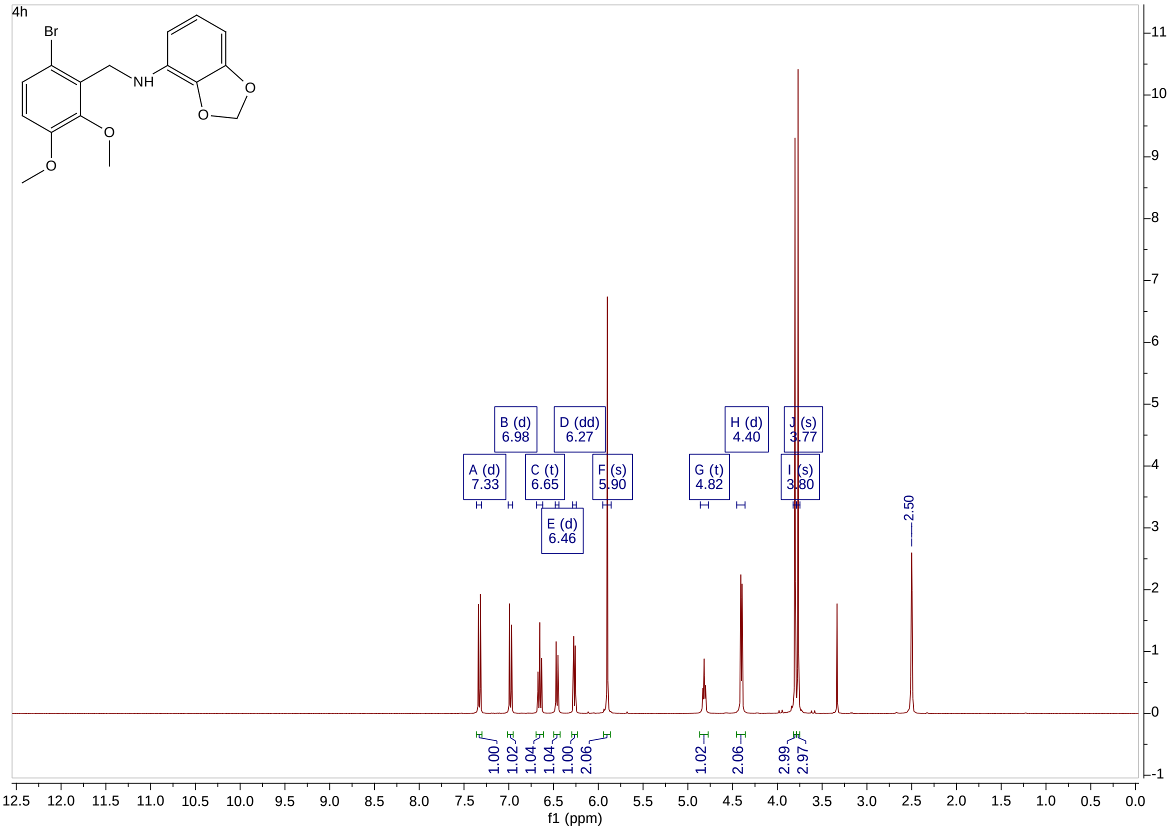

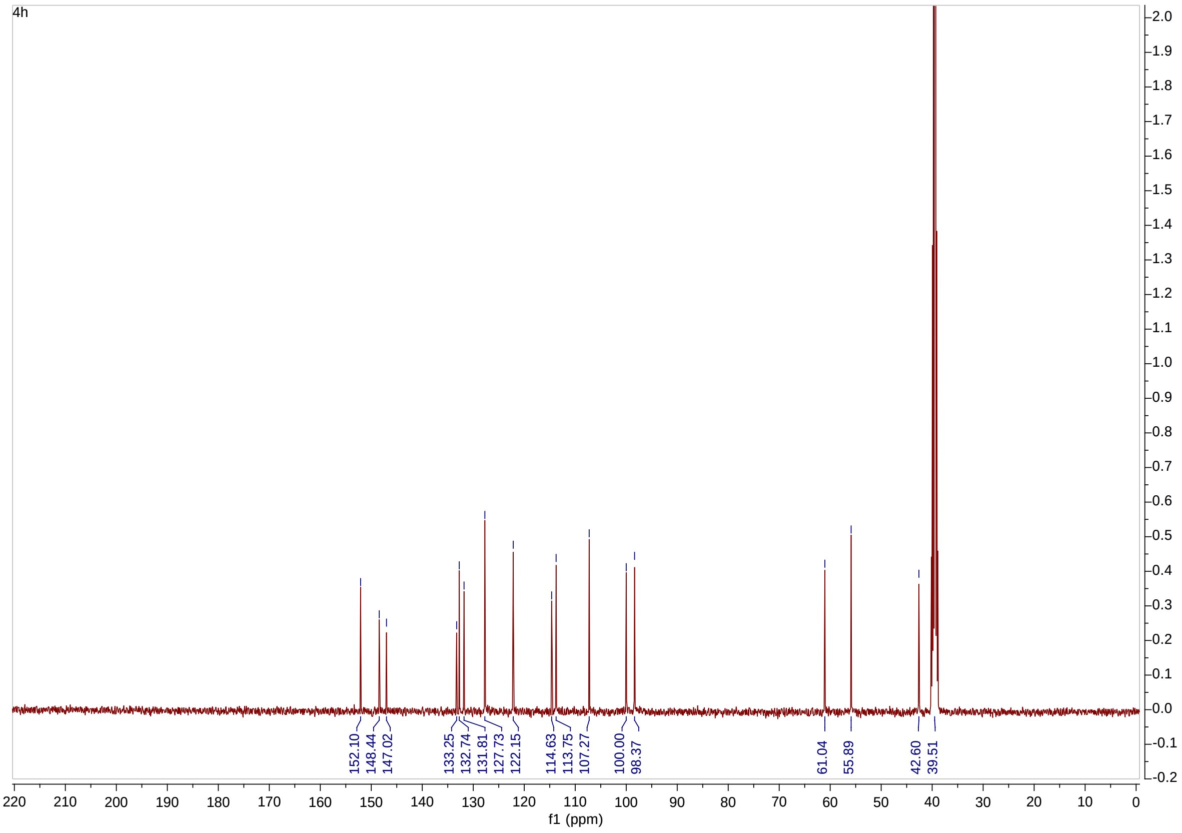

Compound 4i (DMSO-d_6)

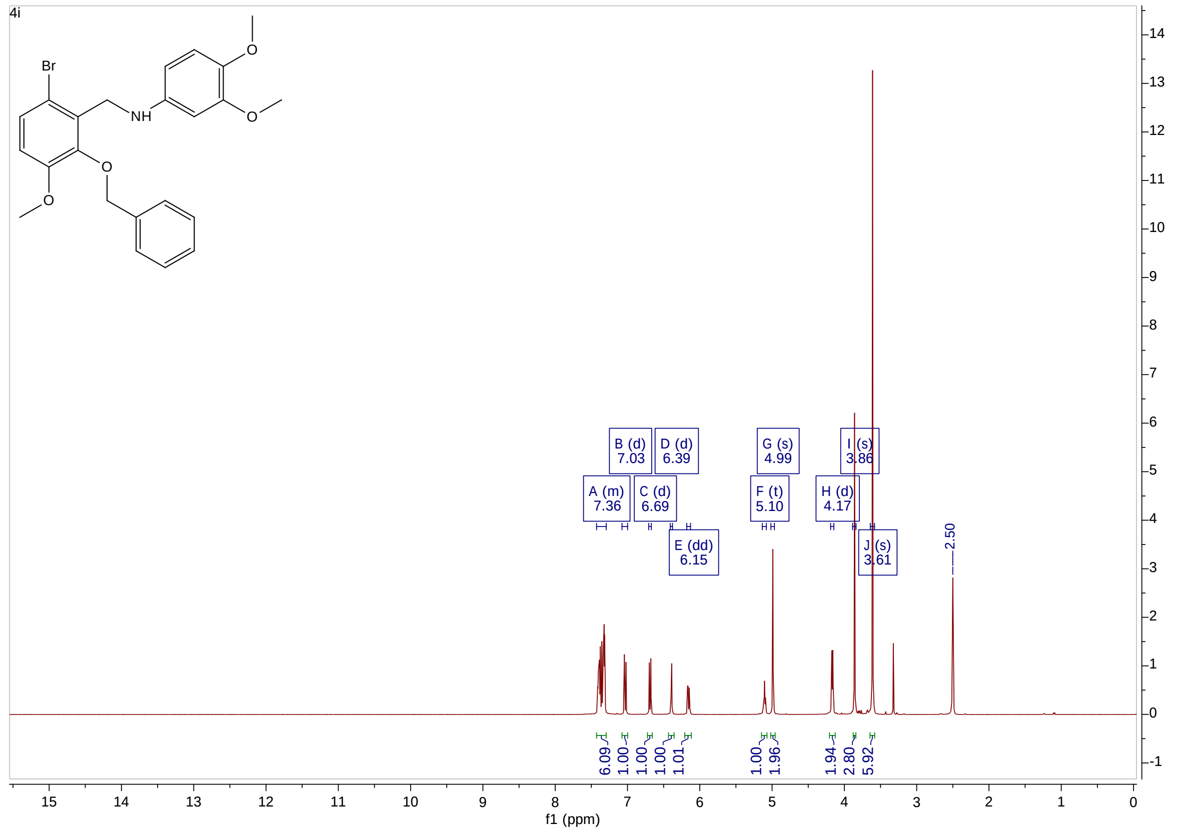

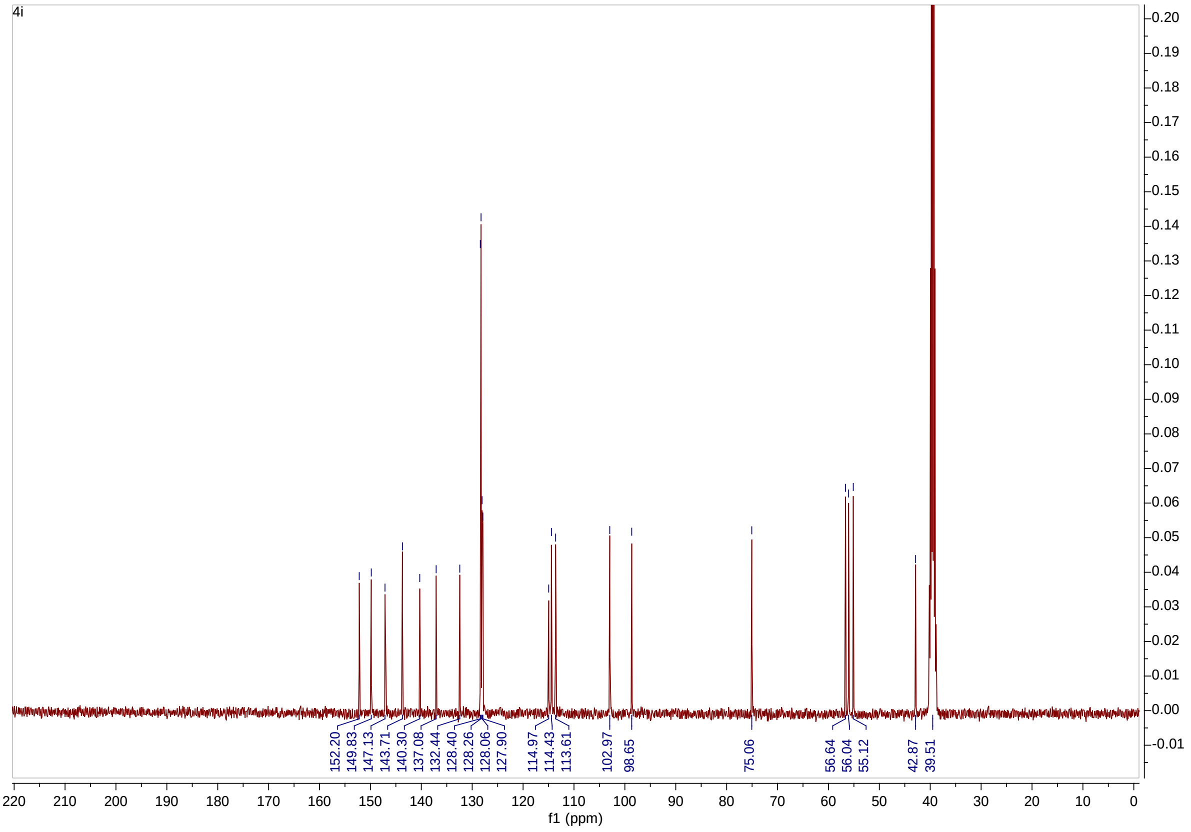

Compound 4j (DMSO-d_6)

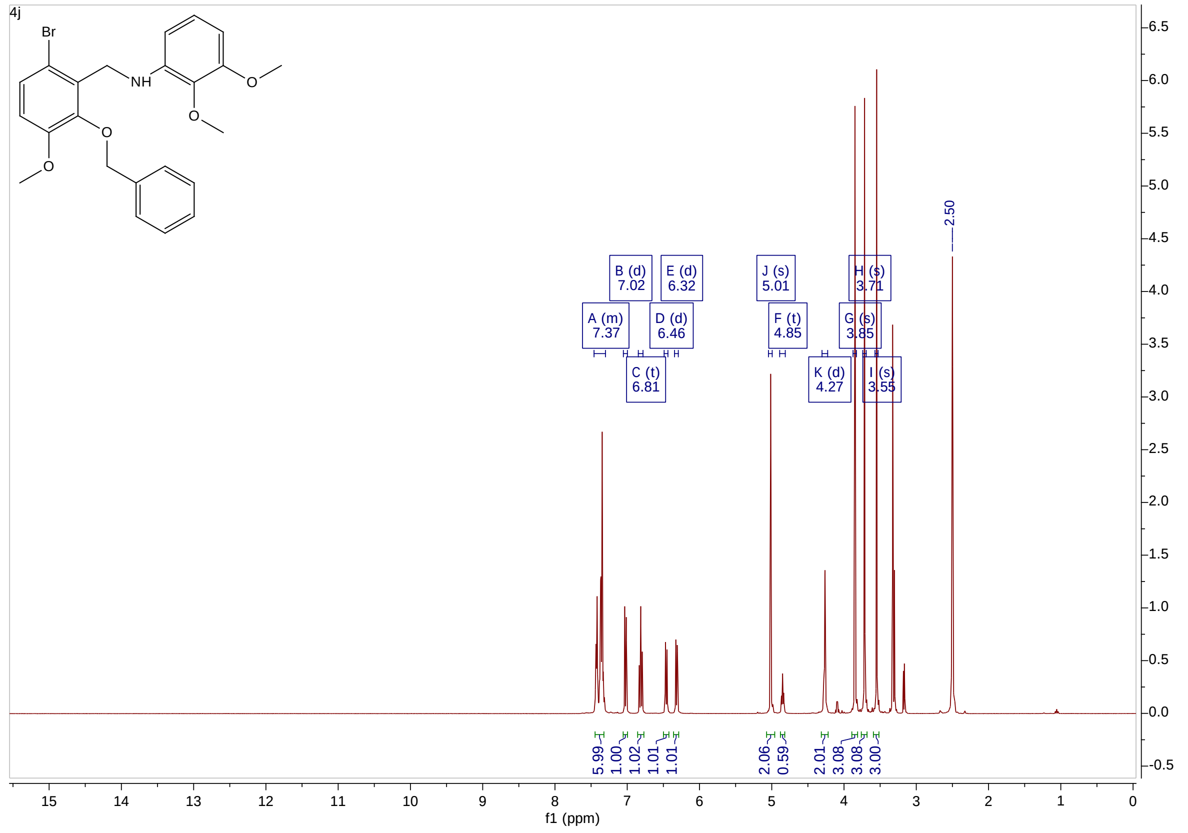

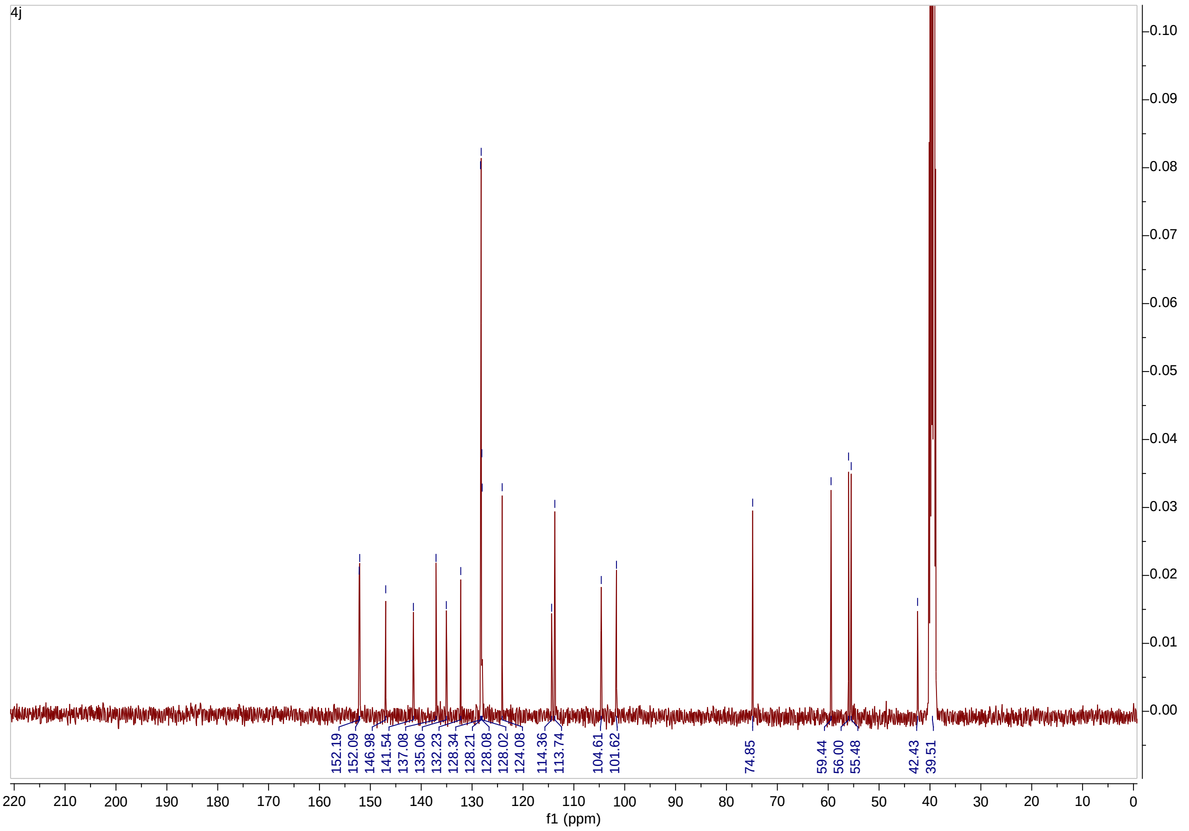

Compound 4k (DMSO-d_6)

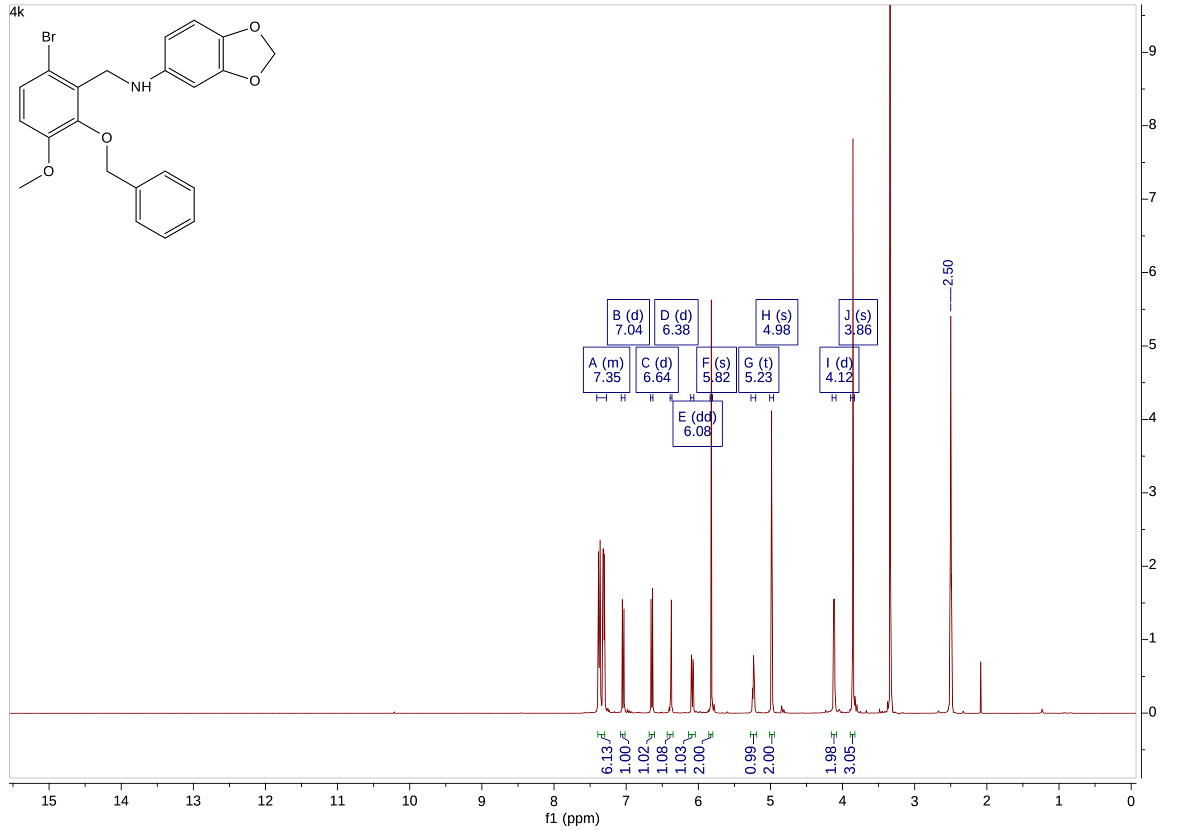

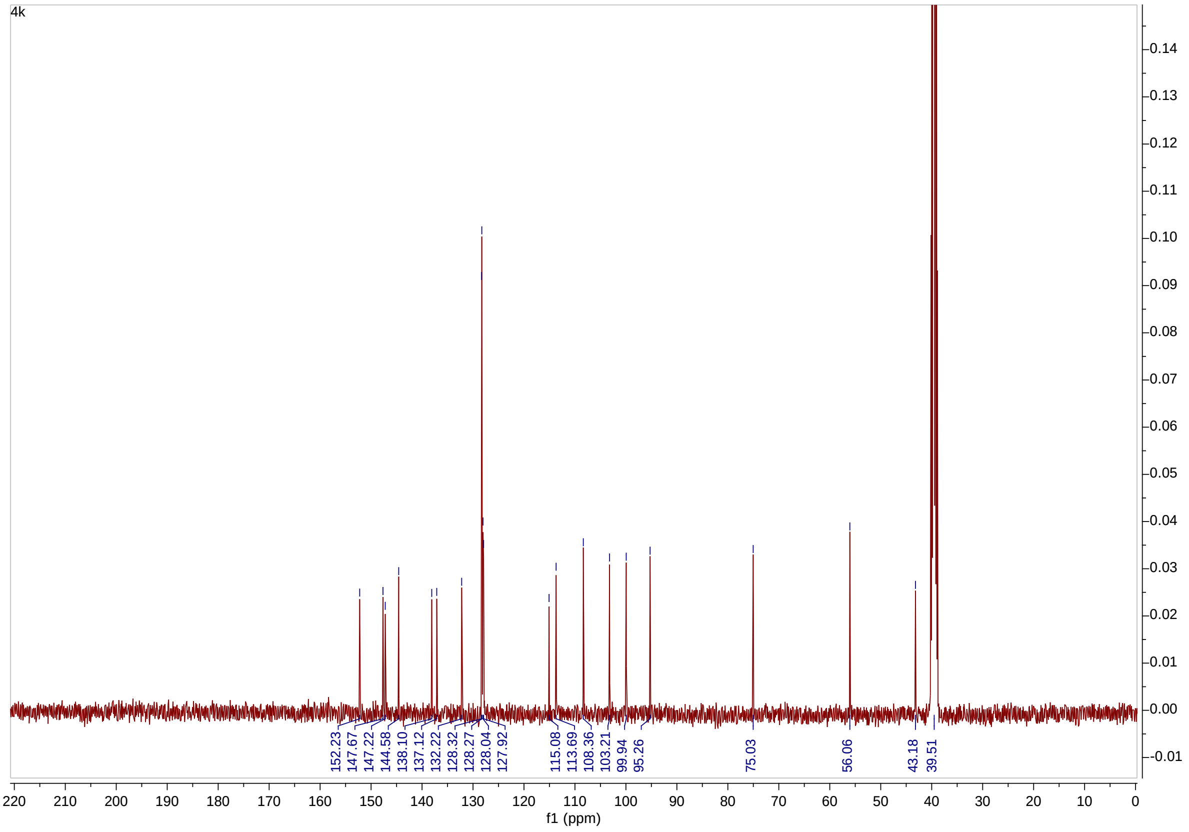

Compound 4l (DMSO-d_6)

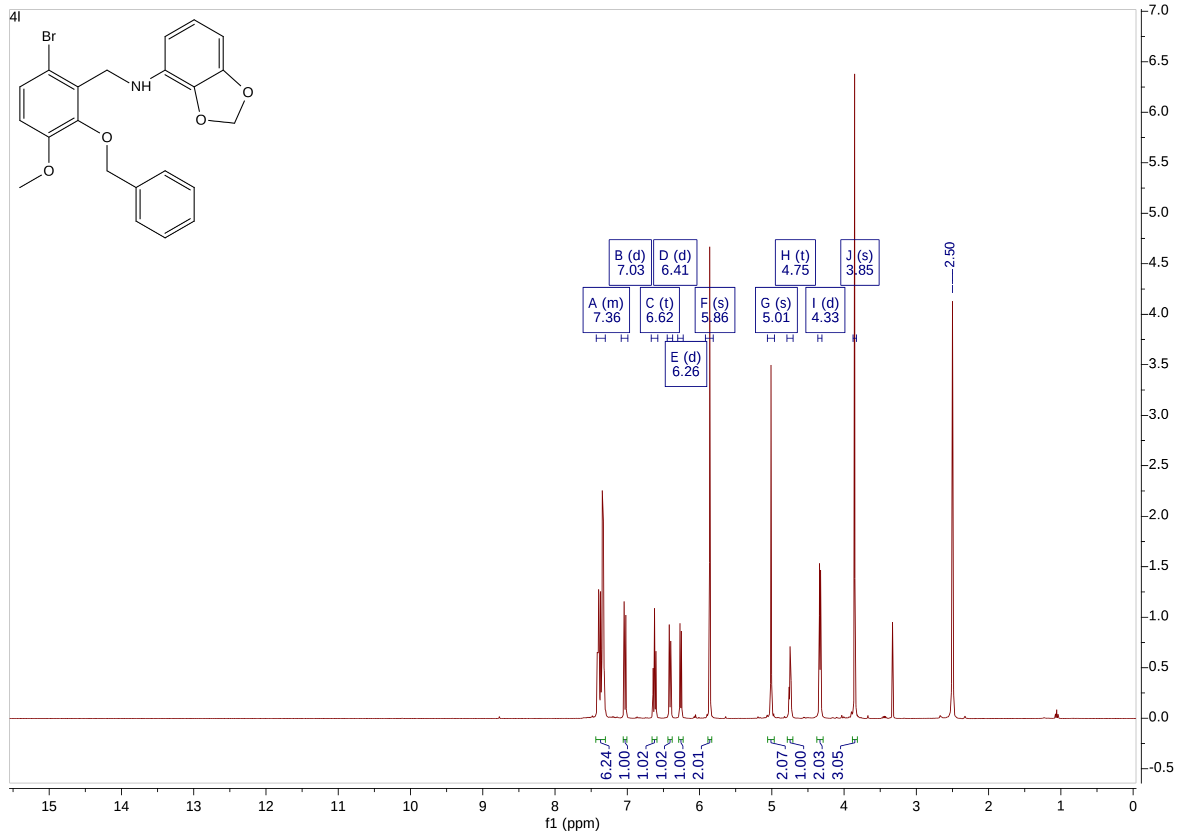

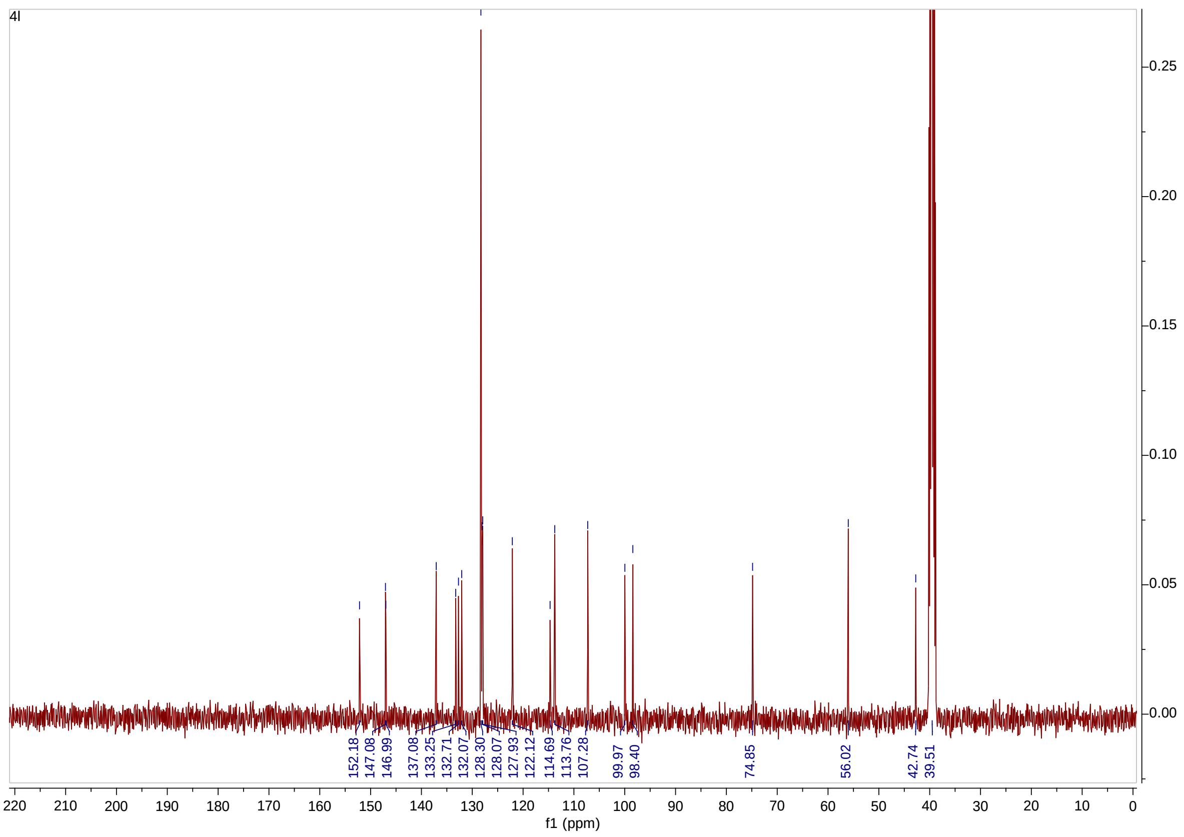

Compound 5a (DMSO-d_6)

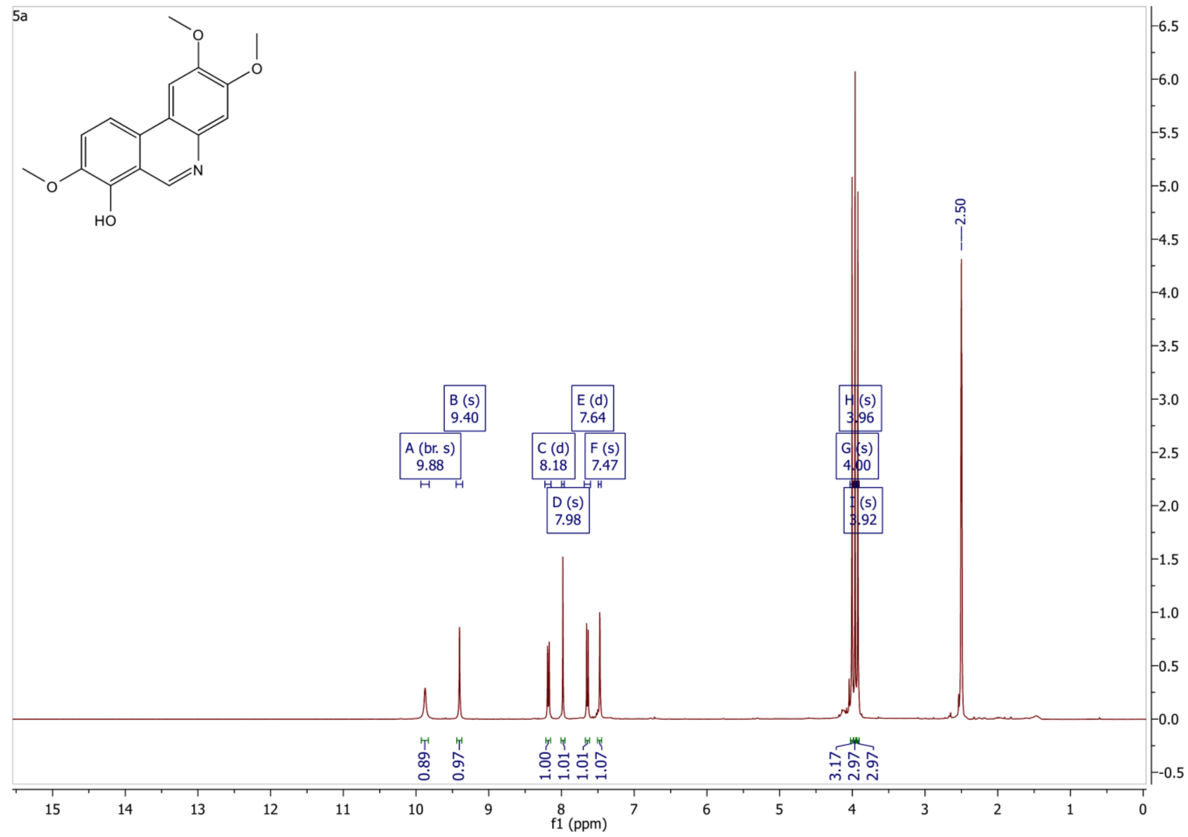

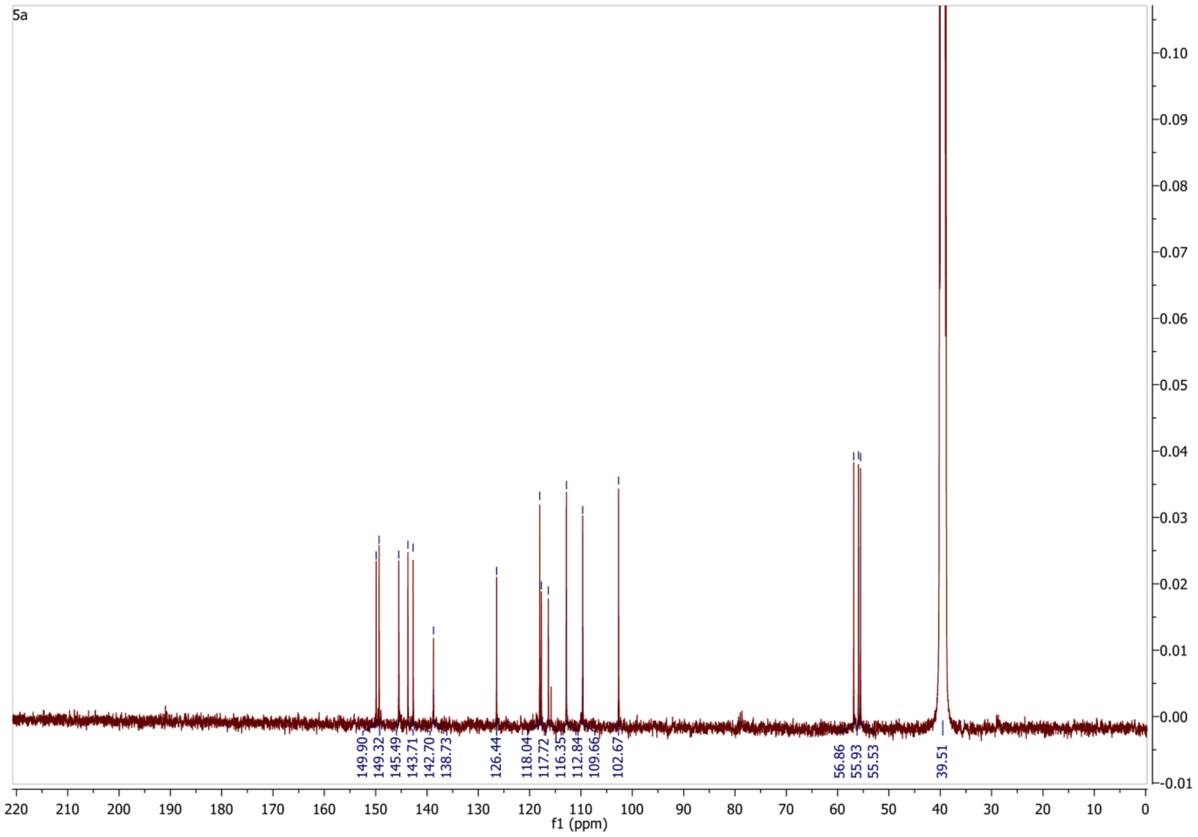

Compound 5b (DMSO-d_6)

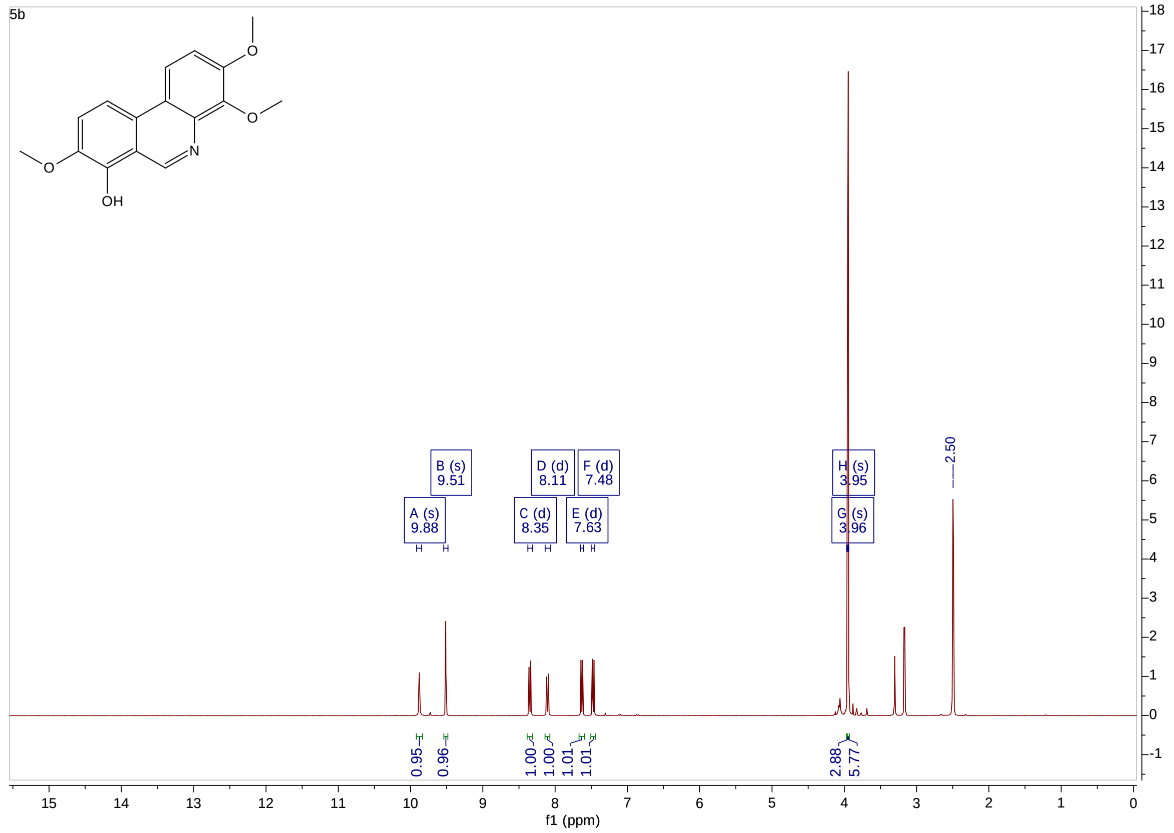

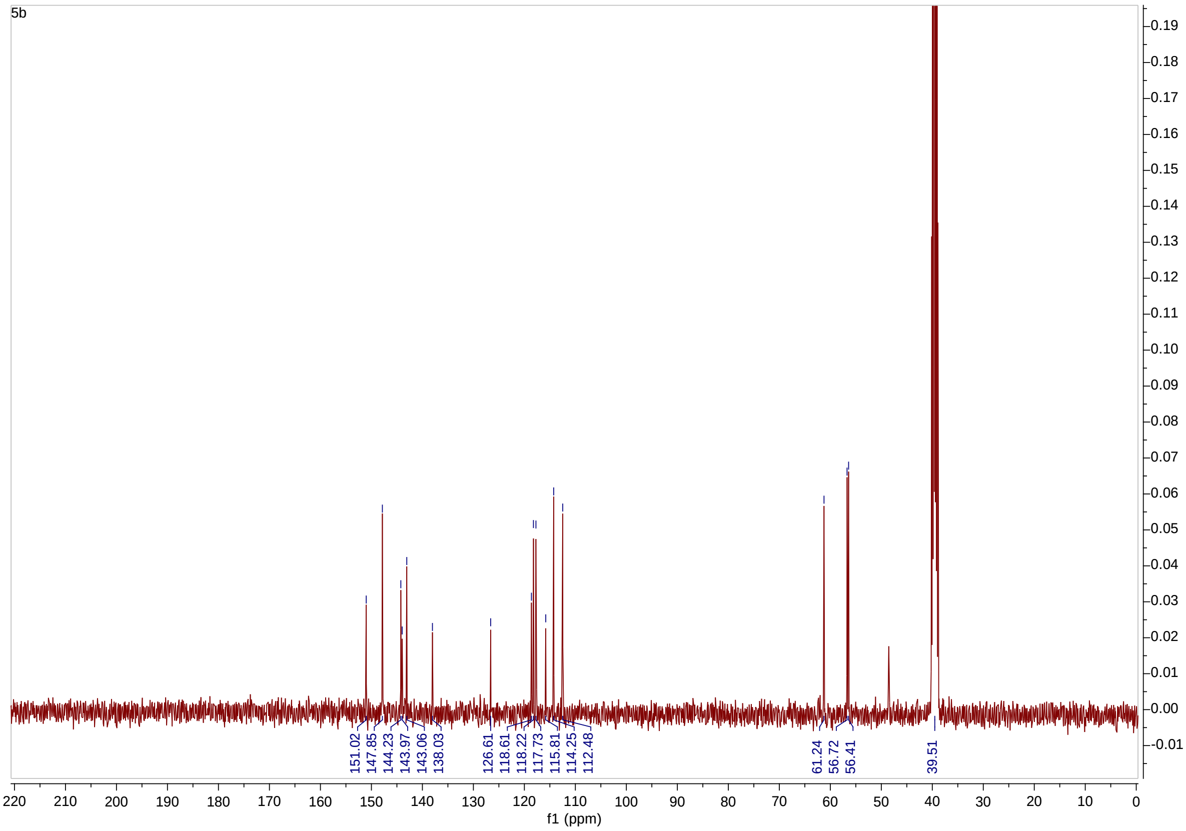

Compound 5c (DMSO-d_6)

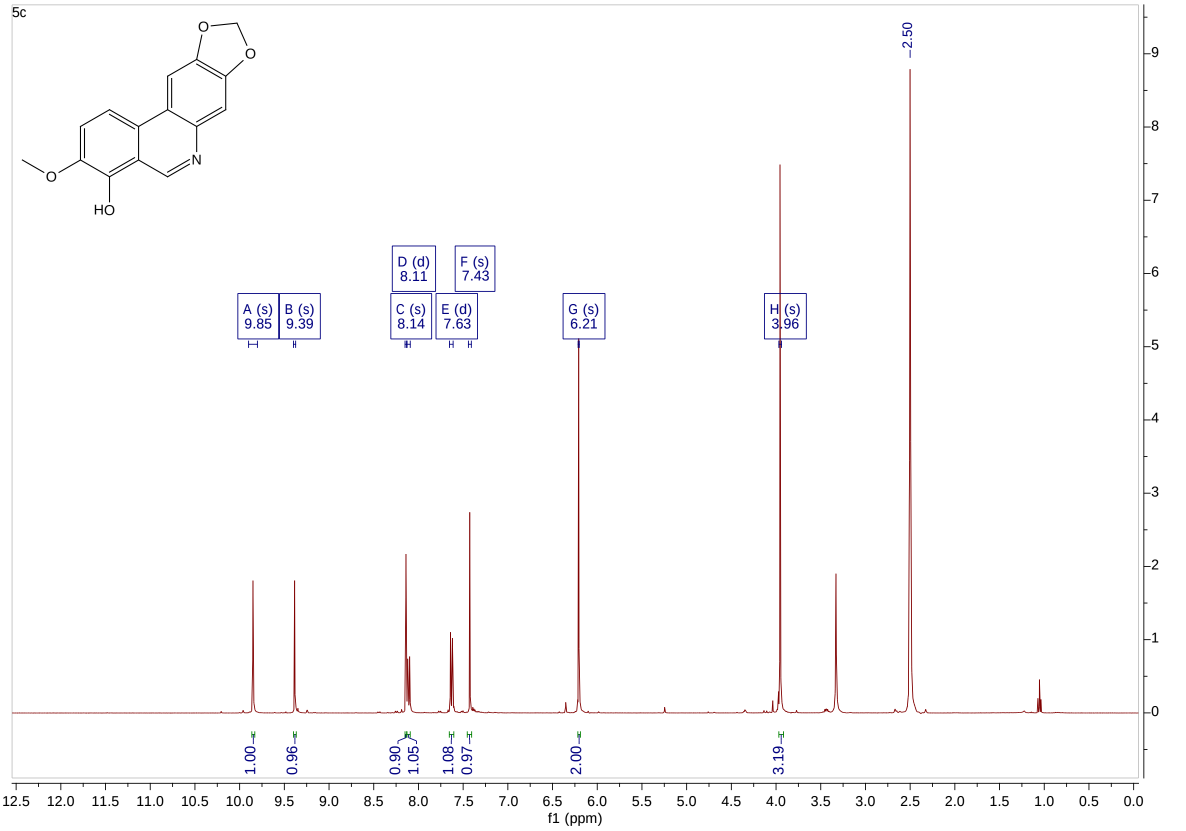

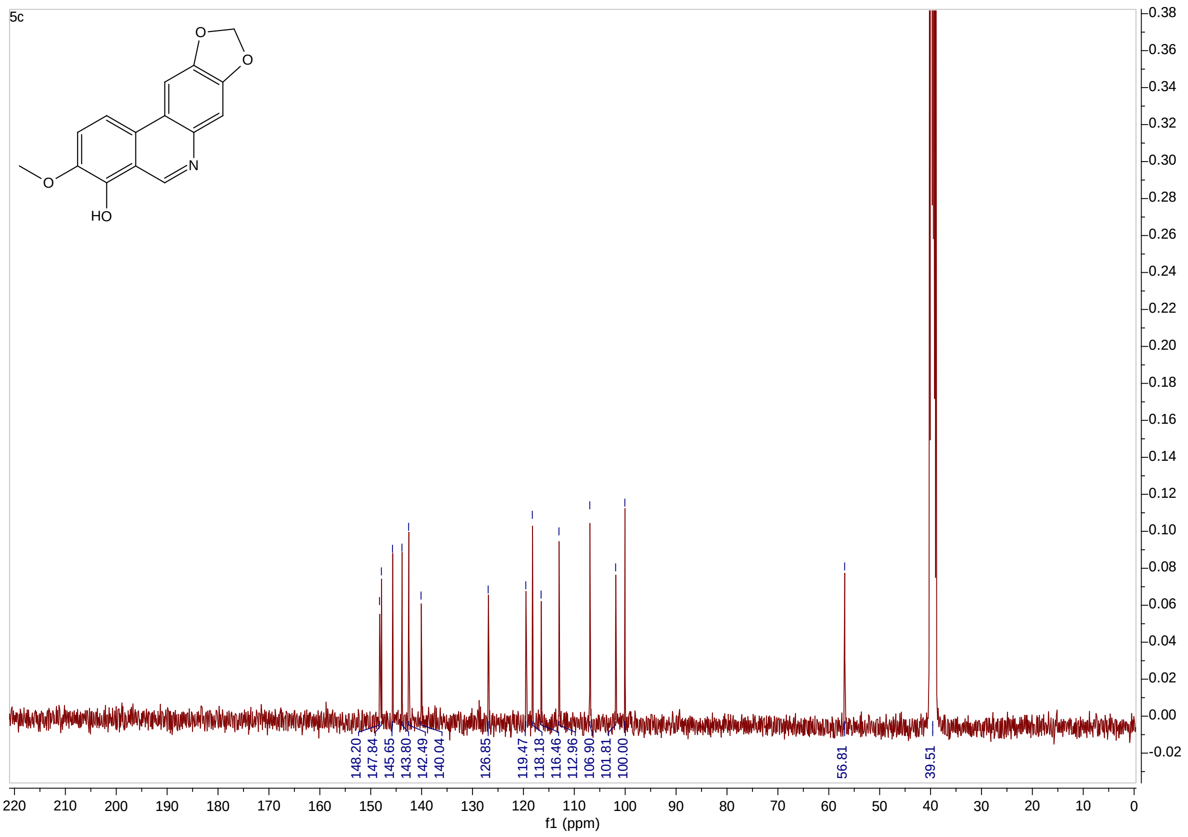

Compound 5d (DMSO-d_6)

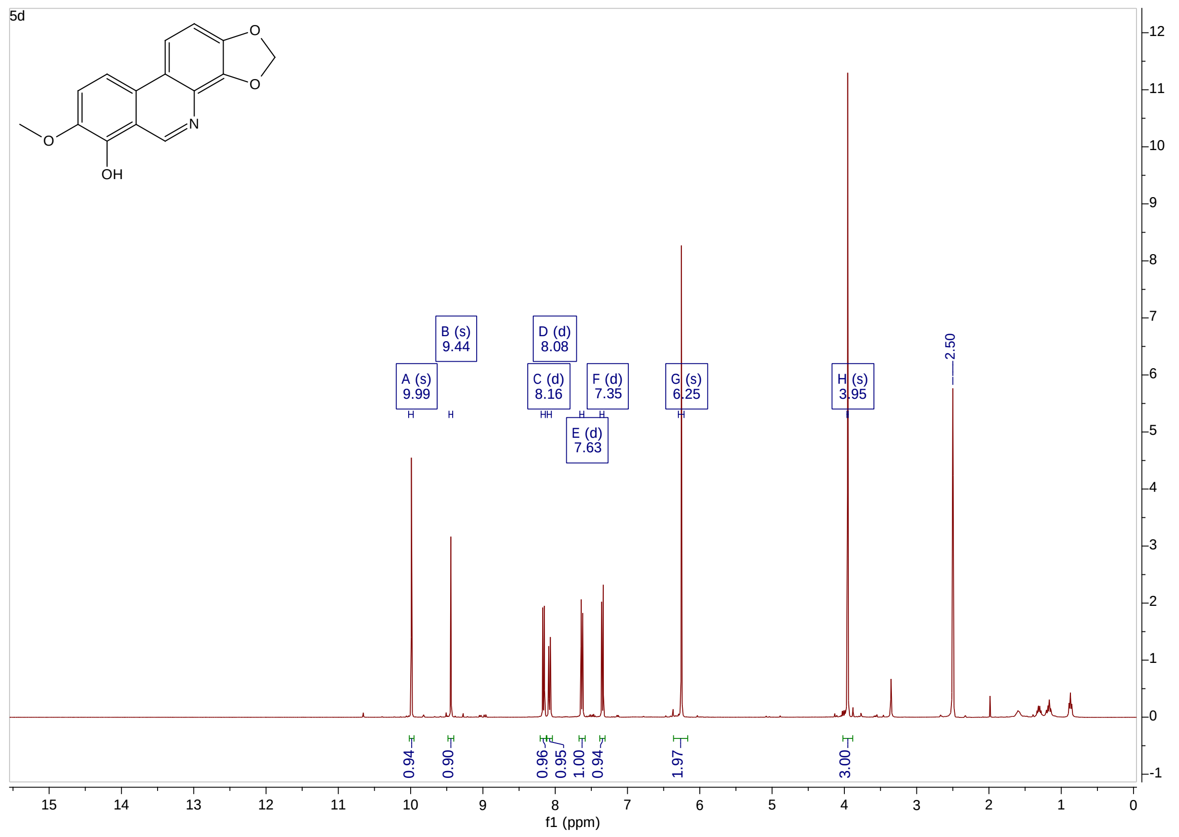

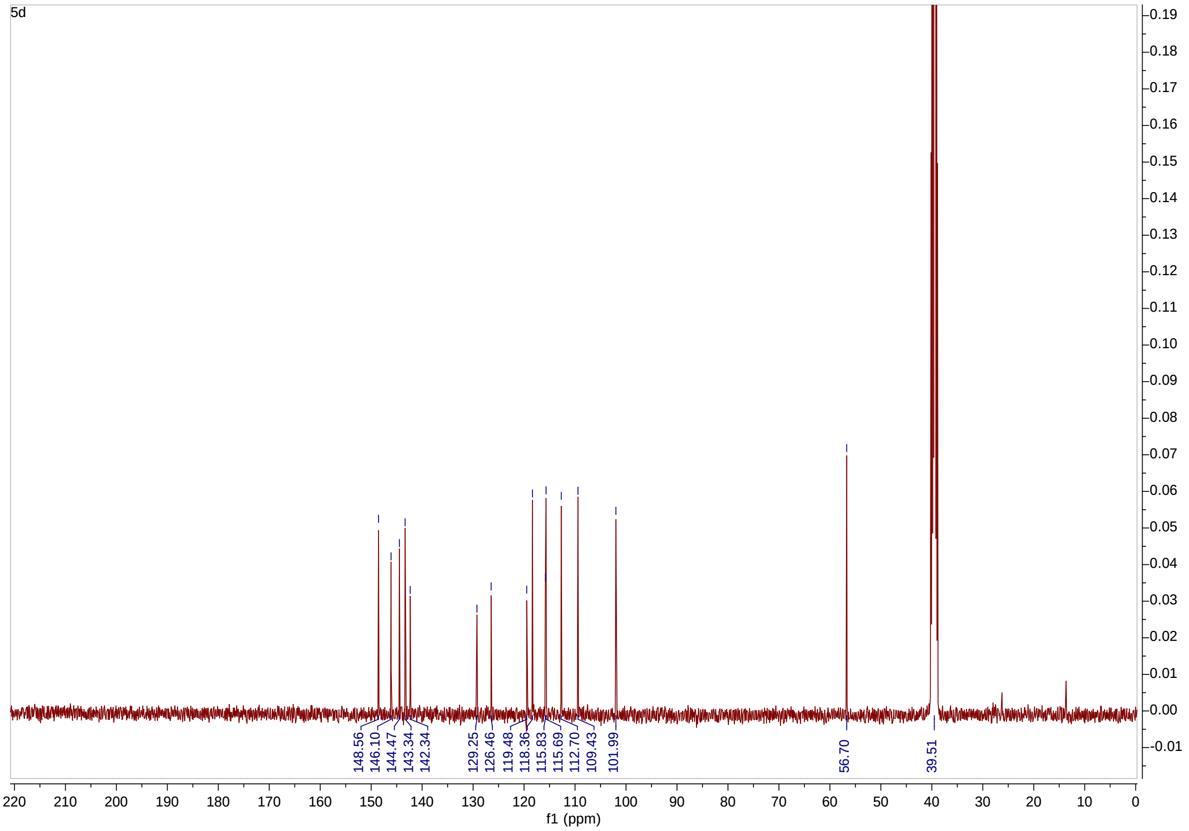

Compound 5e (DMSO-d_6)

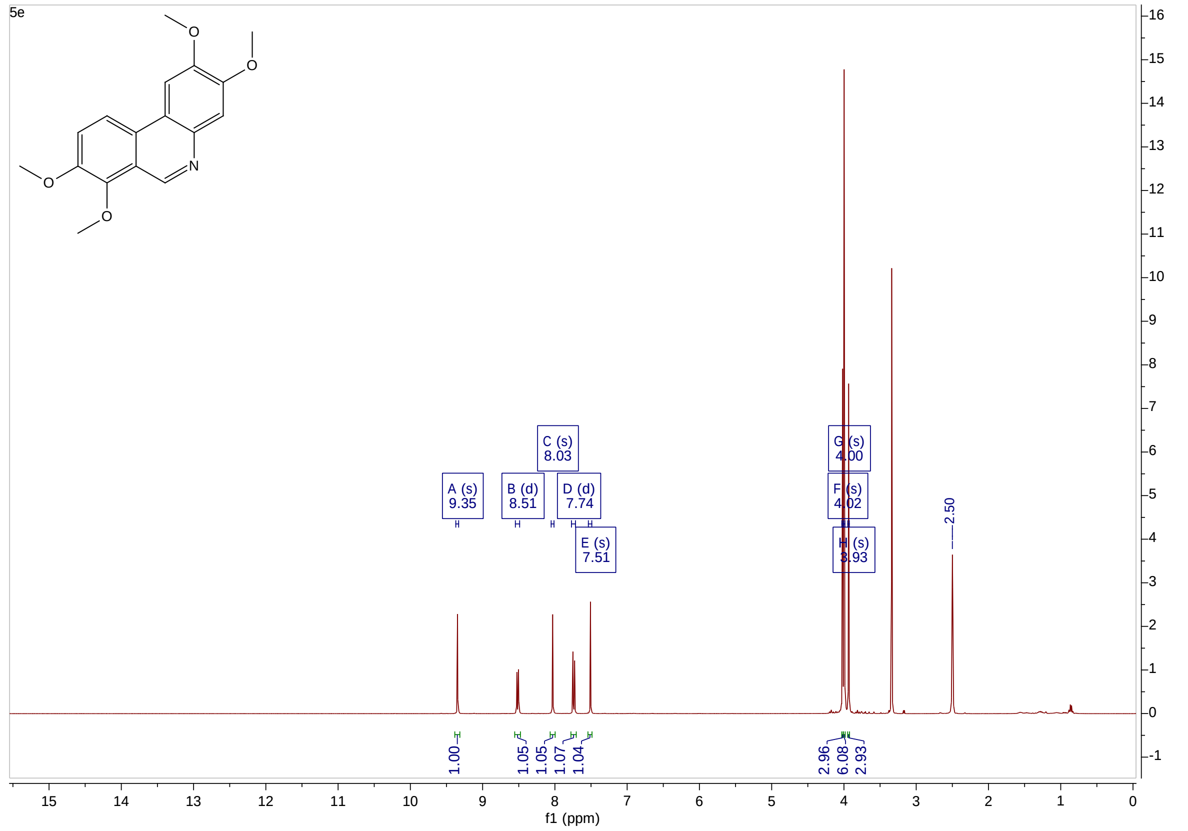

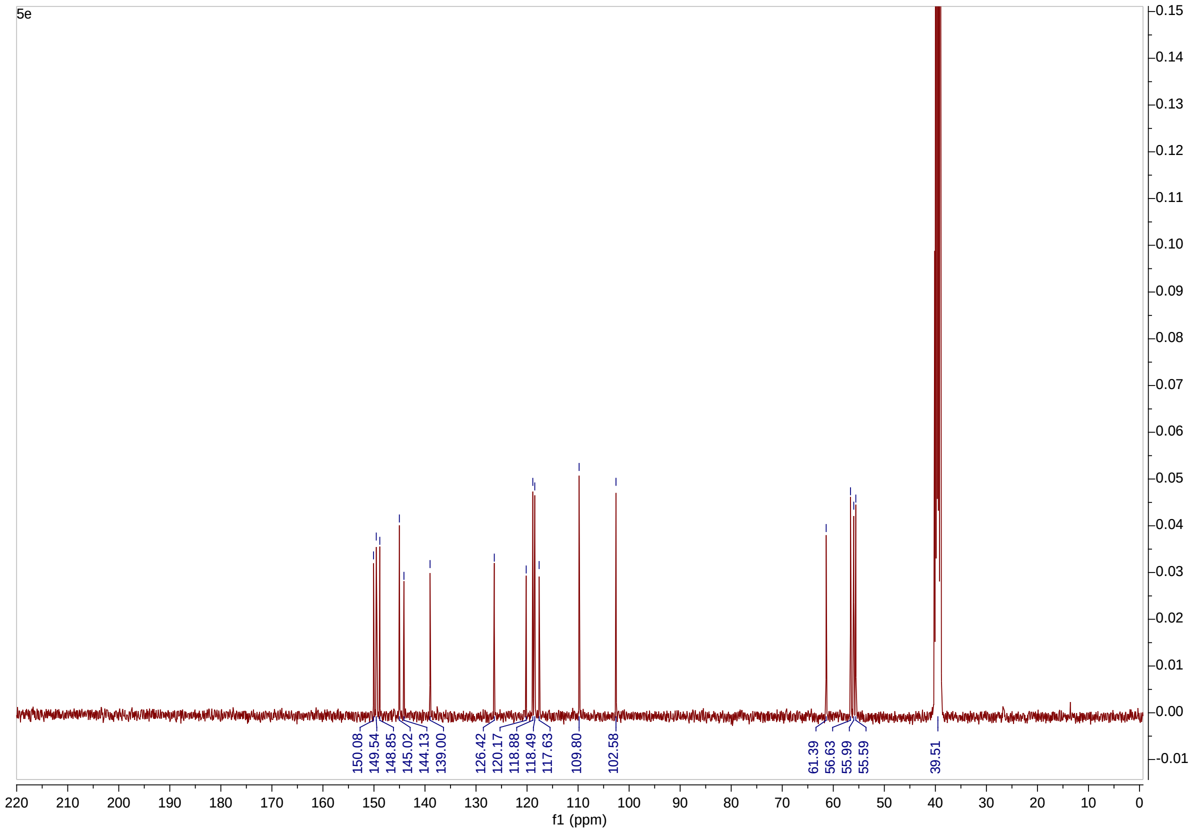

Compound 5f (DMSO-d_6)

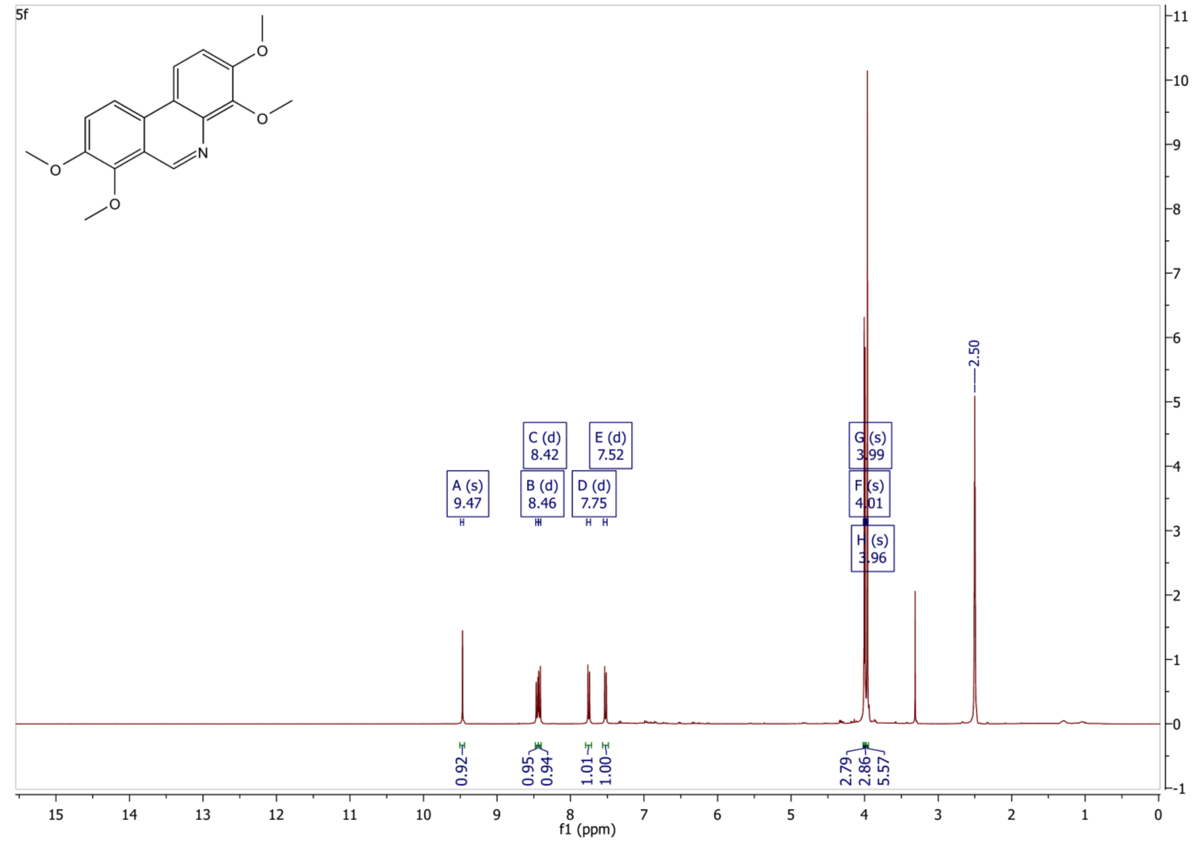

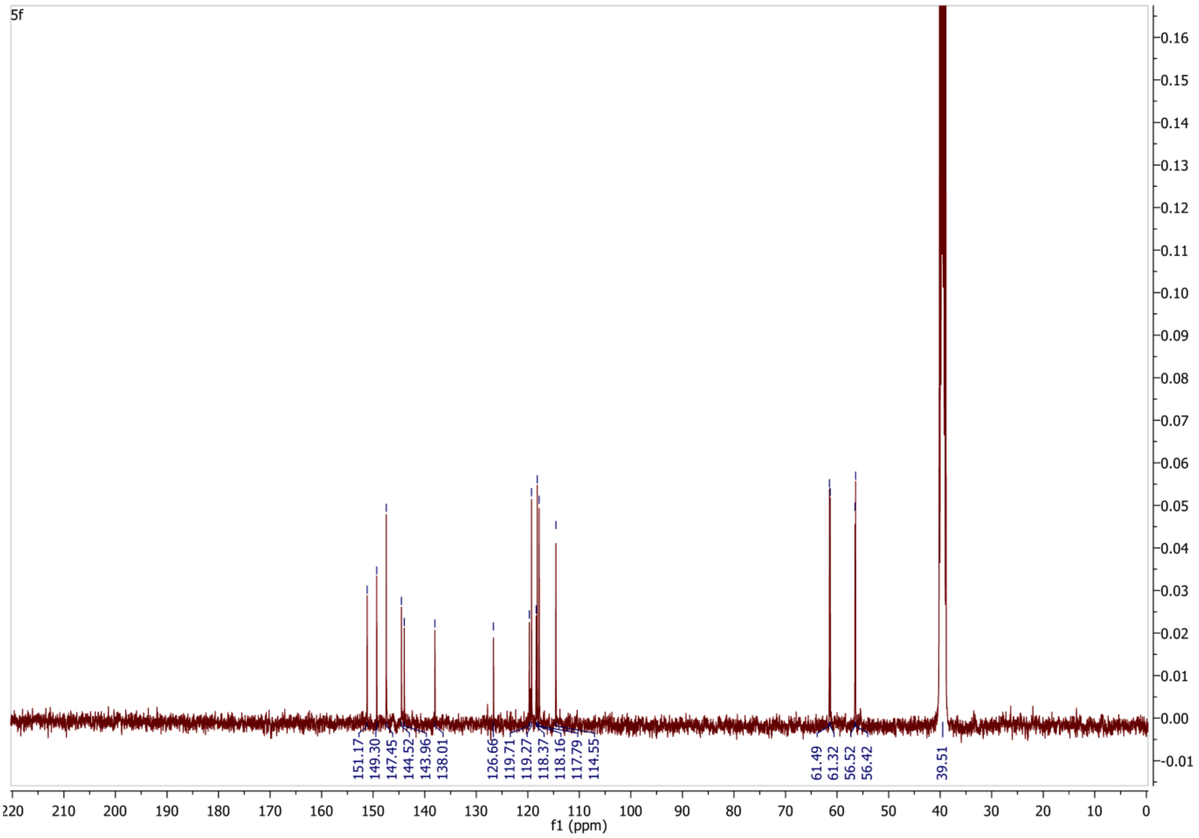

Compound 5g (DMSO-d_6)

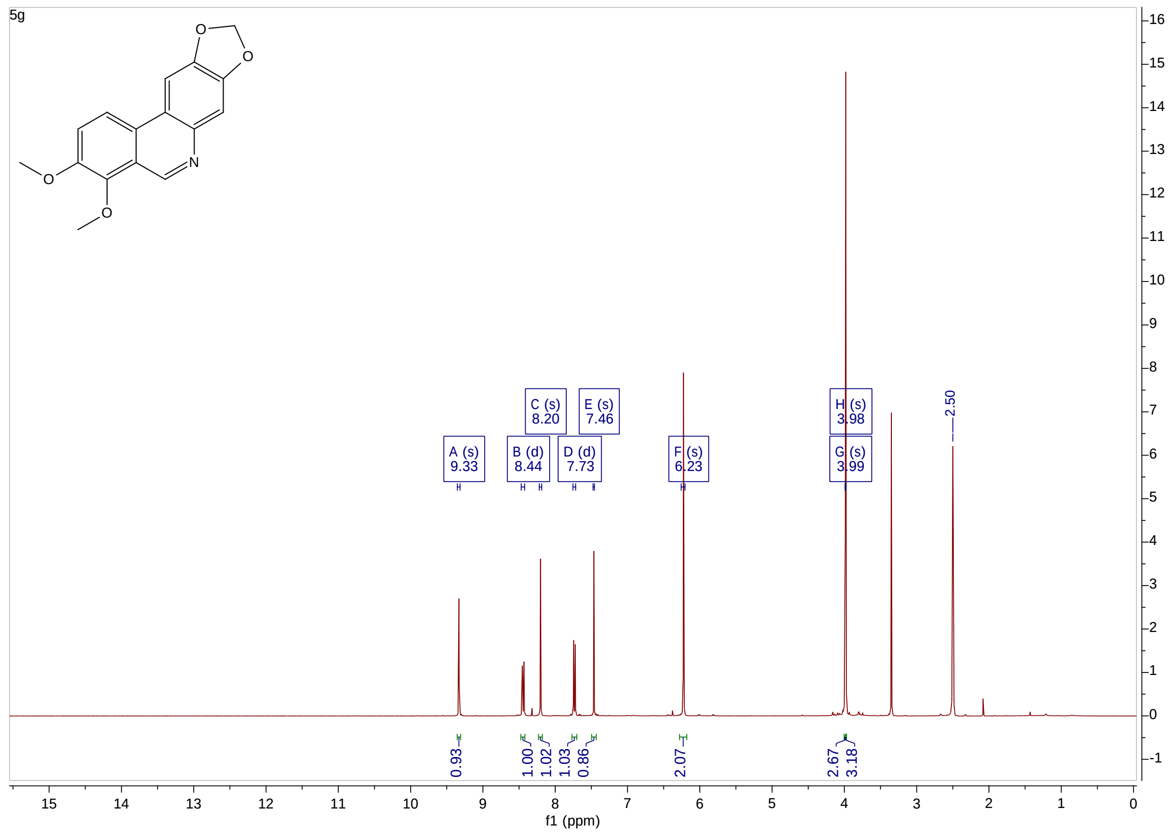

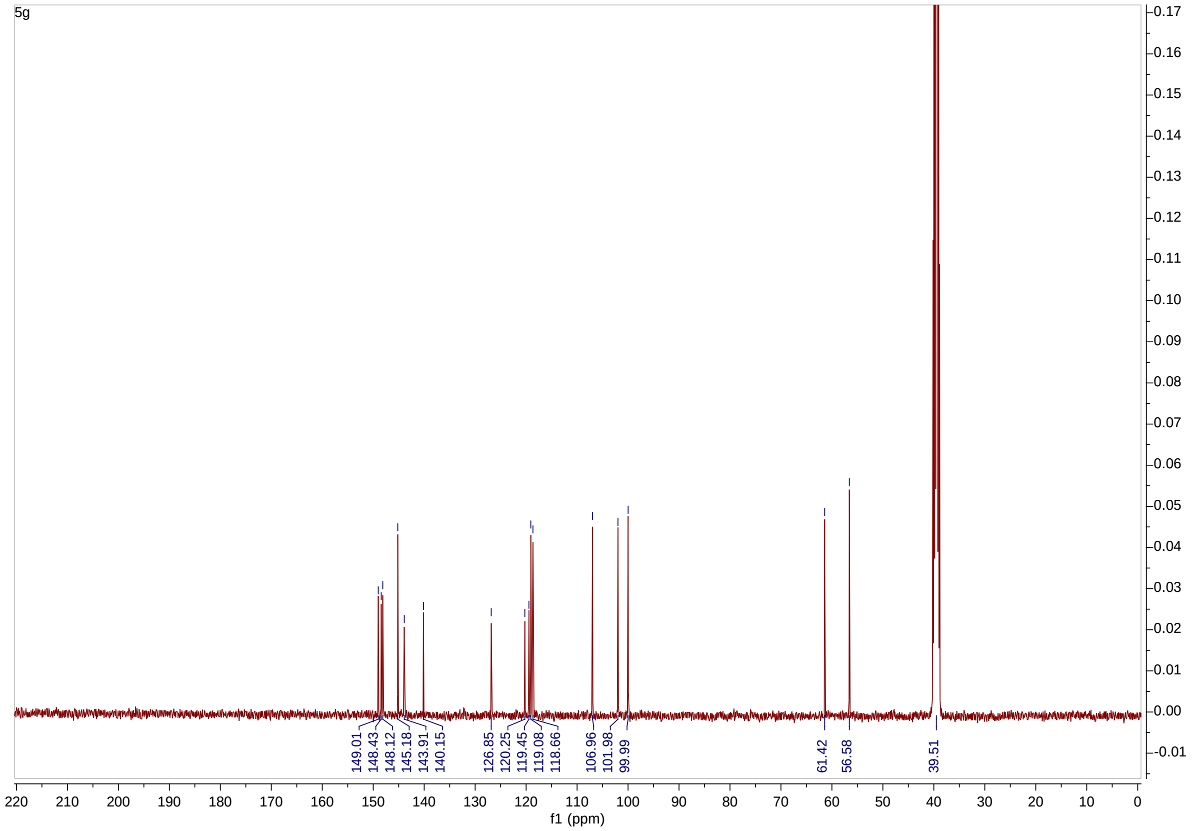

Compound 5h (DMSO-d_6)

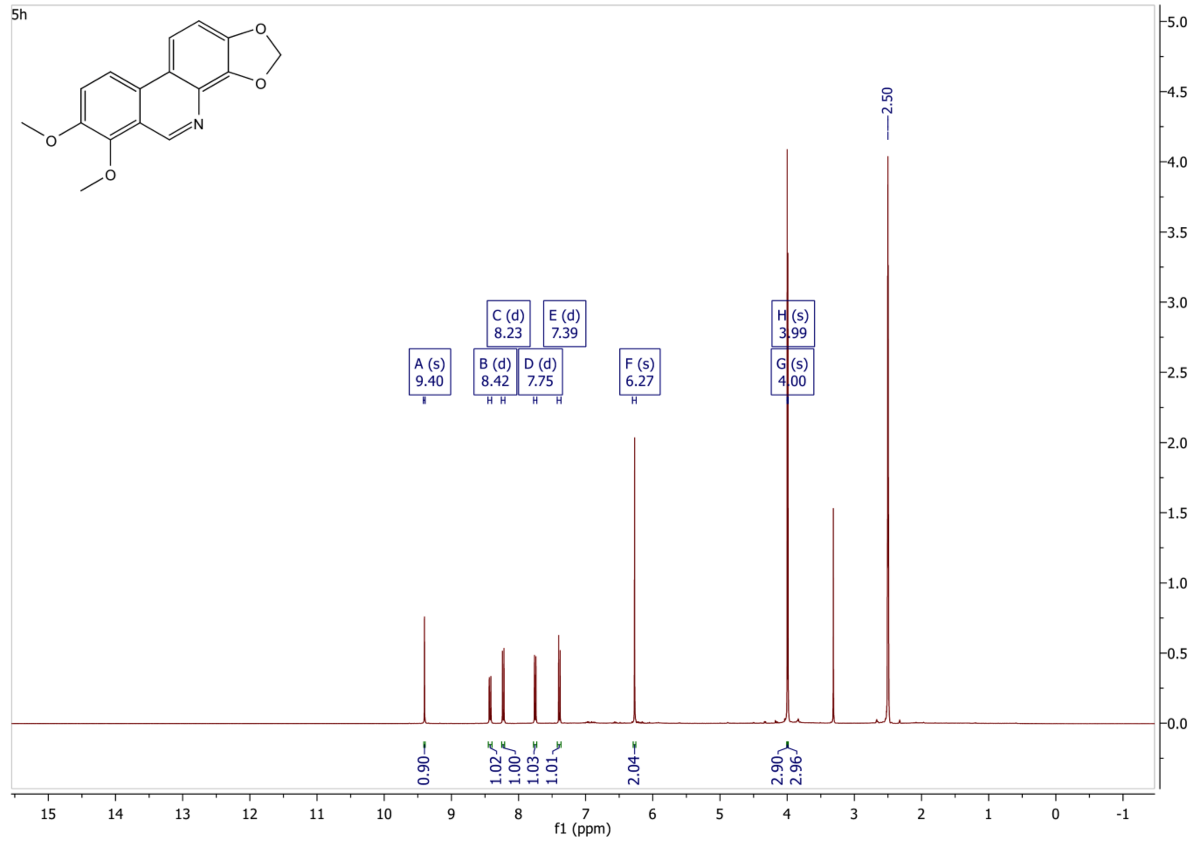

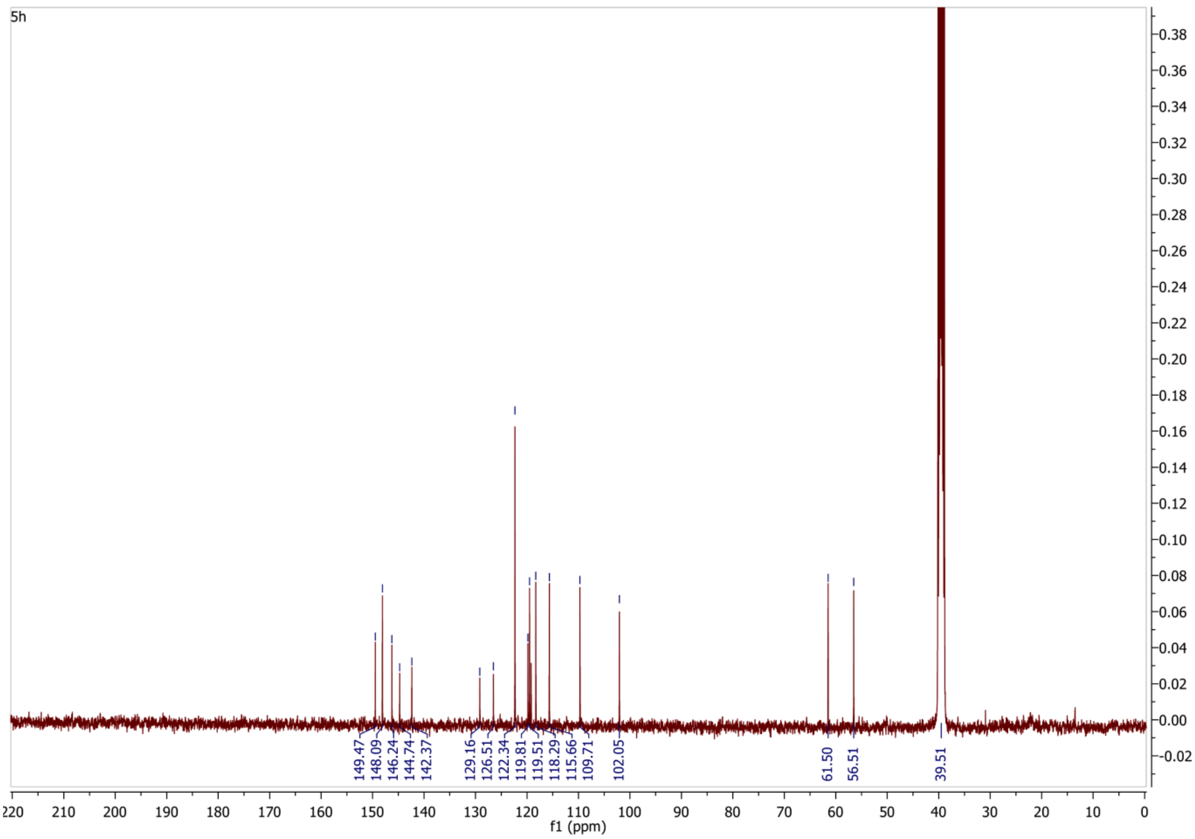

Compound 5i (DMSO-d_6)

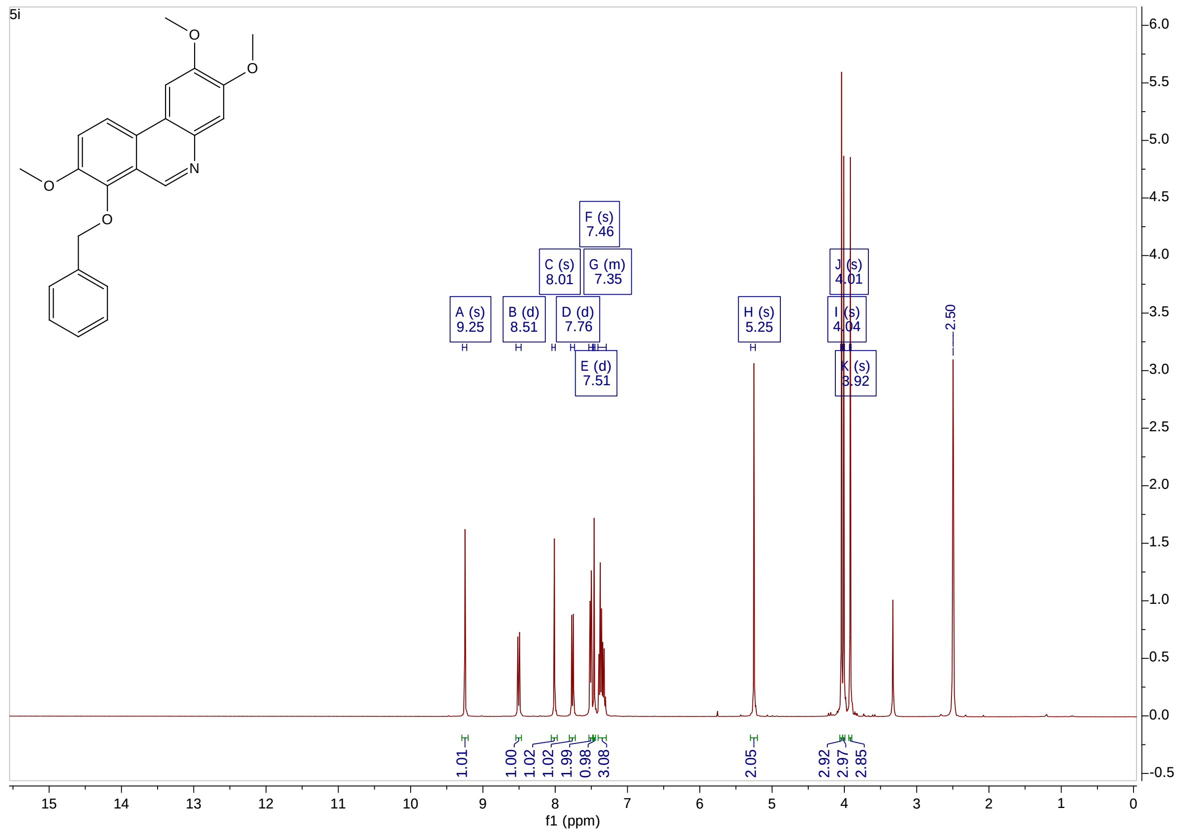

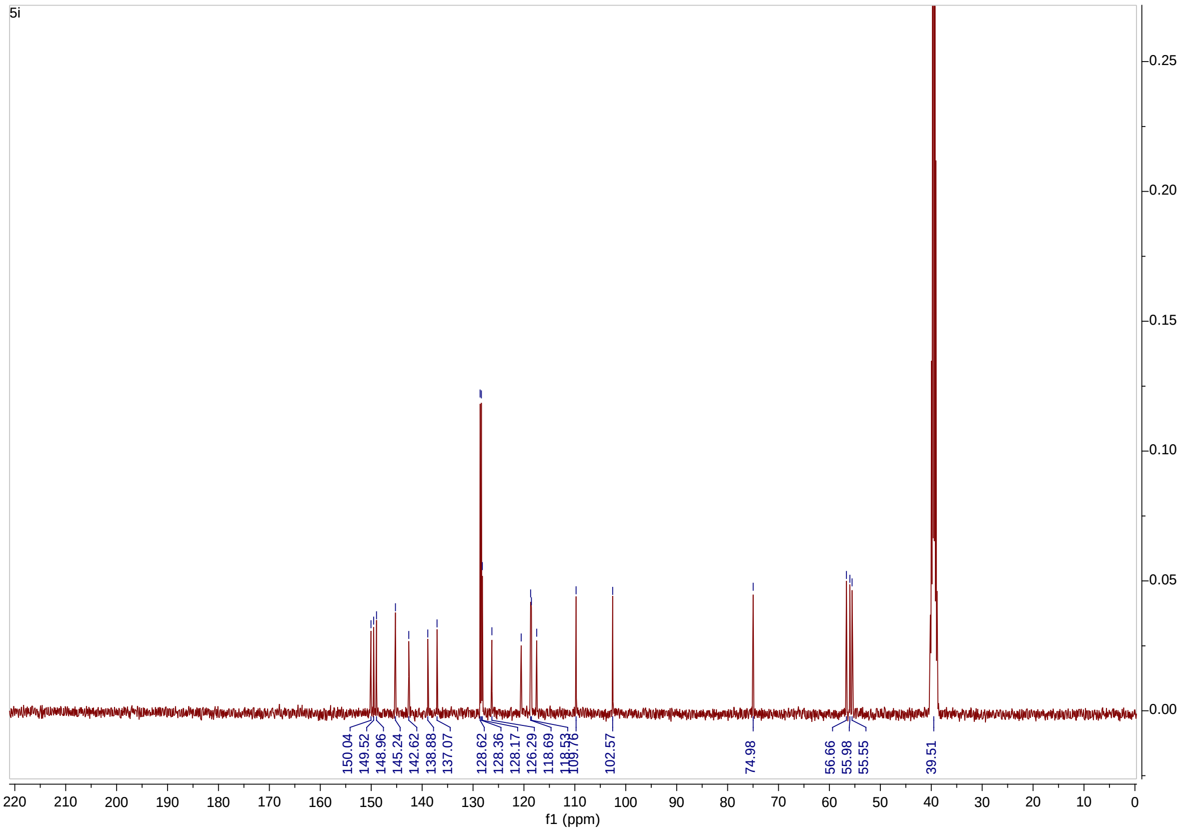

Compound 5j (DMSO-d_6)

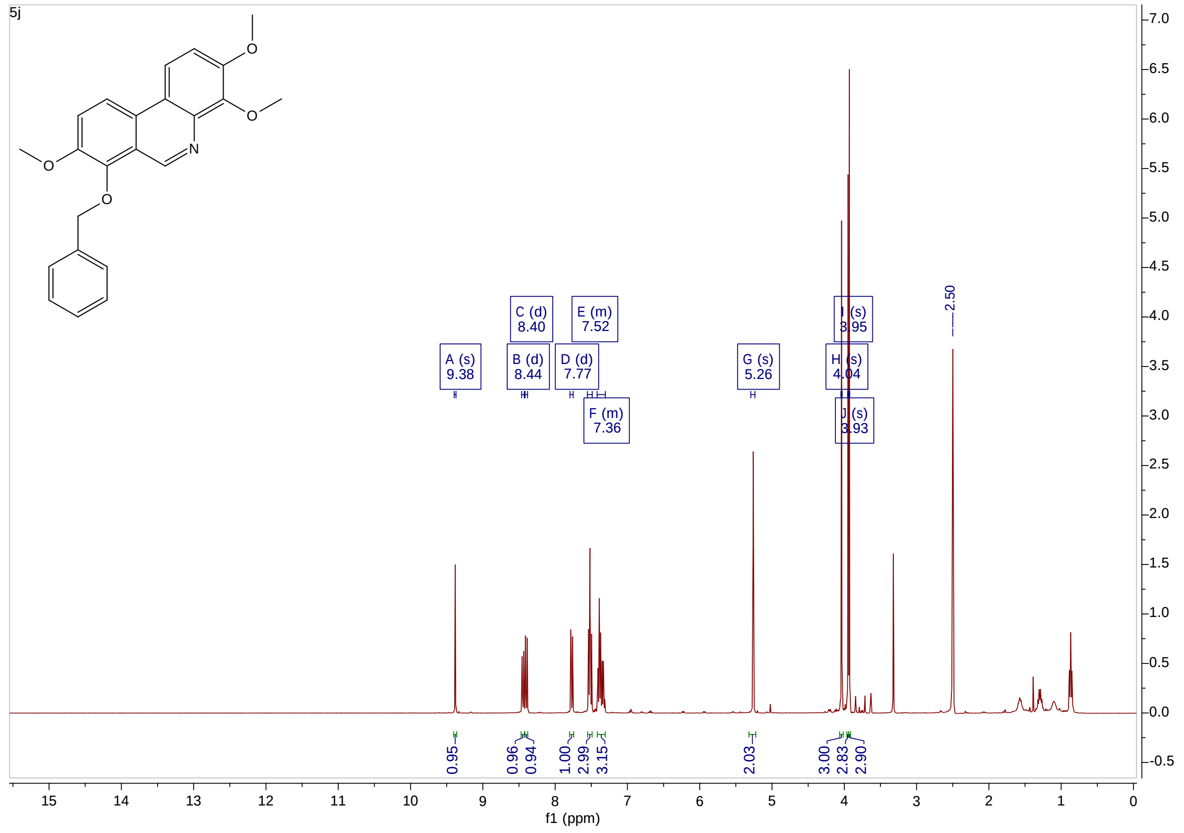

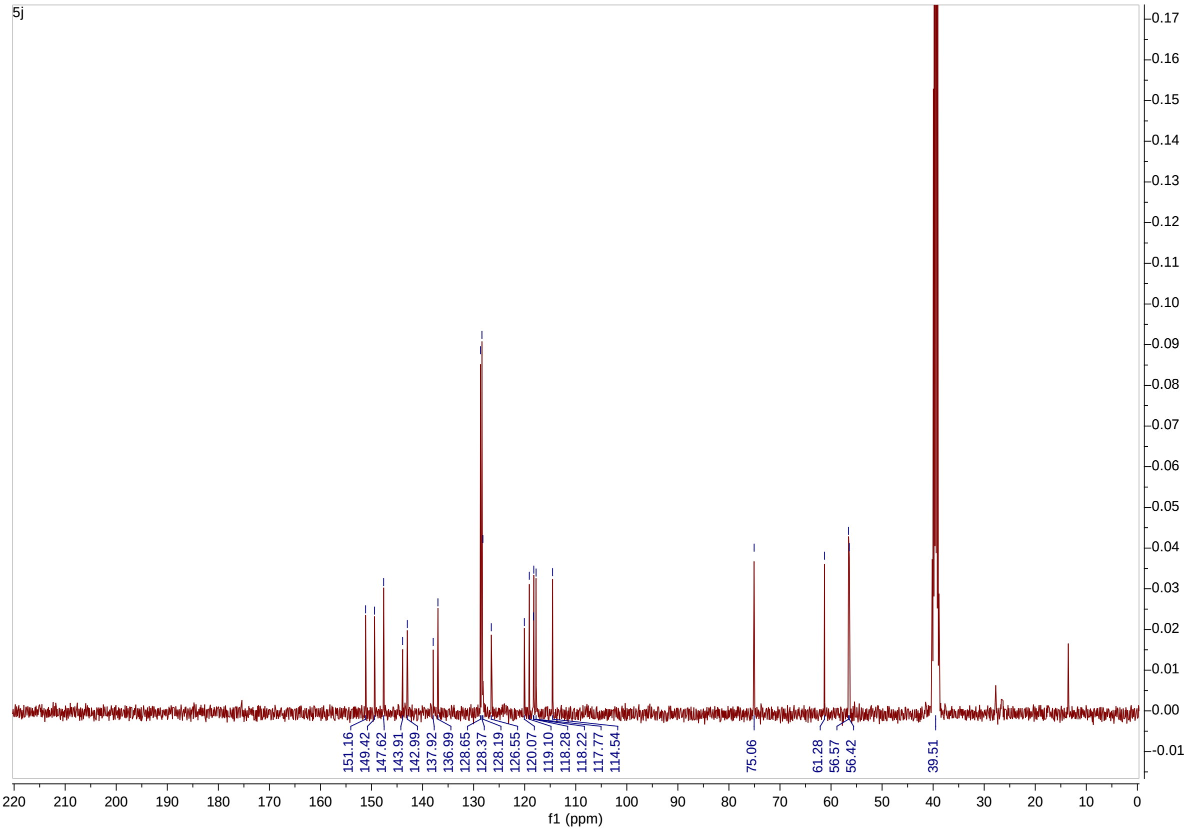

Compound 5k (DMSO-d_6)

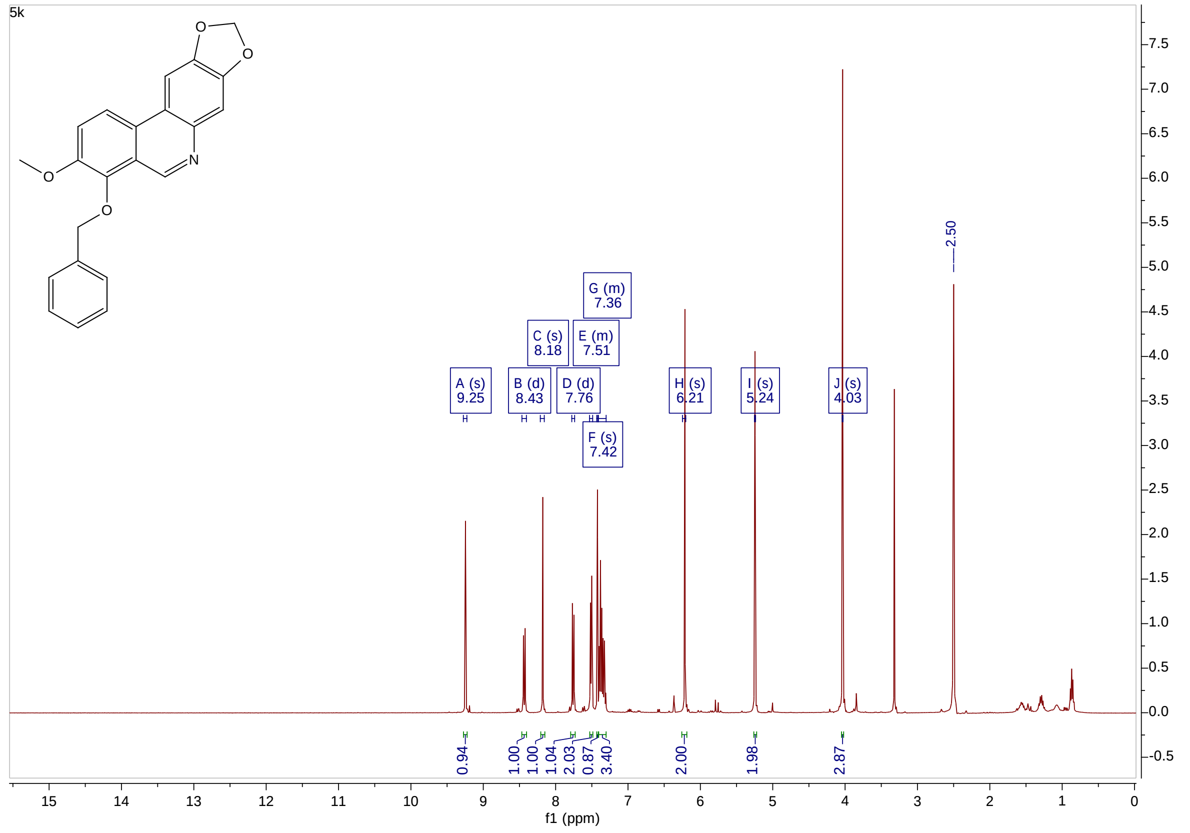

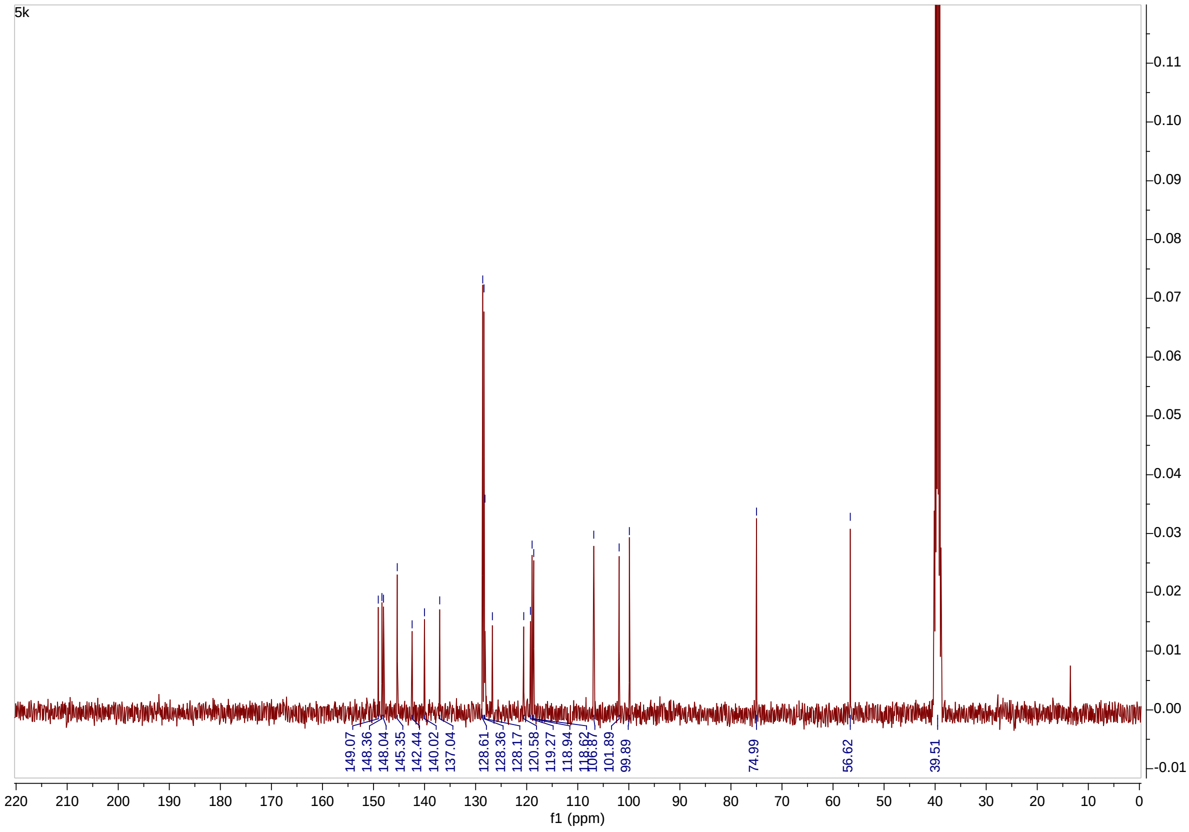

Compound 5l (DMSO-d_6)

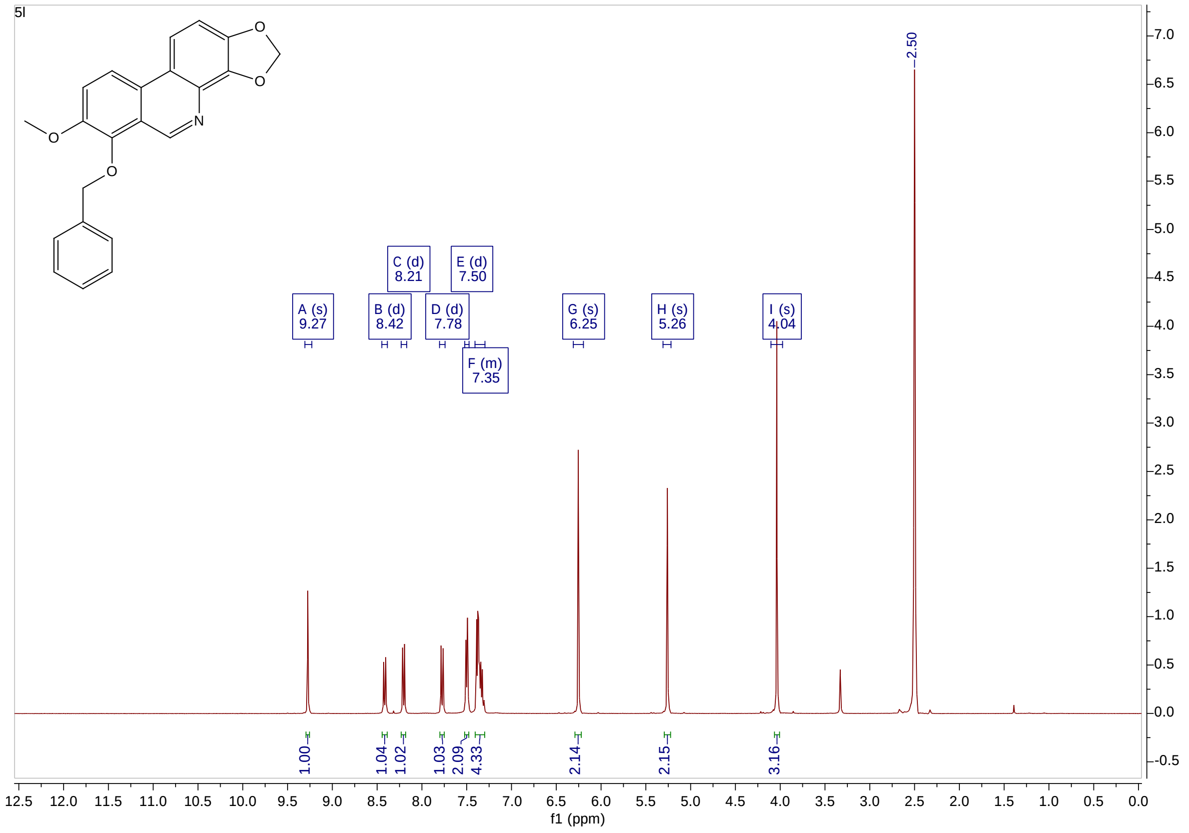

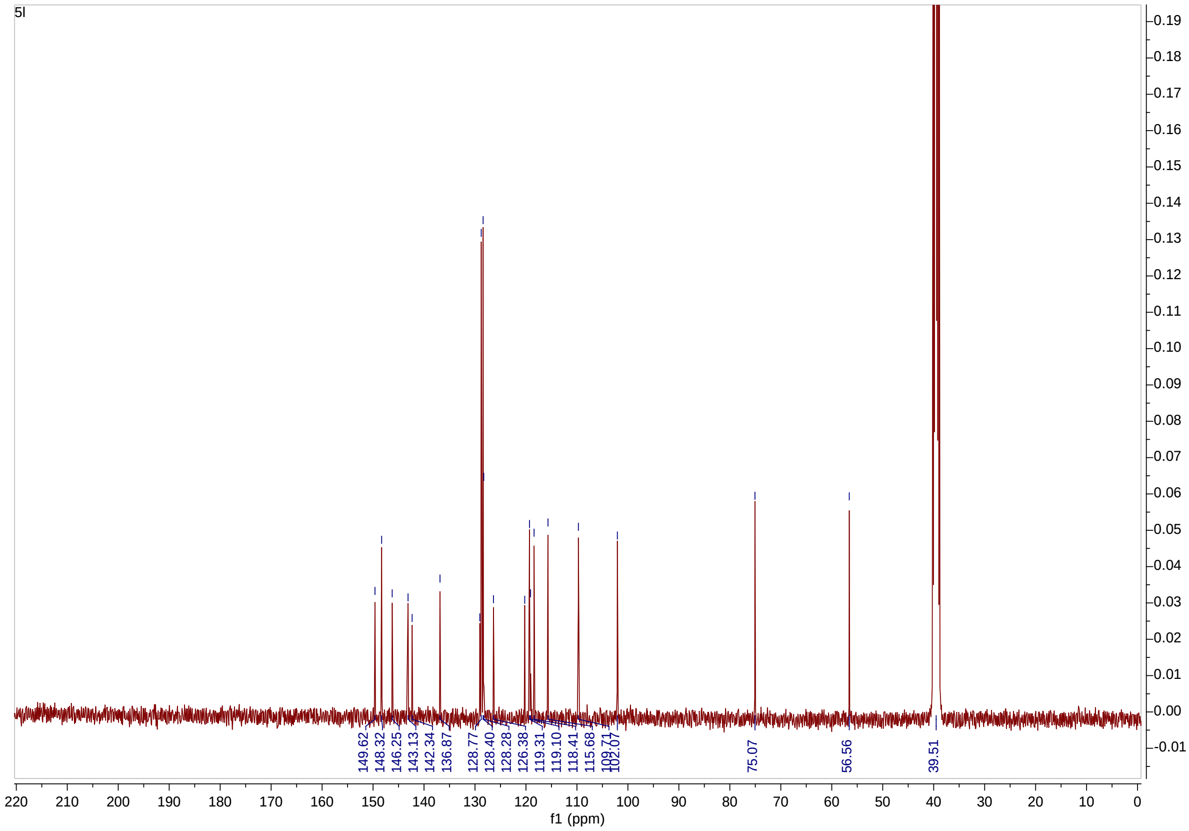

Compound 7c (DMSO-d_6)

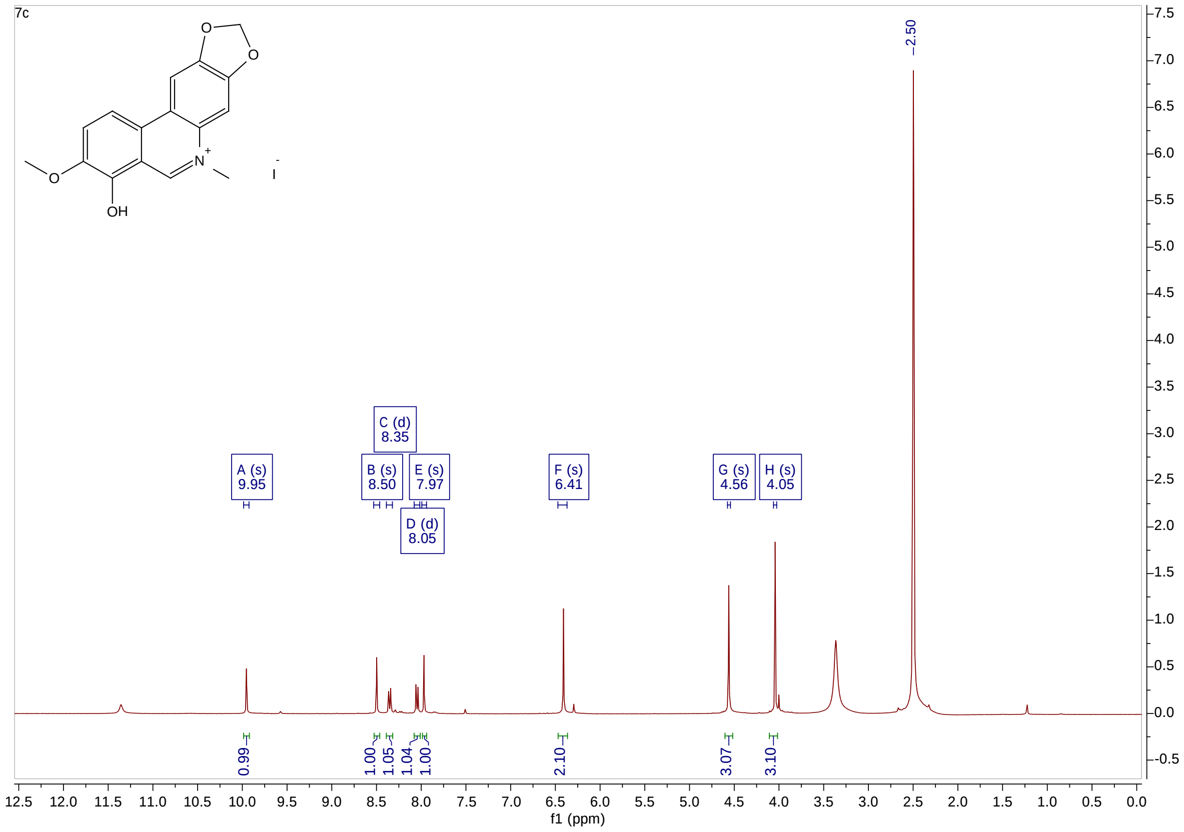

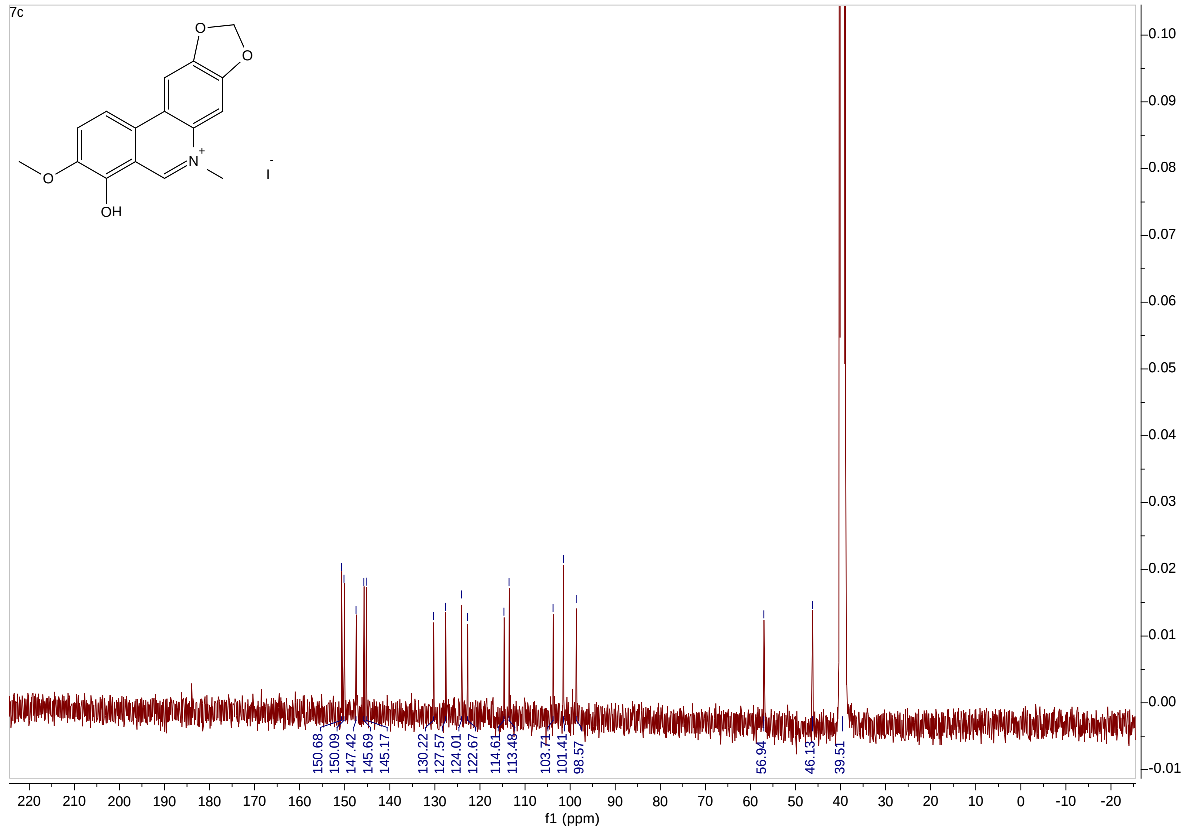

Compound 7d (DMSO-d_6)

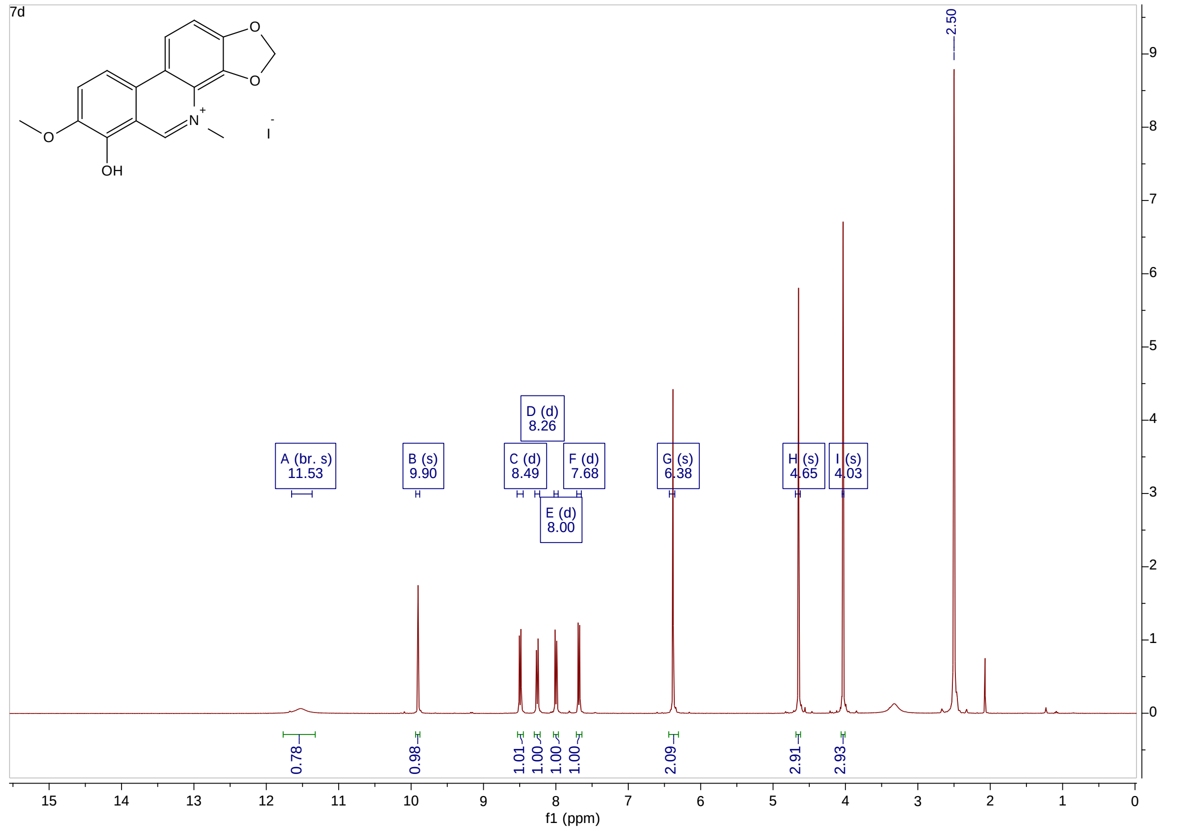

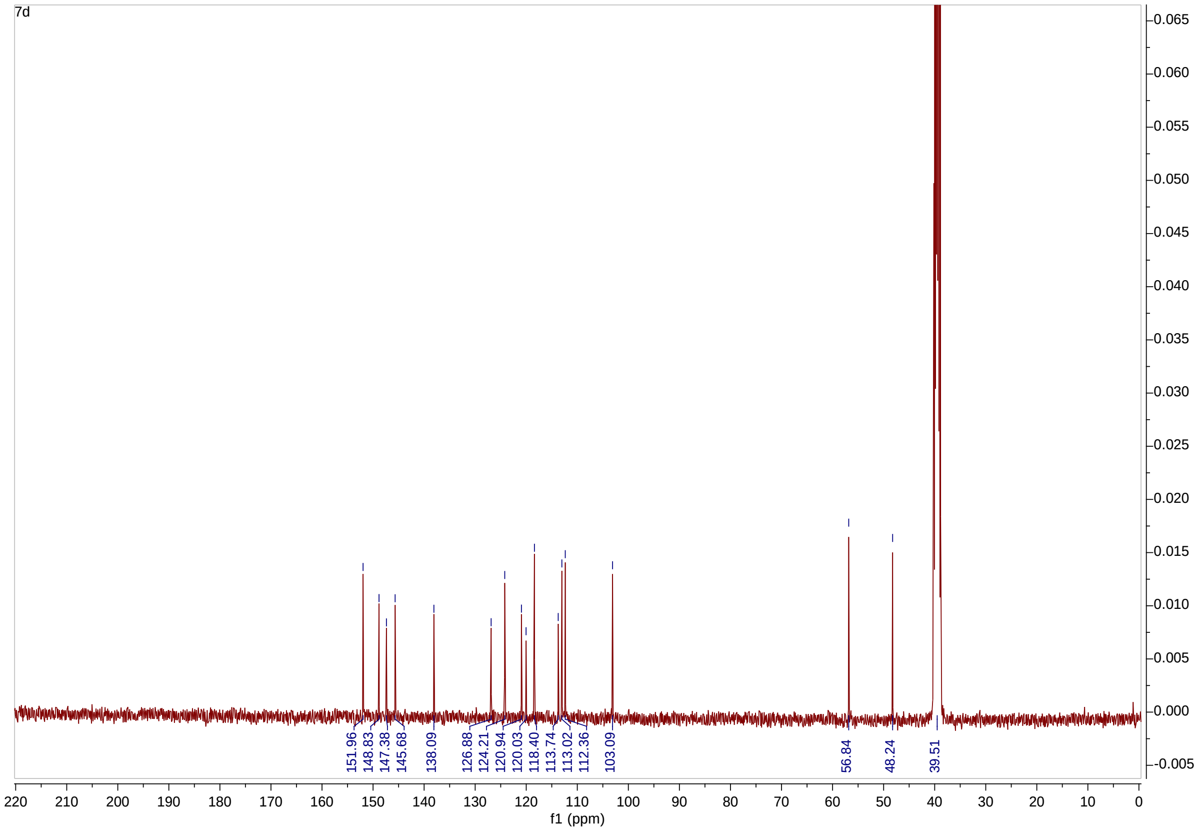

Compound 7e (DMSO-d_6)

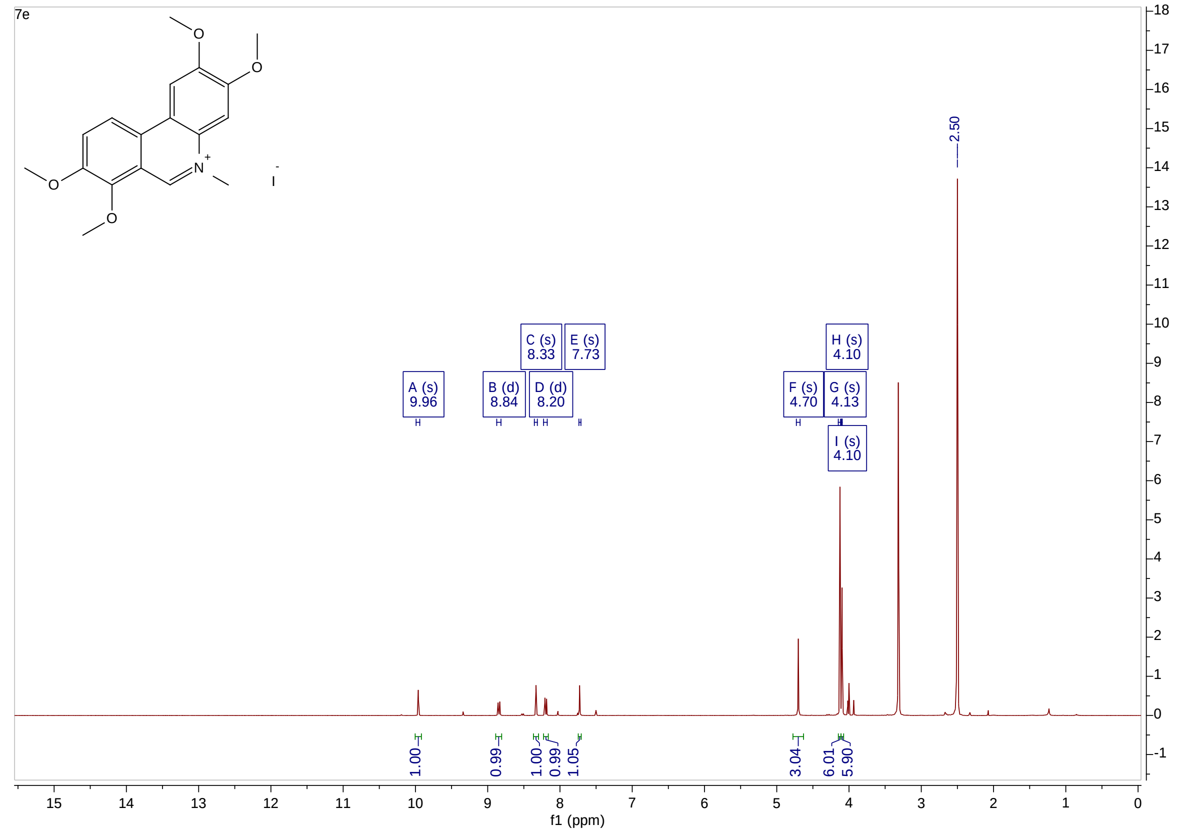

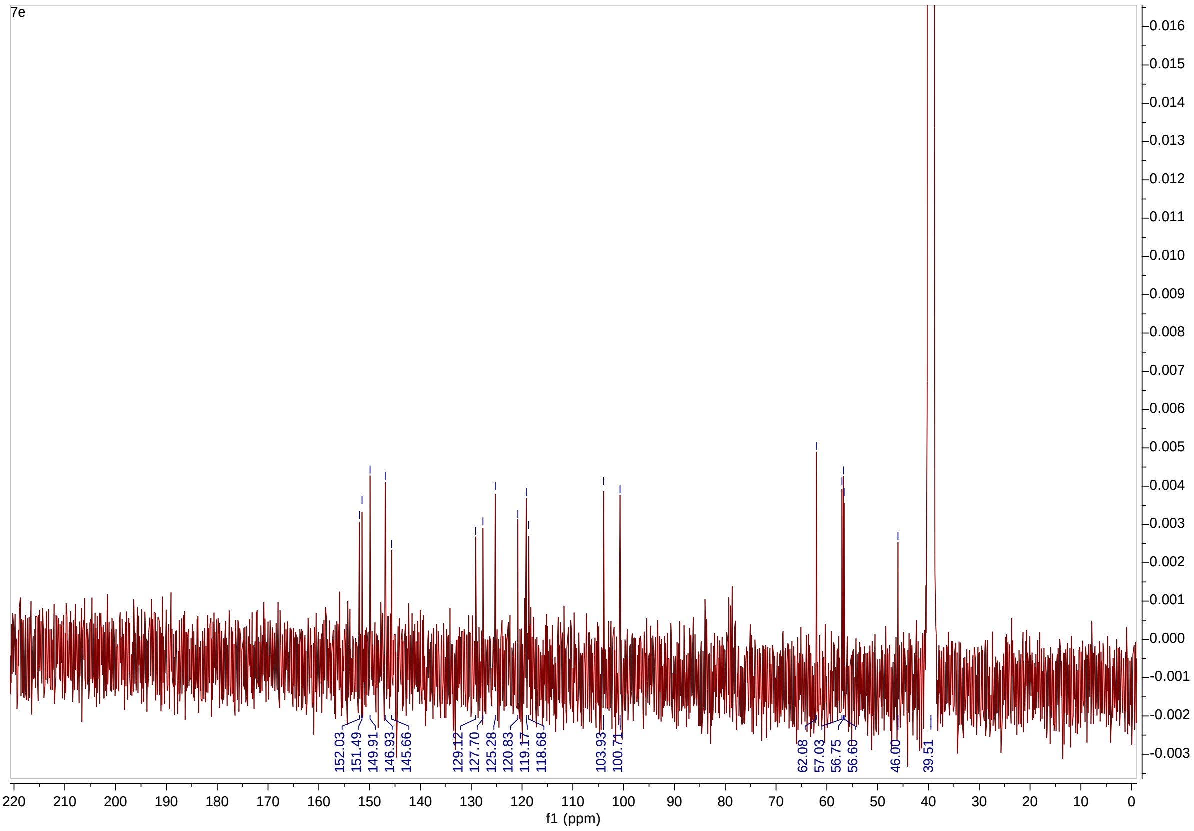

Compound 7f (DMSO-d_6)

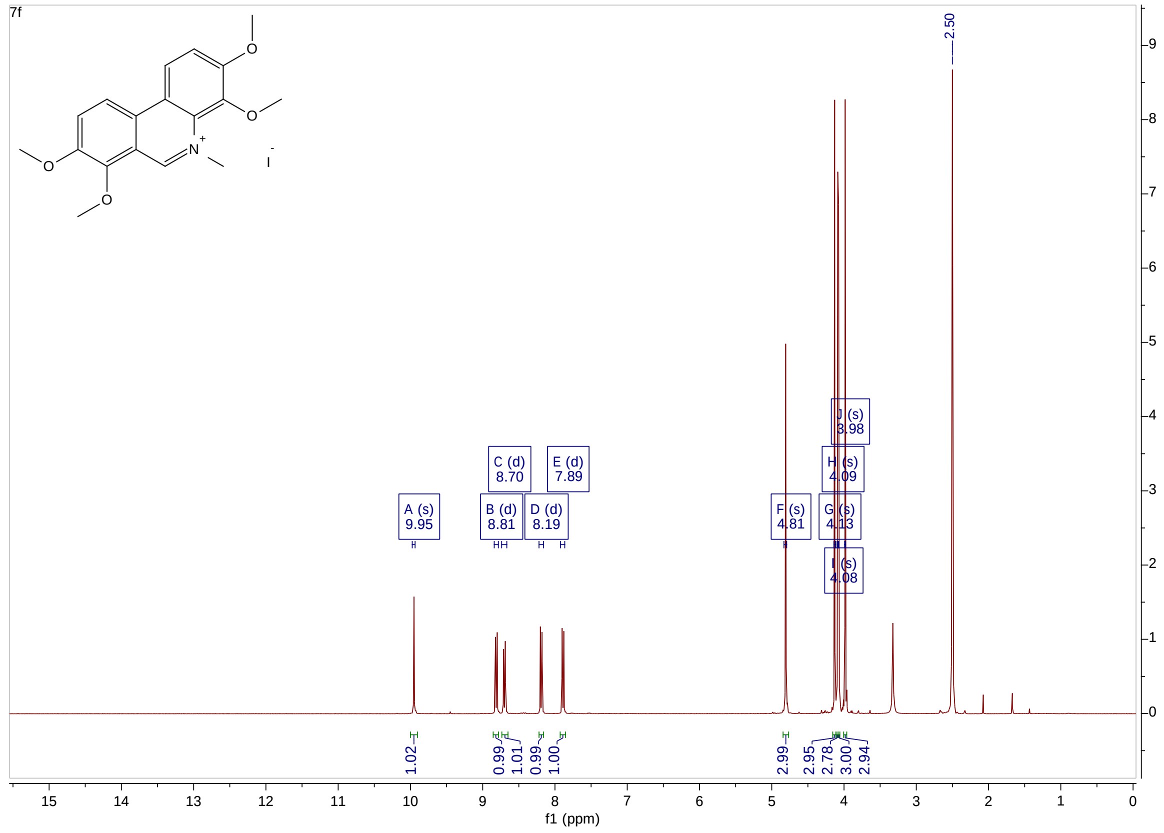

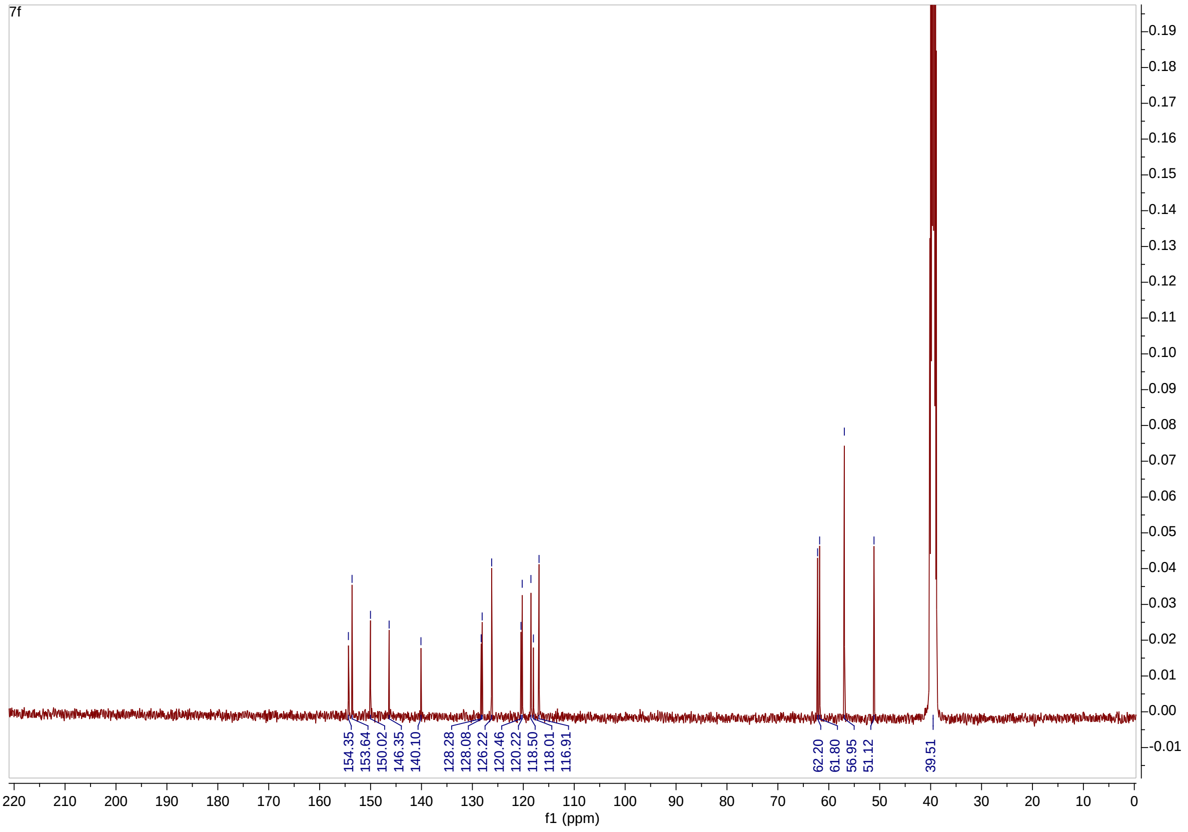

Compound 7g (DMSO-d_6)

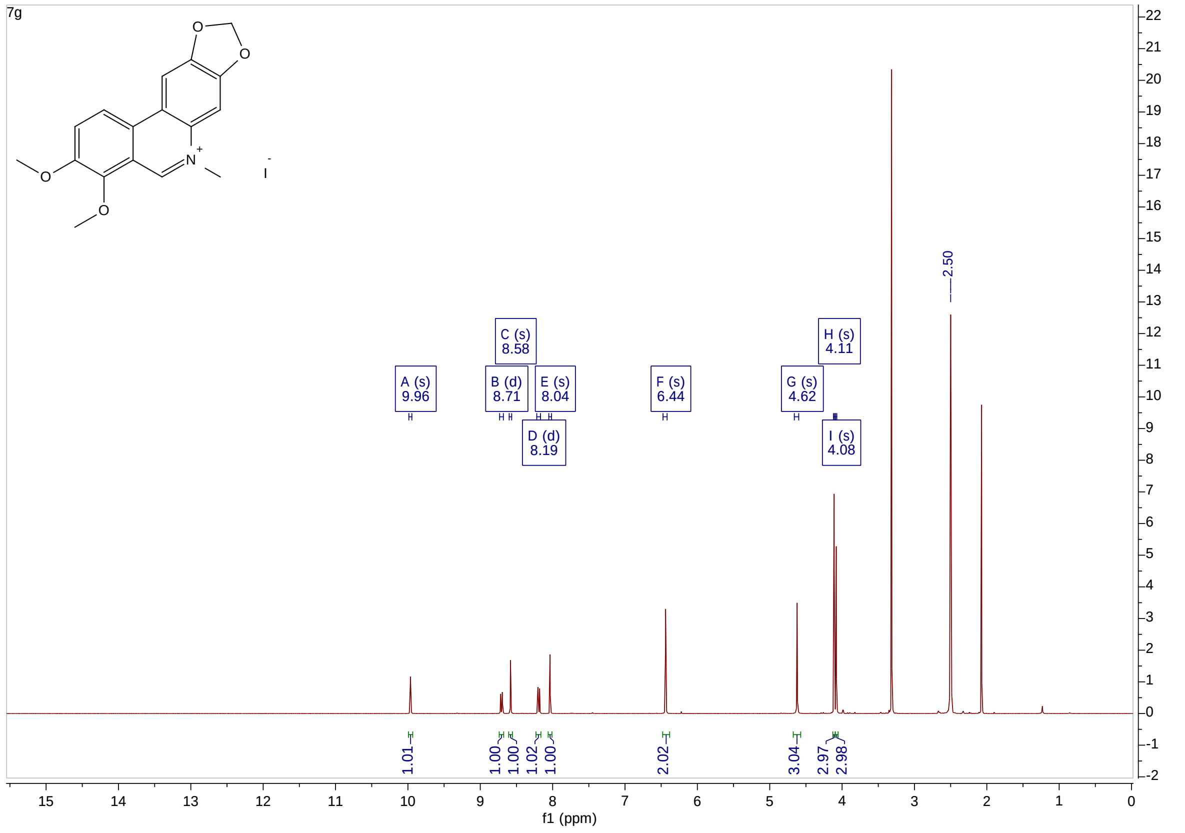

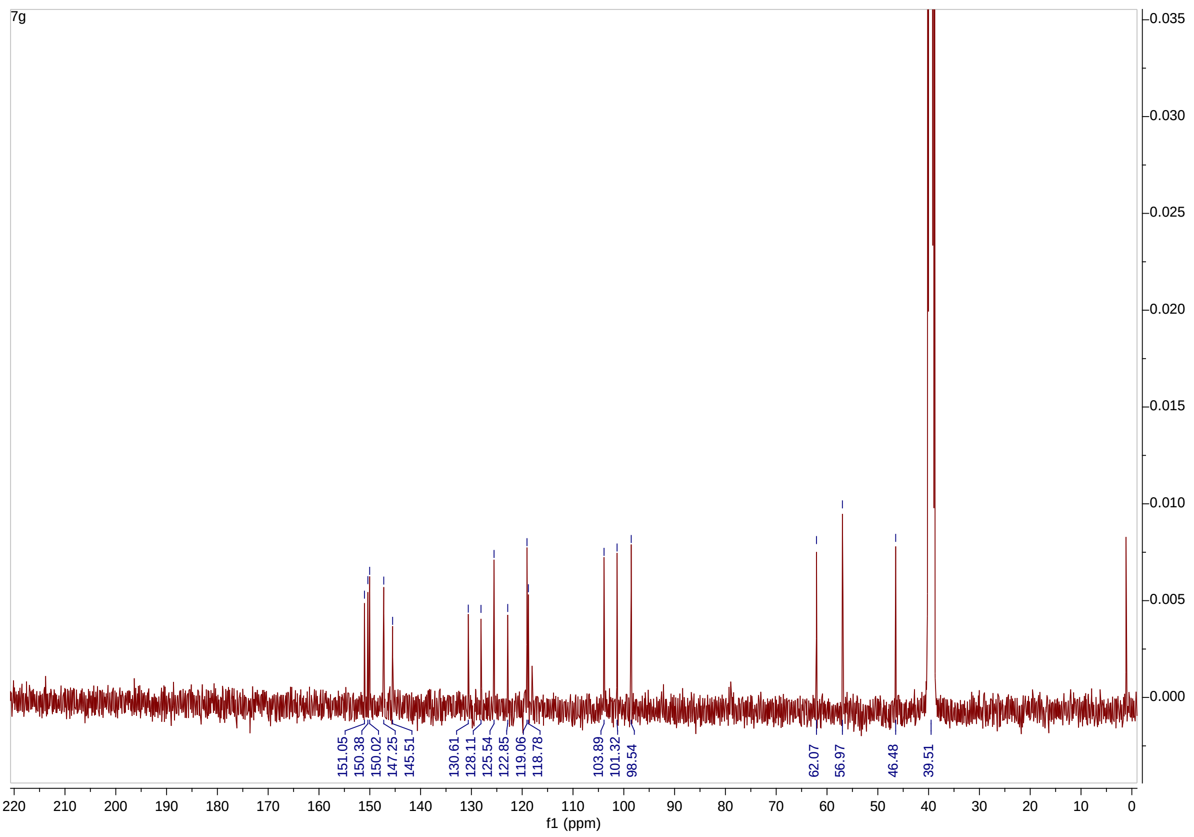

Compound 7h (DMSO-d_6)

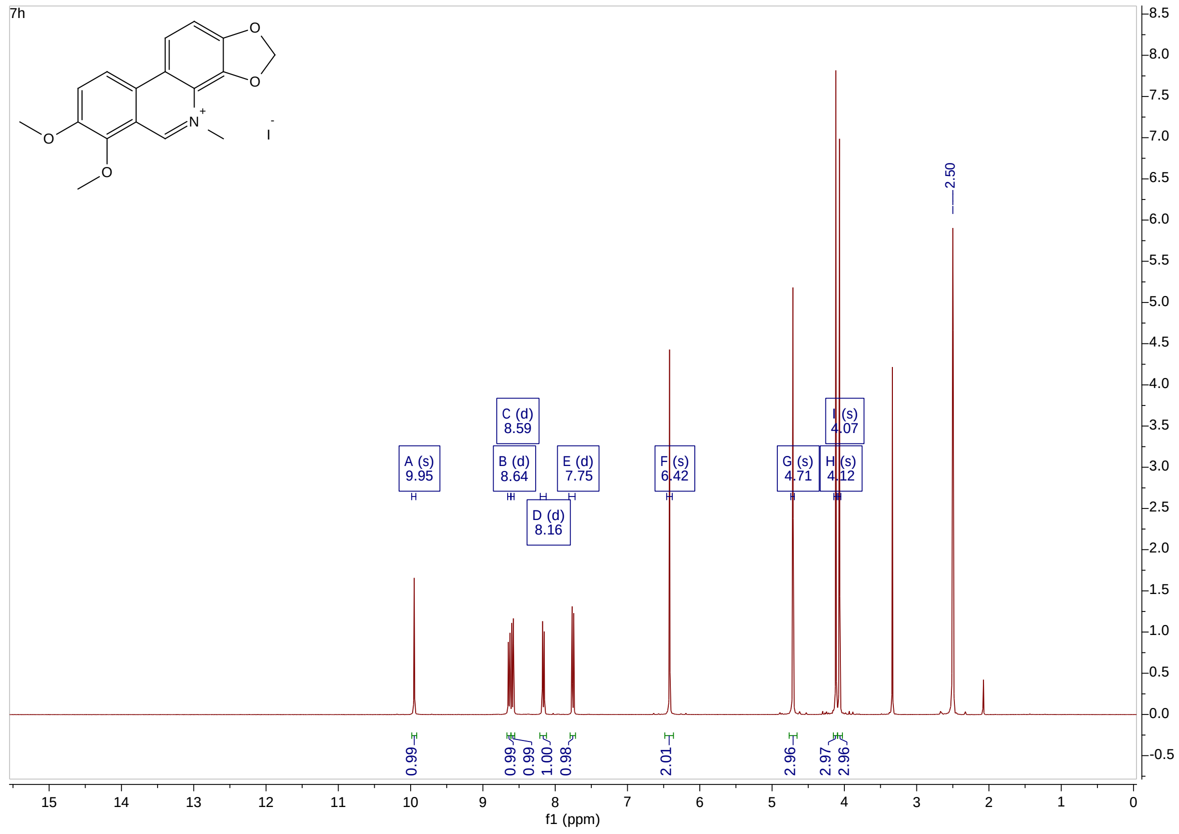

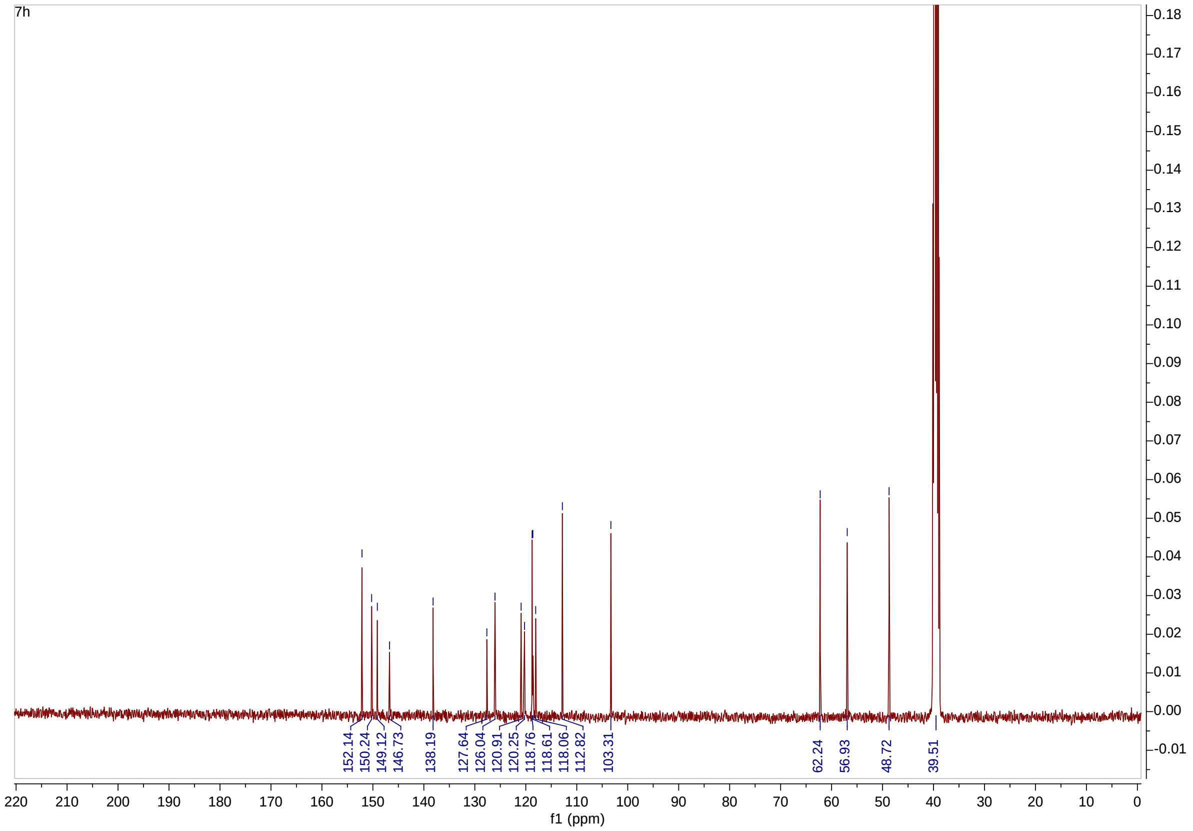

Compound 7i (DMSO-d_6)

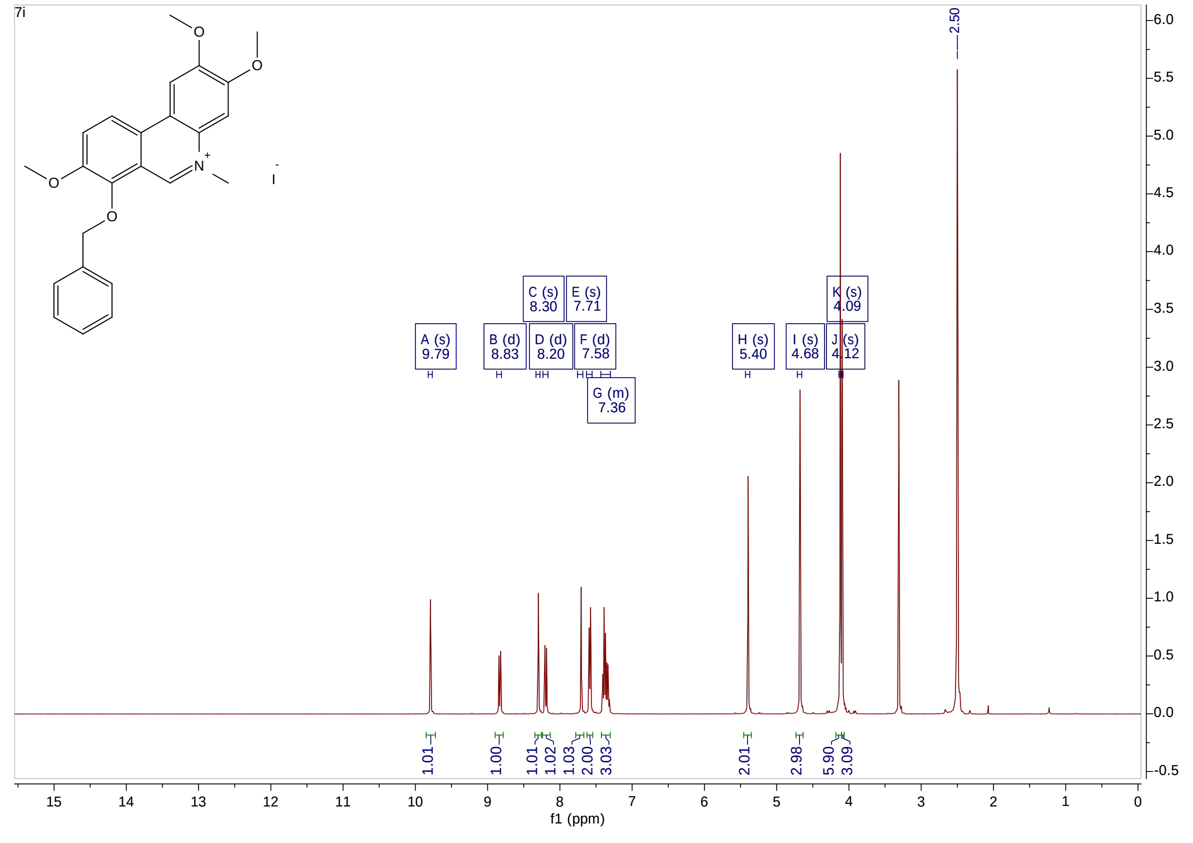

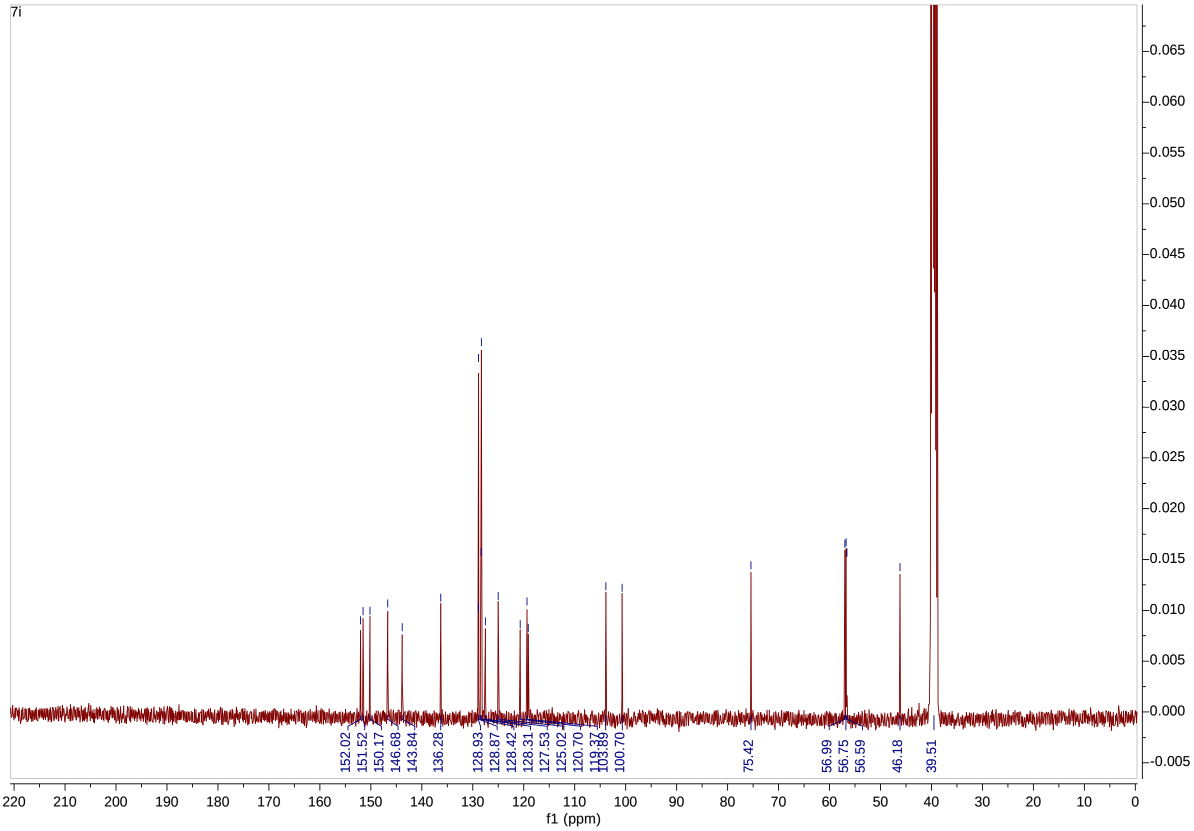

Compound 7j (DMSO-d_6)

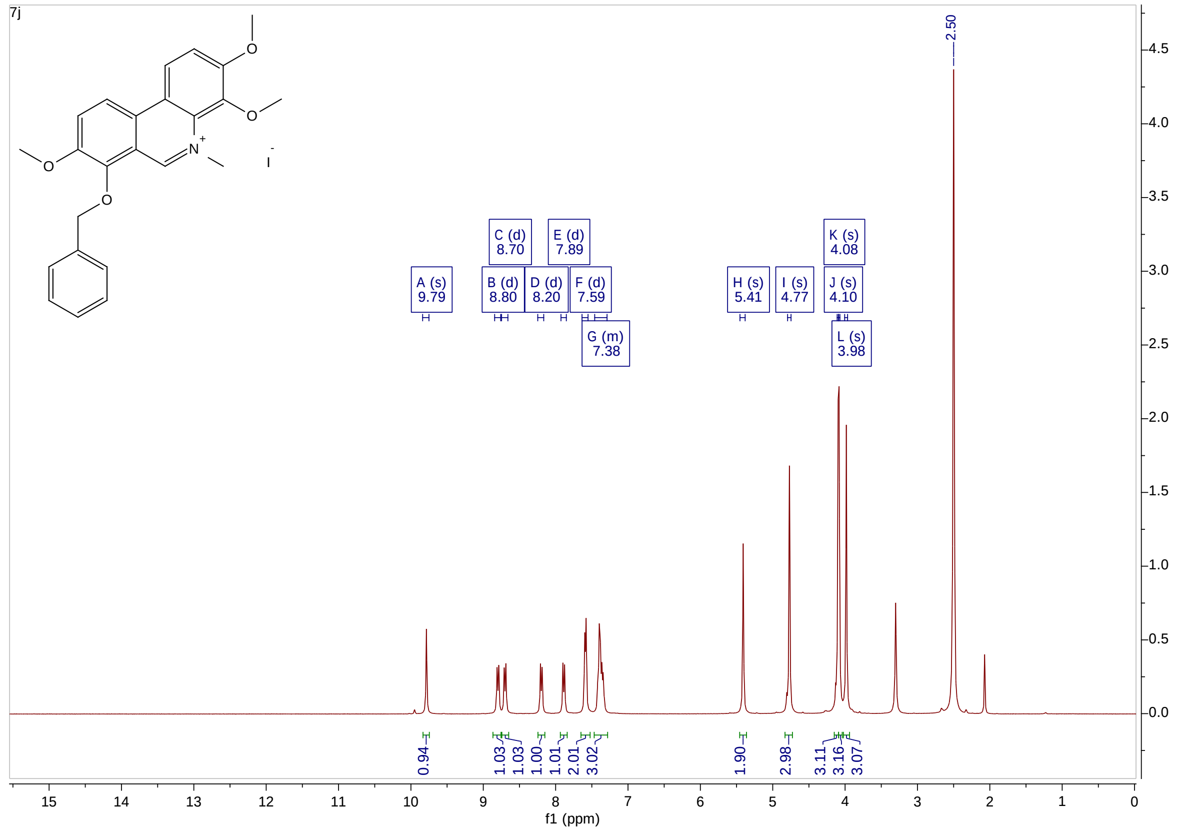

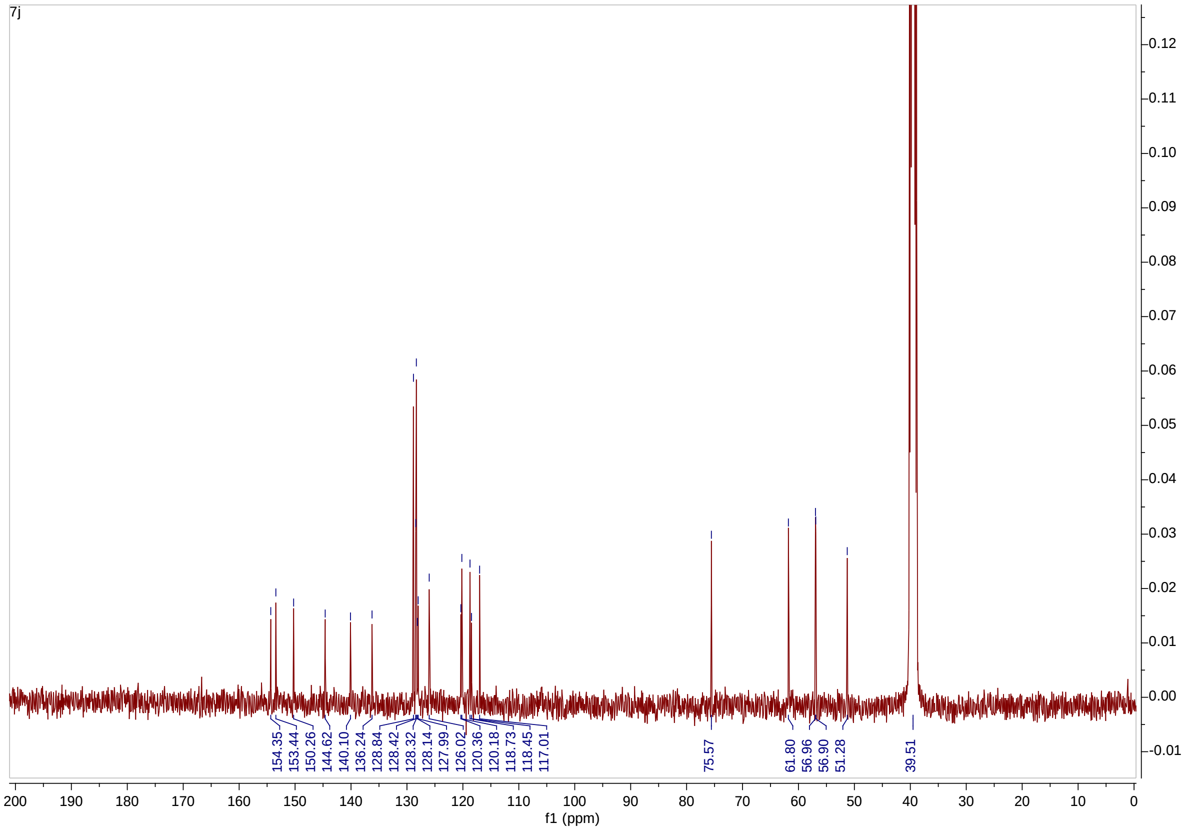

Compound 7k (DMSO-d_6)

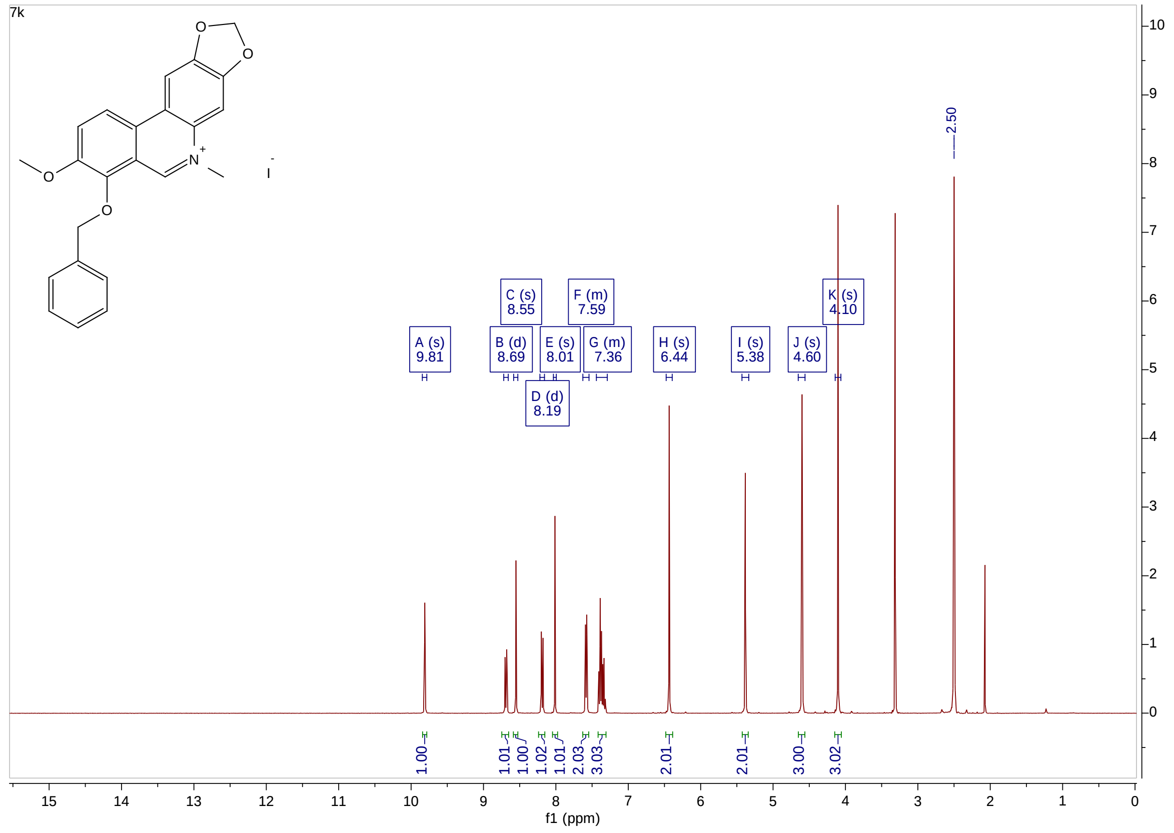

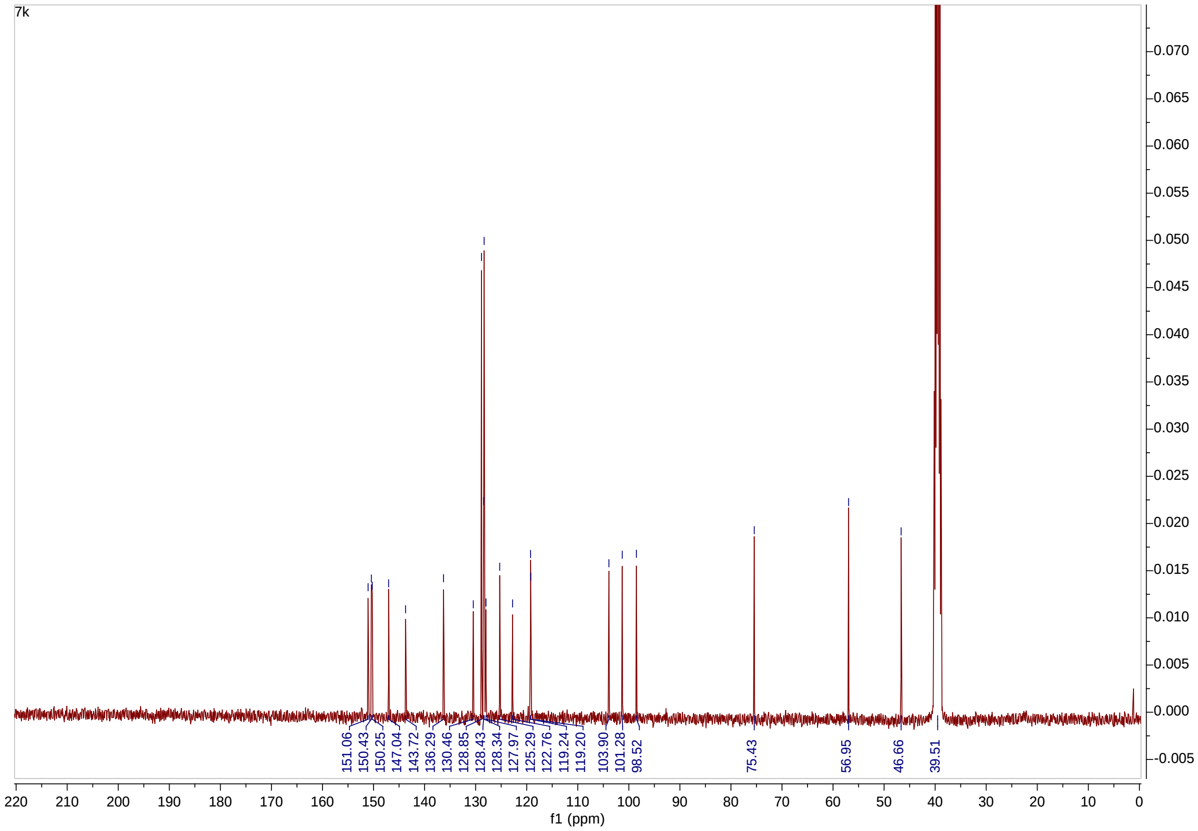

Compound 7l (DMSO-d_6)

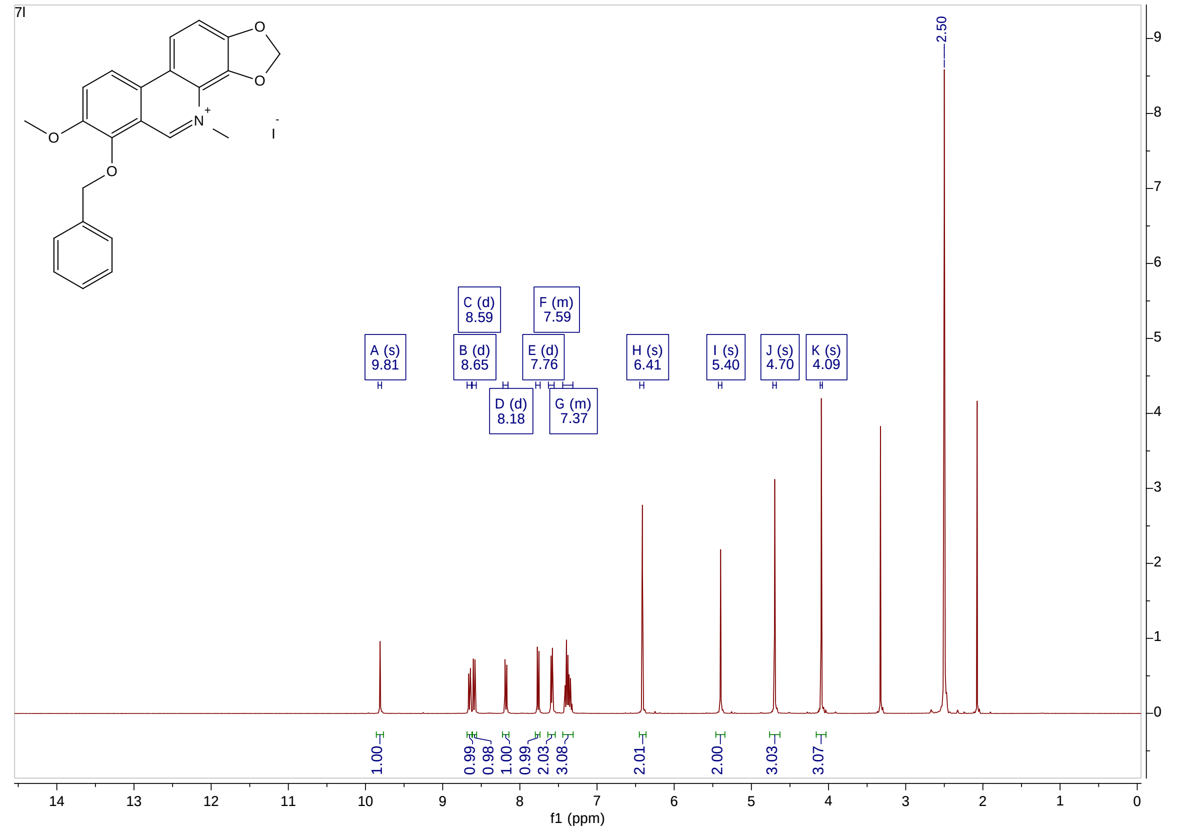

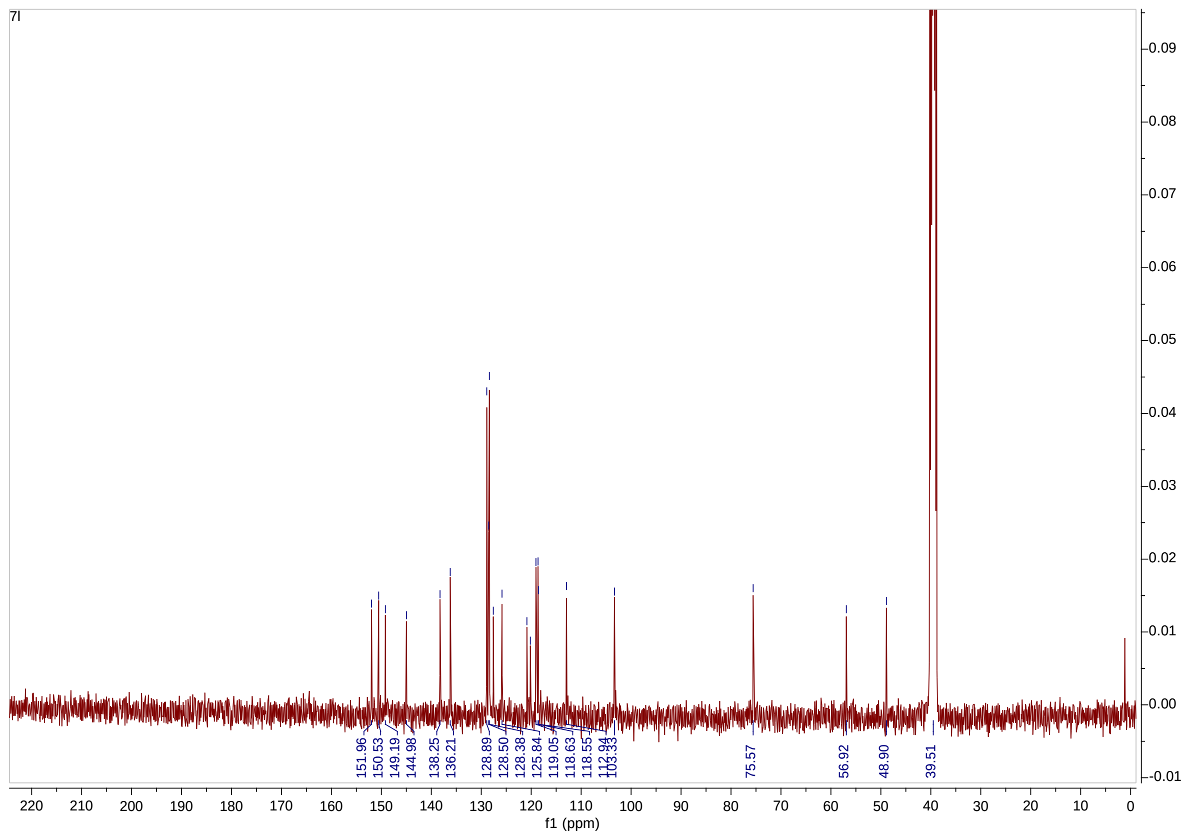

Compound 9t (DMSO-d_6)

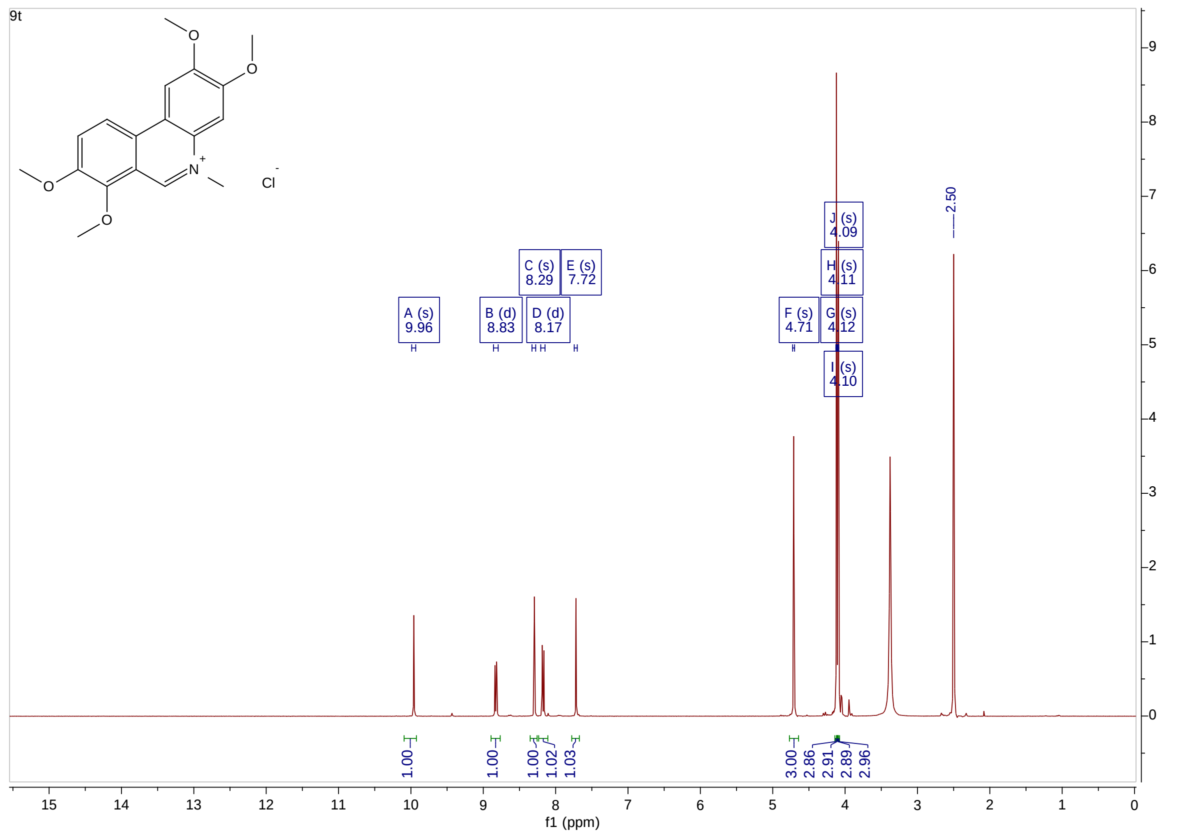

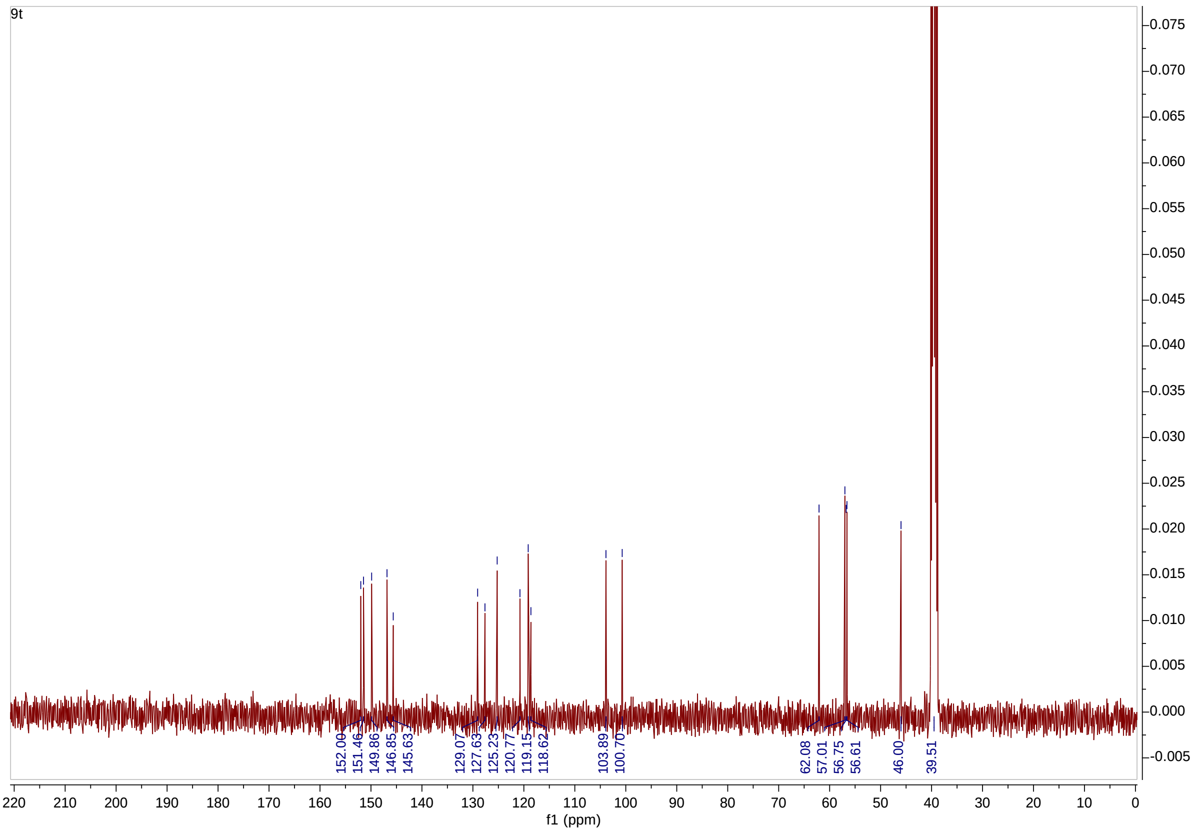

Compound 9u (DMSO-d_6)

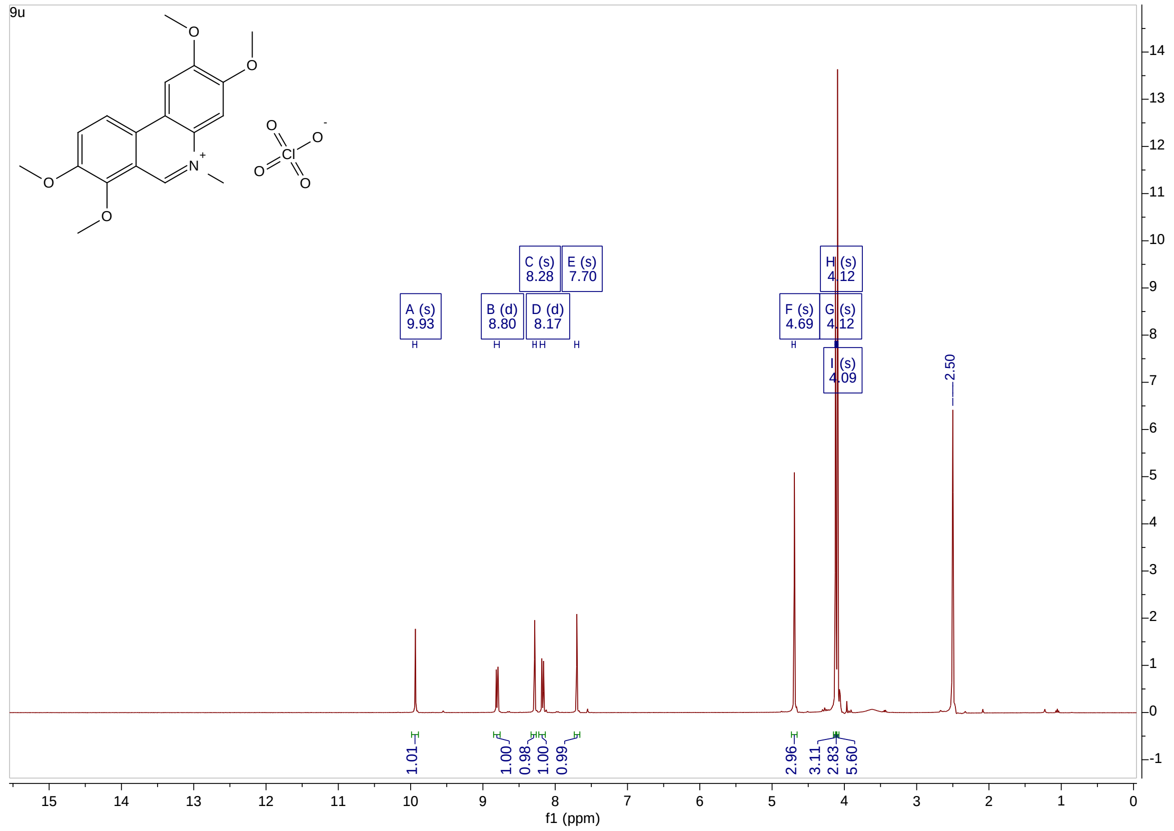

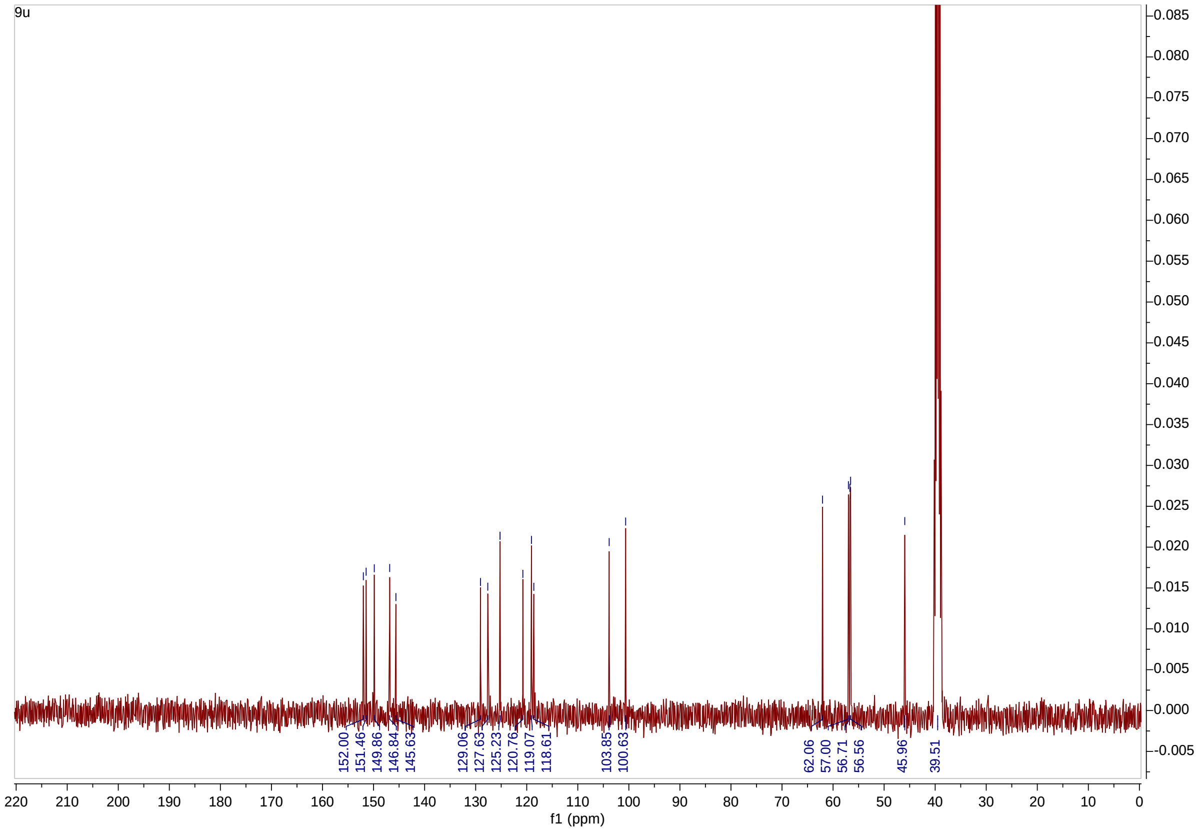

Compound 9w (DMSO-d_6)

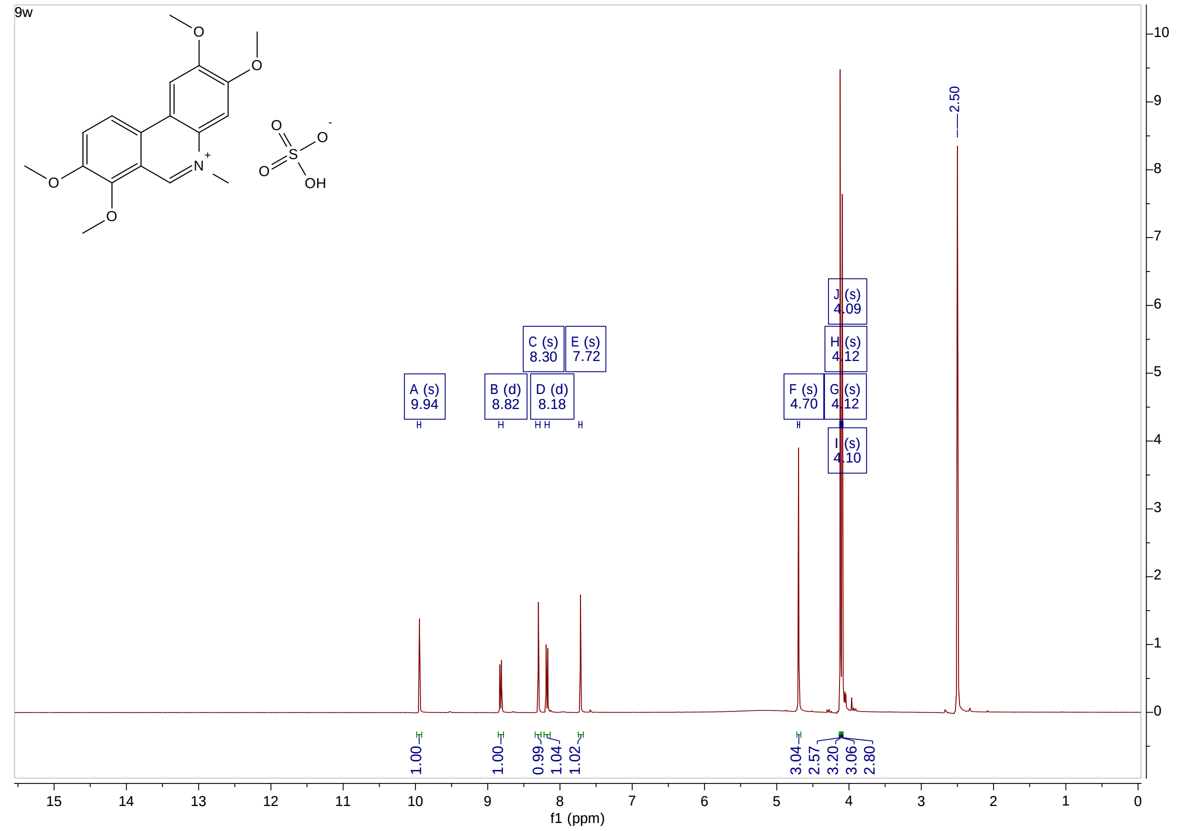

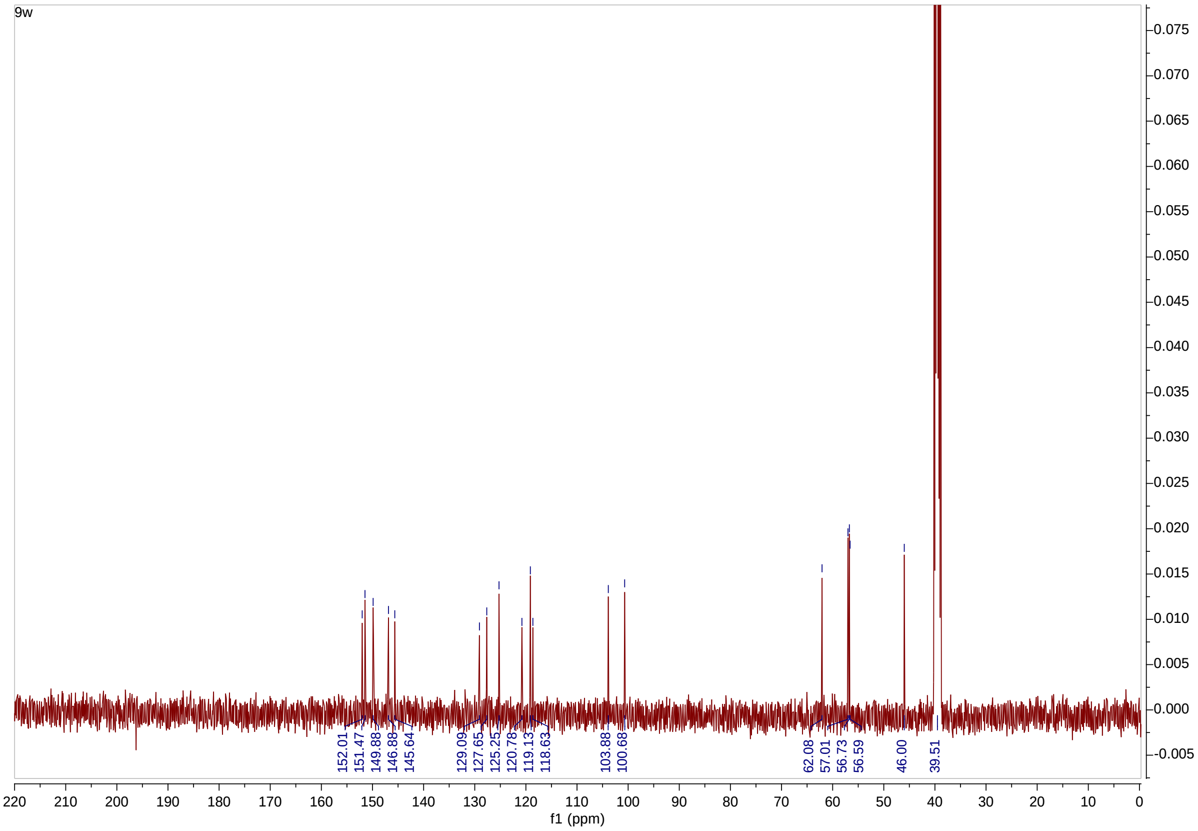

Compound 9z (DMSO-d_6)

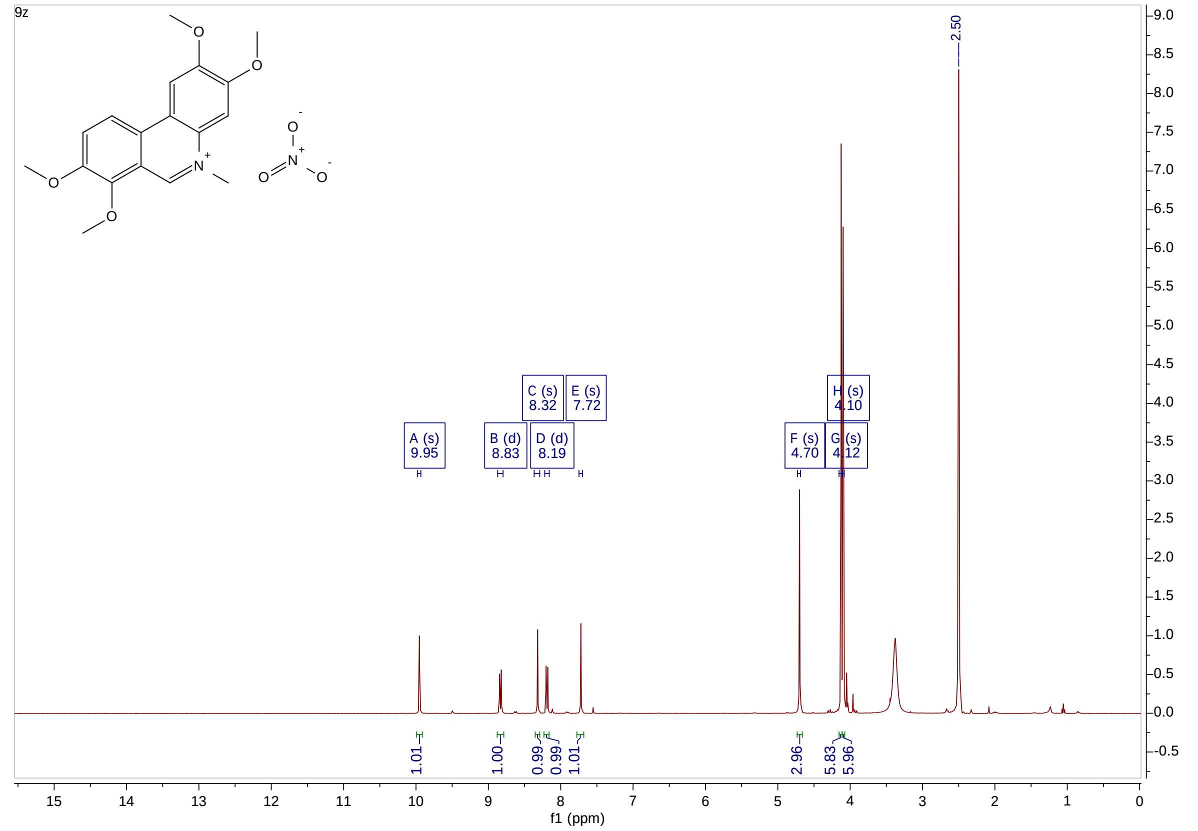

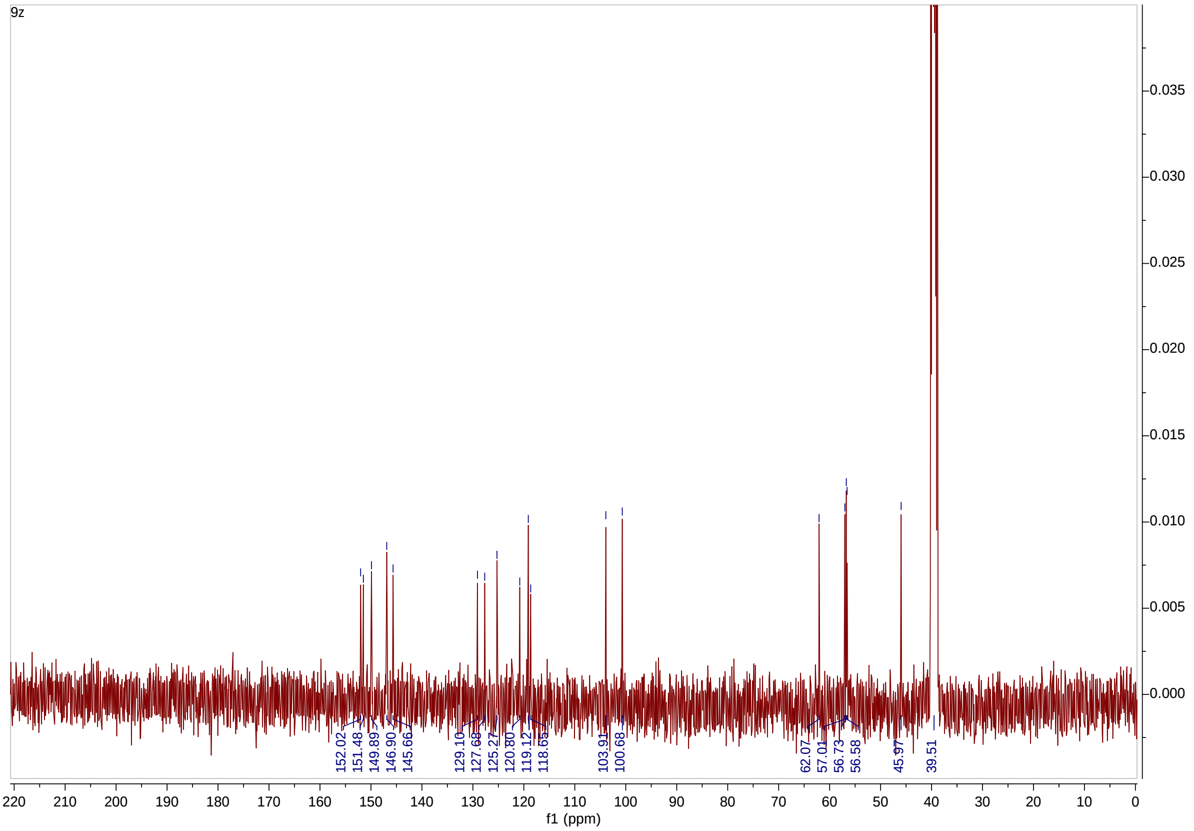
9z
152.02
151.48
149.89
146.90
145.66
129.10
127.68
125.27
120.80
119.12
118.65
103.91
100.68
62.07
57.01
56.73
56.58
45.97
39.51
f1 (ppm)
